# Advances in the engineering of living probiotics for cancer immunotherapy

**DOI:** 10.7150/thno.125301

**Published:** 2026-01-01

**Authors:** Sibtain Muhammad, Menglong Li, Qingyun Jia, Muhammad Ijaz, Shiqi Liang, Wenjun Zeng, Dongxiang Chen, Yinghe Zhang, Xuelian Du, Wencong Song, Bing Guo

**Affiliations:** 1 School of Science, Harbin Institute of Technology, Shenzhen 518055, China.; 2 Department of Radiology, Shenzhen Nanshan People's Hospital (NSPH), 89 Taoyuan Road, Nanshan District, Shenzhen 518052, China.; 3 Department of Radiology, Shenzhen People's Hospital (The Second Clinical Medical College, Jinan University; The First Affiliated Hospital, Southern University of Science and Technology), Shenzhen 518020, Guangdong, China.; 4 Shenzhen Key Laboratory of Advanced Functional Carbon Materials Research and Comprehensive Application, Harbin Institute of Technology, Shenzhen 518055, China.; 5 Department of Gynecology, Shenzhen Traditional Chinese Medicine Hospital, The Fourth Clinical Medical College of Guangzhou University of Chinese Medicine, No. 1, Fuhua Road, Futian District, Shenzhen 518033, China.

**Keywords:** engineered probiotics, cancer immunotherapy, tumor microenvironment, combination therapies, live probiotics

## Abstract

The role of bacteria in tumor development has been increasingly recognized through advances in sequencing technologies, revealing their influence on the tumor microenvironment and immune system. Live bacterial therapy, known for its unique ability to target tumors, colonize cancerous tissues, and activate immune responses, is emerging as a novel approach to cancer treatment. To enhance the therapeutic efficacy and safety of this strategy, various engineering techniques have been developed to modify bacteria, enabling the creation of advanced bacteria-based drug delivery systems. Living probiotics can selectively colonize the tumor microenvironment, where they interact with immune cells to enhance antitumor responses. This review provides an overview of the complex relationship between bacteria and tumors and discusses engineering methods for bacterial modification, including physicochemical approaches and synthetic biology. It further highlights the applications of these strategies in enhancing cancer therapies. Finally, it examines the future opportunities for engineered bacteria in cancer therapy, focusing on the potential of combination therapies, personalized medicine, and the role of the microbiome in enhancing therapeutic outcomes. With ongoing advancements, engineered bacteria hold great promise for improving the efficacy and safety of cancer treatments, offering a new frontier in oncology.

## Introduction

Cancer remains one of the leading causes of morbidity and mortality worldwide, despite substantial advancements in early detection, targeted therapies, and immune-based treatments 1. In recent years, immunotherapy has gained prominence as a revolutionary strategy that harnesses the body's immune system to recognize and destroy cancer cells. However, challenges such as tumor immune evasion, off-target effects, and limited delivery to tumor sites have prompted researchers to seek innovative and more precise therapeutic strategies. Among these, the use of living probiotics as engineered vehicles for cancer immunotherapy has gained significant attention due to their unique capabilities, including tumor targeting, immunomodulation, and biosynthetic versatility 2. Engineered probiotics are beneficial bacteria that have been modified to perform specific therapeutic functions against cancer. They can be designed to deliver drugs or therapeutic molecules directly to tumor sites, offering a targeted treatment approach that reduces side effects compared to conventional therapies. By leveraging their natural ability to survive and colonize within the body, these probiotics can improve the precision and effectiveness of cancer treatment. This innovative strategy combines microbiology and synthetic biology to develop new, safer, and more efficient cancer therapies 3.

Probiotics traditionally associated with gut health and disease prevention are live microorganisms that confer health benefits when administered in adequate amounts 4. With advances in synthetic biology and genetic engineering, these naturally occurring microbes are now being reprogrammed to function as therapeutic agents. Engineered probiotics can be tailored to deliver cytokines, checkpoint inhibitors, antigens, or other therapeutic molecules directly within the tumor microenvironment (TME), enhancing local immune activation while minimizing systemic toxicity 5, 6. Engineered probiotic strains have been shown to enhance antigen presentation by interacting with antigen-presenting cells (APCs) such as dendritic cells and macrophages. The bacteria's natural ability to modulate the gut microbiome and systemic immunity further supports their potential in cancer immunotherapy. Notably, several preclinical studies have demonstrated that probiotic-based vaccine platforms can generate robust antitumor immune responses. These findings highlight their potential as a promising alternative to conventional delivery methods.

The tumor microenvironment is often characterized by immunosuppressive conditions, poor oxygenation, and abnormal vasculature, which collectively hinder effective immune responses 7. Interestingly, certain bacterial species, including *Escherichia coli (E. coli) Nissle 1917*, *Lactobacillus spp.*, and *Bifidobacterium spp.*, have been shown to preferentially colonize tumors due to their ability to thrive in hypoxic and necrotic environments 8. Different engineering approaches for probiotic-based cancer therapy offer distinct advantages and limitations. Biological circuits enable precise control of therapeutic functions and immune modulation, but they may face challenges in stability, immunogenicity, and regulatory approval, affecting their clinical feasibility. In contrast, material-based delivery systems generally provide enhanced stability and controlled release of therapeutics, though they may lack the dynamic responsiveness of biological circuits. Similarly, various probiotic strains differ in their tumor colonization abilities and immune activation effects, with some strains showing superior targeting and immune stimulation but others offering better safety profiles. Compared to traditional drug delivery, engineered bacterial delivery offers improved tumor penetration and targeted therapy, potentially reducing systemic side effects and enhancing efficacy. However, challenges such as biosafety, control over bacterial activity, and large-scale clinical translation remain significant considerations 9. This natural tropism makes them excellent candidates for targeted drug delivery. Once inside the TME, engineered probiotics can act as “living factories,” producing therapeutic molecules *in situ*, modulating local immune cells, and triggering antitumor immunity 10.

In the past decade, synthetic biology has revolutionized our ability to design programmable bacteria with tightly regulated gene circuits, allowing precise spatiotemporal control of therapeutic payloads. These engineered microbes can be endowed with “sense-and-respond” systems, enabling them to detect specific tumor biomarkers or microenvironmental cues (such as hypoxia, pH, or inflammation) and initiate therapeutic responses accordingly 11. Furthermore, safety features such as kill-switches and biocontainment strategies have been incorporated to ensure clinical viability and regulatory compliance 12. Hybrid systems combining probiotics and nanomaterials represent a promising approach for cancer treatment by integrating the unique advantages of both components. Probiotics offer natural tumor-targeting abilities, biocompatibility, and the capacity to modulate the immune system, while nanomaterials provide high stability, controlled drug release, and multifunctionality. By combining these features, probiotic-nanomaterial hybrids can deliver therapeutic agents more efficiently to tumor sites, enhance immune responses, and improve treatment precision. This synergistic strategy holds great potential to overcome limitations of conventional therapies and offers a versatile platform for developing advanced, targeted cancer treatments 13.

Several preclinical studies and early-phase clinical trials have demonstrated the potential of engineered probiotics in combating various cancer types, including colorectal, breast, and melanoma. For instance, engineered *E. coli* strains expressing interleukin-2 (IL-2) or granulocyte-macrophage colony-stimulating factor (GM-CSF) have shown to stimulate robust immune responses and suppress tumor growth in mouse models 14. Other strategies involve engineering probiotics to direct tumor antigens, leading to antigen-specific T-cell responses, or to secrete anti-PD-L1 nanobodies, directly at the tumor site 15.

One of the most compelling aspects of probiotic-based cancer immunotherapy is its potential for personalization. By combining patient-specific tumor antigens with genetically modified probiotic carriers, researchers aim to develop individualized therapeutic regimens that elicit targeted immune responses with reduced adverse effects 16. Moreover, the integration of engineered probiotics with conventional therapies including chemotherapy, radiation, and immune checkpoint blockade offers synergistic benefits and may overcome resistance mechanisms that limit monotherapies 17. These include ensuring consistent colonization and persistence in tumors, preventing unintended immune reactions or horizontal gene transfer, and navigating the complex regulatory landscape for live biotherapeutic products 18. Additionally, interpatient variability in microbiota composition and immune status may influence the efficacy of probiotic-based therapies, underscoring the need for personalized approaches and robust preclinical models 19.

Recent advances in the engineering of living probiotics for cancer immunotherapy have focused on integrating targeted delivery, immune modulation, and controlled therapeutic release to overcome the limitations of conventional treatments. Early studies established the tumor-homing ability of certain probiotic strains, such as *E. coli* Nissle 1917 and *Bifidobacterium*, which provided a foundation for further engineering. Building on this, recent works have introduced innovations such as genetic circuits for controlled therapeutic expression, secretion of immune checkpoint inhibitors, and enzyme-mediated prodrug activation. These advances collectively enhance specificity, therapeutic efficacy, and safety. By linking these studies, it becomes clear that the field is moving towards multifunctional probiotic platforms capable of simultaneous tumor targeting, immune system activation, and reduced systemic toxicity, thereby opening new avenues for cancer immunotherapy 20.

This review offers new perspectives by integrating recent advances in engineering living probiotics specifically for cancer immunotherapy, a topic that has not been comprehensively addressed in prior reviews. It highlights innovative strategies such as AI-driven synthetic biology design, hybrid probiotic nanomaterial systems, and imaging-guided delivery, which represent cutting-edge directions in the field. Additionally, the review provides a comparative analysis of different engineering approaches, probiotic strains, and therapeutic modalities, offering a unified framework that can guide future research and accelerate clinical translation.

This review aims to provide a comprehensive overview of recent advances in the engineering of living probiotics for cancer immunotherapy. Living probiotics offer unique advantages for cancer immunotherapy, including natural tumor targeting, immune modulation, and on-site therapeutic production. Compared to other living cell-based therapies and bacterial outer membrane vesicle (OMV)-based approaches, they provide simpler administration and sustained activity but face challenges in biosafety, control, and regulatory approval. While each approach has distinct strengths, living probiotics represent a promising, versatile, and cost-effective platform for advancing cancer immunotherapy. We begin by exploring the fundamental biology of probiotic-tumor interactions and the rationale for their use in cancer treatment. We then examine the latest strategies for engineering probiotics, including gene circuit design, payload optimization, and delivery systems. Furthermore, we highlight preclinical and clinical studies that demonstrate the feasibility and efficacy of this approach. Finally, we discuss current challenges, regulatory considerations, and future directions for translating probiotic-based immunotherapy into mainstream cancer treatment. The engineering of living probiotics for cancer immunotherapy represents a paradigm shift in the way we think about microbial therapeutics and oncology. As synthetic biology tools continue to evolve, and as our understanding of host-microbe-tumor interactions deepens, engineered probiotics may soon become a cornerstone of personalized and precise cancer immunotherapy.

While existing reviews have explored various aspects of probiotic-based cancer therapies, they often focus on specific modalities or mechanisms, such as immune modulation or tumor targeting. Our manuscript provides a comprehensive synthesis of the latest engineering strategies, including AI-driven design, synthetic biology, and multimodal delivery systems, offering a holistic perspective on the advancements in this field. By integrating these diverse approaches, we aim to present a unified framework that highlights the synergistic potential of engineered probiotics in cancer immunotherapy.

## 2. The Tumor Microenvironment and Microbial Interactions

The tumor microenvironment (TME) is a complex and dynamic ecosystem that plays a pivotal role in cancer progression, immune evasion, and resistance to therapy. The tumor microenvironment (TME) consists of cancer cells, various immune and stromal cells, and interconnected signaling molecules, characterized by distinctive conditions including oxygen deprivation, acidic pH, and suppressed immune activity. These characteristics not only promote tumor survival but also hinder the efficacy of conventional and immune-based therapies 21, 22. A growing body of research has focused on exploiting these features for therapeutic gain, particularly through the use of microbes that naturally thrive or can be engineered to function in such hostile conditions.

**Figure 1:**
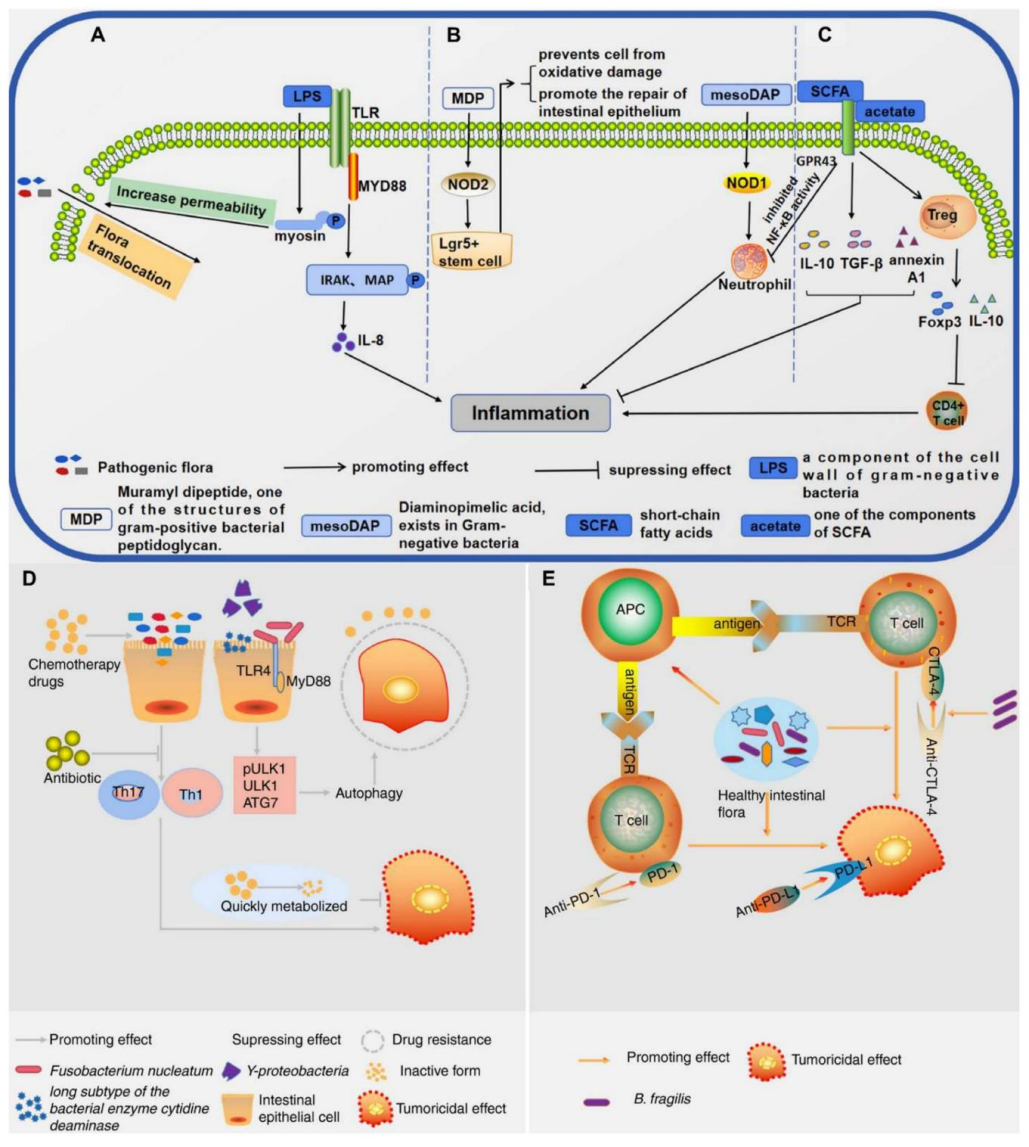
Interconnections between gut microbial components, their metabolites, and host immunity: **(A)** Lipopolysaccharides (LPS) trigger inflammatory responses by activating the TLR-MYD88 signaling cascade, leading to downstream phosphorylation events. **(B)** Muramyl dipeptide (MDP) supports the viability of Lgr^5+^ stem cells located in the intestinal crypts via NOD2 signaling, protecting them from injury and facilitating epithelial repair. Additionally, meso-diaminopimelic acid (meso-DAP) activates NOD pathways, enhancing the antimicrobial function of bone marrow-derived neutrophils and intensifying inflammatory responses. **(C)** Short-chain fatty acids (SCFAs), particularly via the GPR43 receptor, stimulate regulatory T cells (Tregs), increasing their population and promoting the expression of immunoregulatory markers such as *Foxp3* and *IL-10*. This, in turn, suppresses the activity of effector CD4+ T cells and alleviates colitis symptoms. **(D)** Influence of gut microbiota on resistance to cancer therapies. **(E)** Role of gut microbiota in modulating immunotherapeutic responses. A healthy intestinal microbiota can stimulate antigen-presenting cells (APCs). Reused under Creative Commons Attribution License from ref. 23.

Yang *et al.* 2021 explained in his study that intestinal microbiota plays a pivotal role in shaping host immune responses and has profound implications for cancer development and therapy. The tumour microenvironment is a complex milieu consisting of immune cells, stromal cells, and extracellular matrix components. The gut microbiota, by modulating systemic and local immune responses, can influence whether a tumour exhibits an immunosuppressive or immunostimulatory microenvironment. Commensal microbes impact both innate and adaptive immunity. Certain beneficial species, such as *Akkermansia muciniphila*, *Bacteroides fragilis*, and *Lactobacillus reuteri*, enhance the recruitment and activation of dendritic cells, macrophages, and T cells, thereby promoting anti-tumour immunity. Microbes generate metabolites like short-chain fatty acids (SCFAs), which modulate the differentiation of regulatory and effector T cells, thereby helping maintain immune balance and supporting anti-tumor immunity. Conversely, dysbiosis characterized by an imbalance in microbial composition can facilitate tumour growth and immune evasion. Pathogenic bacteria such as *Fusobacterium nucleatum* and *Helicobacter hepaticus* have been linked with chronic inflammation, DNA damage, and immunosuppression within the TME. These changes contribute to tumour initiation and progression in various cancers, including colorectal, liver, and pancreatic cancers **(Figure 1)**. Patients with a favourable microbiota profile often show better responses to ICIs, suggesting the potential of microbiota-based strategies to enhance immunotherapy efficacy. Modulating the microbiota could convert cold tumours into hot ones, making them more amenable to immunotherapy 23.

Hypoxia, a hallmark of the TME, arises from abnormal tumor vasculature and rapid cellular proliferation, resulting in areas of low oxygen tension that are inaccessible to most immune cells and drugs. Interestingly, several bacterial species, especially obligate and facultative anaerobes, are naturally drawn to and capable of colonizing these hypoxic tumor regions. *Clostridium novyi*-NT, a genetically modified obligate anaerobe, was among the first bacterial strains shown to selectively germinate in the hypoxic cores of tumors and mediate lysis of malignant tissues in preclinical models 24. Likewise, attenuated and genetically modified *Salmonella typhimurium* exhibits natural tumor-targeting properties and has been shown to trigger immunogenic cell death while activating both innate and adaptive immune responses 25.

In addition to pathogenic bacteria, certain probiotic strains also display intrinsic tumor-targeting capabilities. For instance, *E. coli* Nissle 1917, *Lactobacillus* spp., and *Bifidobacterium* spp. have been reported to localize within tumor tissues following oral or systemic administration, particularly in conditions where the mucosal barrier is disrupted by inflammation or cancer therapy 26. *Bifidobacterium longum* has been successfully used to deliver therapeutic payloads directly to tumors. In one study, it was engineered to express cytosine deaminase, an enzyme that converts the non-toxic prodrug 5-fluorocytosine (5-FC) into the active chemotherapeutic agent 5-fluorouracil (5-FU), enabling targeted drug activation within the tumor microenvironment 27. This form of microbial-mediated prodrug therapy represents a promising strategy to limit systemic toxicity while enhancing local anti-tumor efficacy.

The interaction between microbes and the host immune system is a critical component of their anti-cancer potential. Bacteria possess pathogen-associated molecular patterns (PAMPs) such as lipopolysaccharide (LPS) and peptidoglycans that are recognized by pattern recognition receptors (PRRs) like Toll-like receptors (TLRs) on immune cells. Activation of these receptors can lead to the maturation of dendritic cells, the production of pro-inflammatory cytokines, and the recruitment of cytotoxic T lymphocytes to the tumor site 28. For instance, in a mouse model of breast cancer, oral delivery of *Lactobacillus casei* resulted in notable tumor reduction and increased infiltration of CD8+ T cells, highlighting the immune-boosting potential of probiotic treatment 29. Similarly, *E. coli* Nissle has been bioengineered to produce interleukin-2 (IL-2), a cytokine that stimulates the activation of T cells and natural killer (NK) cells. In mouse models, this approach resulted in robust immune activation and considerable tumor shrinkage without systemic toxicity 30.

Beyond exogenous administration of probiotics, the endogenous tumor-associated microbiota itself has emerged as a key modulator of cancer progression and immune responses. Microorganisms such as Fusobacterium nucleatum, often found in colorectal cancers, have been shown to promote tumor growth, confer resistance to chemotherapy, and facilitate immune evasion through mechanisms involving TLR activation and the modulation of immune checkpoints 31. Conversely, the presence of beneficial commensals such as Akkermansia muciniphila has been positively correlated with improved responses to immune checkpoint inhibitors in melanoma patients, suggesting that microbial composition may influence the success of immunotherapy 32.

**Figure 2:**
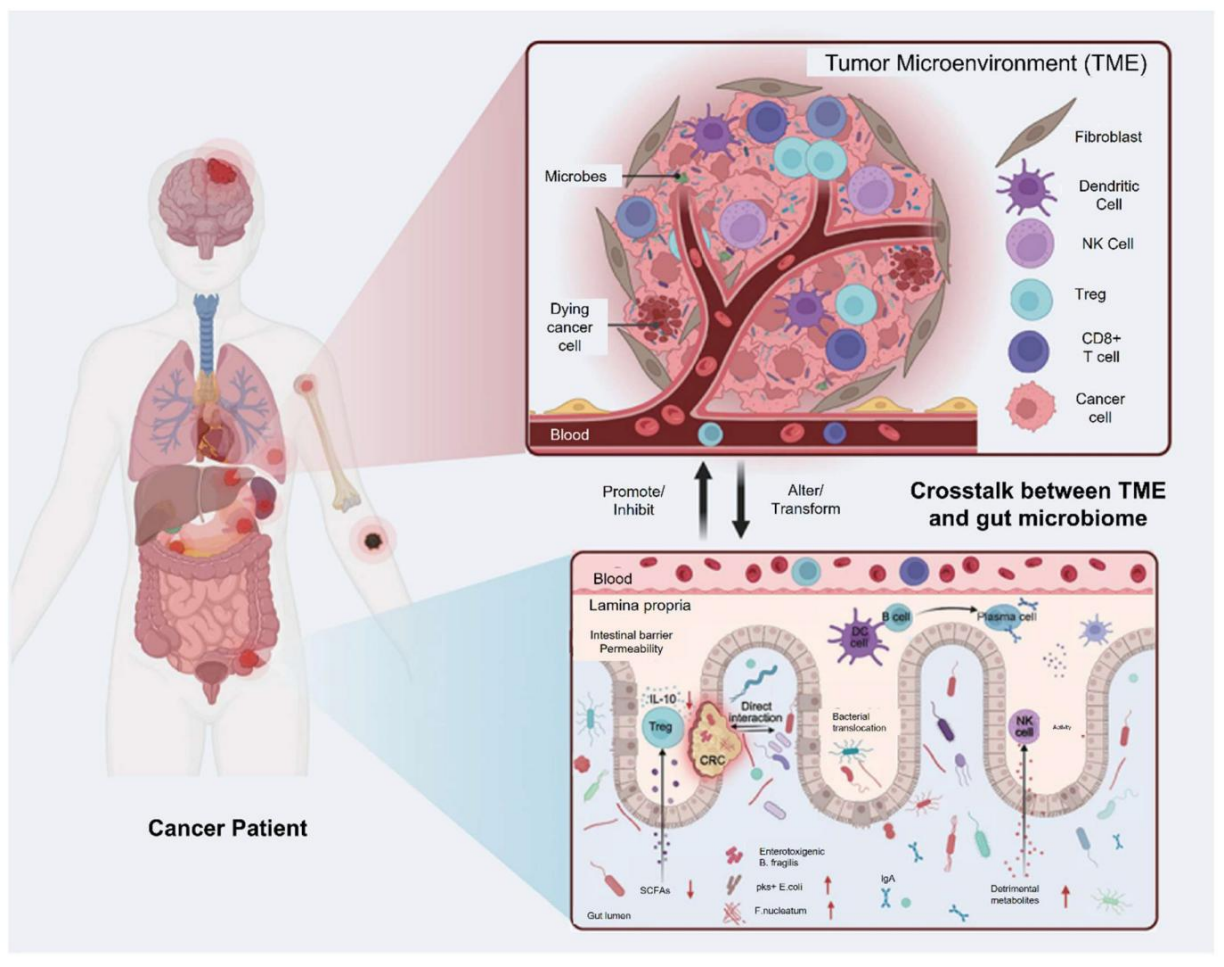
The interaction between the gut microbiome and the tumor microenvironment (TME). Reused under Creative Commons Attribution License from ref. 33.

Jiang* et al.* 2024 explores in his study the complex and bidirectional relationship between gut microbiota and cancer, emphasizing how disruptions in microbial balance can influence tumor development and progression. They highlight emerging evidence that the gut microbiota significantly shapes the tumor microenvironment (TME), affecting both local and distant tumors. Furthermore, the microbiota's role in modulating the outcomes of cancer immunotherapy is underscored, as it can impact therapeutic specificity and long-term success across various cancer types. They particularly focus on probiotics as promising agents in cancer treatment due to their ability to beneficially modulate gut microbiota and enhance immune responses. Probiotics may synergize with immunotherapy by stimulating the host's immune system to suppress tumor growth. The study presents recent scientific advances and mechanisms through which probiotics can improve the efficacy of immunotherapeutic strategies **(Figure 2)**. Overall, they provide a comprehensive overview of how probiotics and gut microbiota interactions influence cancer biology and therapy, suggesting that harnessing these interactions could offer innovative pathways for cancer diagnosis, treatment, and immunotherapy optimization. Understanding these relationships may pave the way for more personalized and effective cancer interventions by integrating microbiome-targeted strategies alongside conventional therapies 33.

TME is a complex network of cancer cells, immune cells, blood vessels, and extracellular matrix that supports tumor growth and progression. Microbial interactions within the TME can influence tumor development by modulating immune responses, metabolism, and inflammation. Understanding these interactions offers new opportunities for developing targeted cancer therapies that exploit the relationship between microbes and the TME.

**Figure 3:**
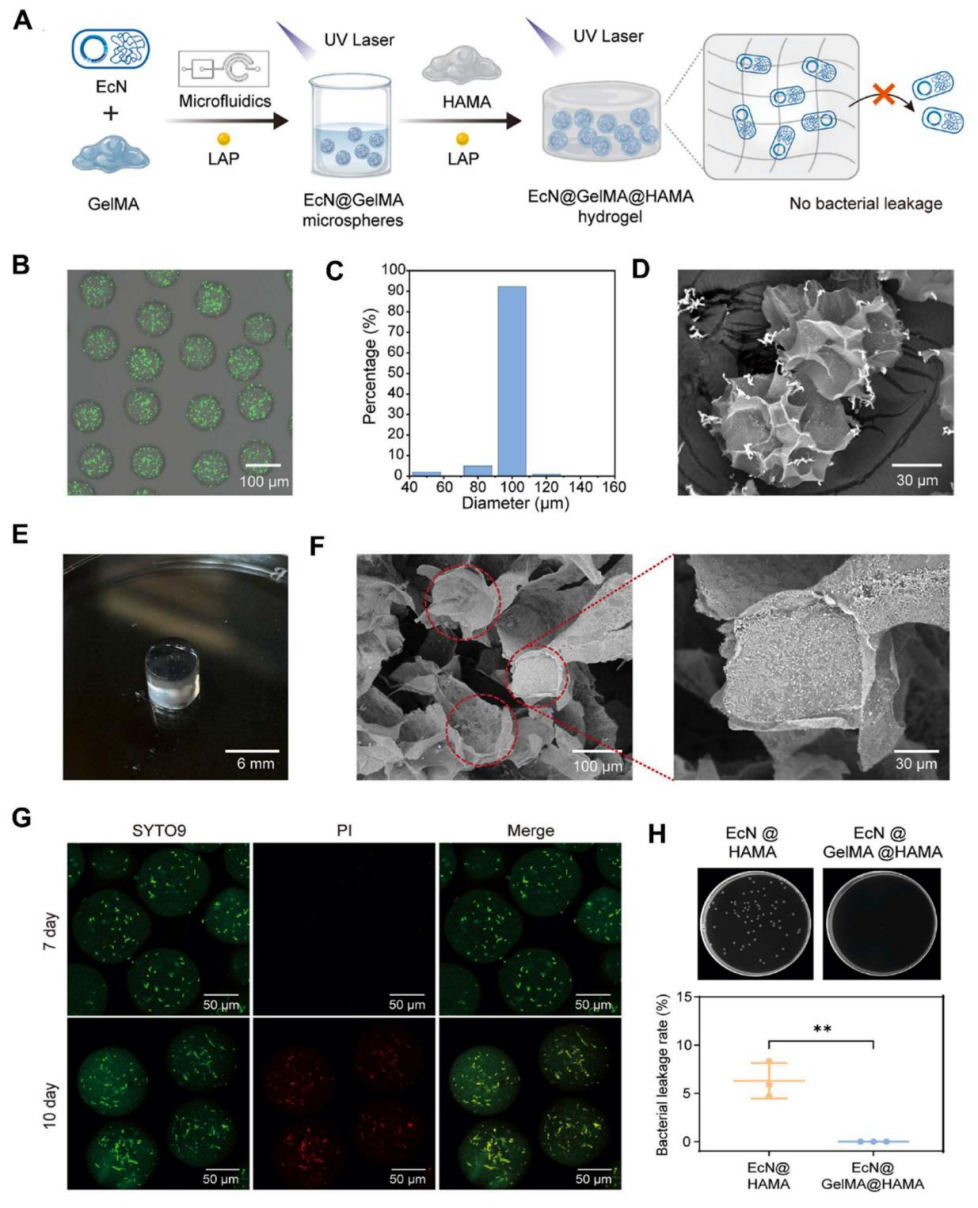
Preparation and characterization of the probiotic-enabled living hydrogel: **(A)** A schematic representation illustrates the step-by-step fabrication of the living hydrogel construct. Initially, engineered bacteria were gently mixed into a 2.5% (w/v) GelMA solution to create a precursor mixture with an optical density (OD) of 1. This suspension was then processed into microspheres loaded with bacteria using a microfluidic device. These bacteria-containing microspheres were subsequently combined with a 3% (w/v) HAMA solution to form a bulk hydrogel. Both the microspheres and the final hydrogel structure were stabilized through photocrosslinking. **(B)** Images show typical appearances of the EcN@GelMA microspheres. **(C)** The size distribution of the EcN@GelMA microspheres is presented in histogram form. **(D)** Scanning electron microscopy (SEM) images provide detailed views of the microsphere morphology. **(E)** A digital photograph displays the final EcN@GelMA@HAMA living hydrogel. **(F)** SEM images reveal the internal structure of the EcN@GelMA@HAMA hydrogel, with samples freeze-dried prior to imaging. **(G)** Live/dead staining was used to assess bacterial viability inside the hydrogel system (Live cells appear green with SYTO9; dead cells appear red with PI). **(H)** A bacterial leakage assay was conducted to evaluate the hydrogel's ability to contain bacteria over a 48-hour incubation. The leakage percentage was 6.3% for the HAMA-only hydrogel and 0% for the GelMA/HAMA composite hydrogel. Supporting images above the bar graph show bacterial colonies that grew after plating supernatant samples (diluted 10,000-fold) on LB agar. Reused under Creative Commons Attribution License from ref 34.

Fang* et al.* 2025, presents a novel approach to bone tissue regeneration by integrating genetically engineered probiotics into a responsive biomaterial system. The study addresses the limitations of traditional bone grafting techniques, which often face issues like immune rejection, infection risk, and poor integration. To overcome these challenges, the authors propose a Living Responsive Regenerative Medicine (LRRM) strategy that utilizes the body's natural signaling mechanisms specifically elevated nitric oxide (NO) levels that occur during bone fracture as a biological trigger for therapeutic action. At the core of this strategy is the use of *E. coli* Nissle 1917 (EcN), a well-characterized probiotic strain. This bacterium was genetically modified to detect increased NO levels in the fracture microenvironment and respond by producing and secreting bone morphogenetic protein-2 (BMP2), a potent factor known to stimulate bone formation. This allows for a real-time and site-specific release of therapeutic proteins based on the body's own signals, ensuring localized and timely healing responses. To deliver these engineered bacteria safely and effectively, the researchers developed a dual-encapsulation system. First, the modified EcN bacteria were encapsulated in gelatin methacryloyl (GelMA) microspheres. These microspheres offer a supportive matrix and act as the first protective layer. They were then embedded within a larger hyaluronic acid methacryloyl (HAMA) hydrogel scaffold, forming a bilayer structure. This encapsulation system plays a vital role in preventing immune system detection and bacterial leakage, which are major safety concerns in microbial-based therapies. The hydrogel not only secures the engineered bacteria in place but also provides a biomimetic environment that supports cellular interactions and tissue integration. *In vivo* experiments using multiple animal models with bone defects demonstrated the therapeutic effectiveness of this living hydrogel. The LRRM platform significantly enhanced bone callus formation, promoted neovascularization (formation of new blood vessels), and facilitated full-thickness bone healing (**Figure 3**). These outcomes indicate that the therapy supports not only bone regeneration but also vascular integration, which is crucial for the long-term viability and function of regenerated tissue. The strategic design of combining synthetic biology with advanced biomaterial engineering highlights the potential of living therapeutics in regenerative medicine. The use of a biological signal (NO) to autonomously trigger therapeutic activity, along with a dual-encapsulation method for controlled and safe delivery, represents a major advancement in the field. This system offers a high degree of tunability, responsiveness, and safety, which are essential for future clinical applications 34.

## 3. Probiotics as Therapeutic Agents: From Gut Health to Cancer Therapy

Probiotics are live microorganisms that, when consumed in adequate amounts, provide health benefits to the host and have traditionally been associated with maintaining gut health. They have been widely used to restore intestinal balance, strengthen immune function, and manage gastrointestinal conditions such as irritable bowel syndrome, inflammatory bowel disease (IBD), and antibiotic-associated diarrhea 35. Recently, interest has expanded toward their potential role in cancer therapy. Probiotics are now being explored for their capacity to modulate immune responses, improve the effectiveness of existing cancer treatments, and directly interact with tumor cells. Advances in biotechnology have further enabled the engineering of probiotics to deliver therapeutic agents, modify the tumor microenvironment (TME), and enhance immune surveillance key mechanisms in the fight against cancer.

**Figure 4:**
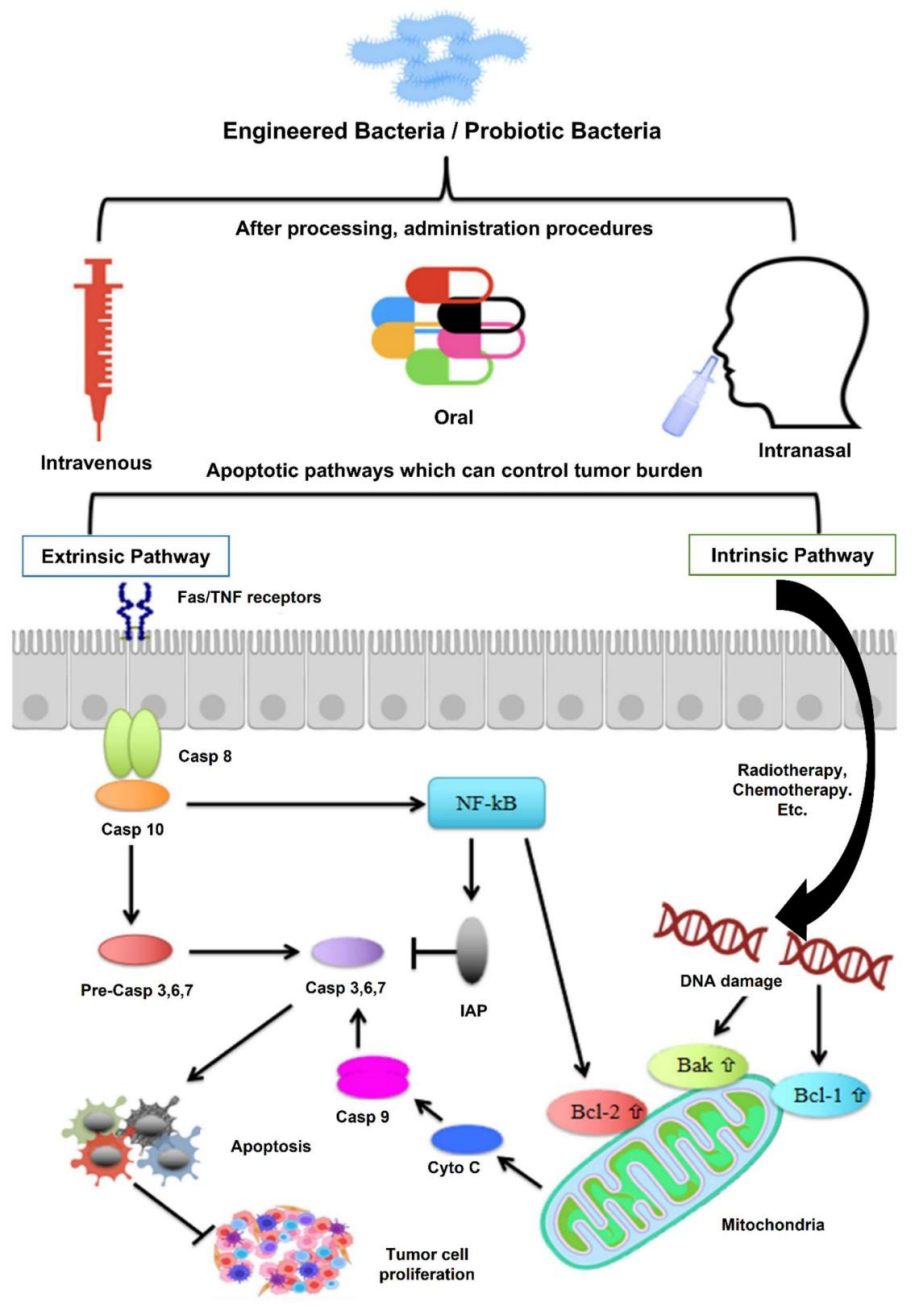
Mechanism of action: probiotics and tumor suppression via apoptotic pathways. Probiotics can influence tumor suppression by modulating both extrinsic and intrinsic apoptotic mechanisms. In the extrinsic pathway, FAS ligand activates death receptors on the cell surface, initiating programmed cell death. The intrinsic pathway, typically triggered by internal stimuli like chemotherapy or radiation, leads to DNA damage that increases the expression of mitochondrial proteins Bax and Bak. These proteins facilitate the release of cytochrome c, which activates caspase-dependent apoptosis. Additionally, probiotics may inhibit tumor growth by downregulating NF-κB activity and its downstream pro-survival gene expression. Reused under Creative Commons Attribution License from ref. 36.

Mukherjee* et al.* 2024 reported a study in which they explore the potential of probiotics as supportive or alternative agents in cancer therapy, focusing on their underlying mechanisms and practical applications. Probiotics beneficial live microorganisms primarily found in the gut have demonstrated anti-tumor effects through various biological activities, making them promising candidates in integrative cancer treatment strategies. Probiotic strains like *Lactobacillus* and *Bifidobacterium* have shown the ability to inhibit tumor growth by triggering apoptosis (programmed cell death), inhibiting oncogene expression, and altering cytokine profiles to favor anti-tumor immunity. Moreover, the authors highlight that probiotics can positively influence the tumor microenvironment (TME), making it less conducive to cancer progression. By rebalancing dysbiosis (microbial imbalance), probiotics reduce chronic inflammation and oxidative stress two major contributors to carcinogenesis. Additionally, they can enhance the effectiveness of existing treatments such as chemotherapy and immunotherapy by improving drug metabolism, reducing side effects, and enhancing the host's immune response. They also discuss the role of probiotics in reducing therapy-associated complications, such as mucositis, diarrhea, and immunosuppression, commonly seen during chemotherapy and radiation **(Figure 4)**. These benefits make probiotics attractive not only for therapeutic purposes but also for improving the quality of life of cancer patients 36.

The most well-established use of probiotics lies in maintaining gut health. The human gastrointestinal tract is home to a complex and diverse microbiota that plays a critical role in digestion, nutrient absorption, and immune system regulation. Probiotics help maintain microbial balance by preventing the overgrowth of pathogenic microorganisms, promoting beneficial bacteria, and enhancing the integrity of the intestinal epithelial barrier 37. This gut microbiota is intimately connected to the host's immune system, and alterations in its composition, known as dysbiosis, have been linked to various health conditions, including cancer, autoimmune diseases, and inflammatory conditions 38.

Probiotics exert their effects on the immune system through various mechanisms. For example, they can stimulate the production of cytokines, activate antigen-presenting cells (APCs) such as dendritic cells, and enhance the function of both innate and adaptive immune cells 39. These immune-modulating properties of probiotics are critical not only for maintaining gut homeostasis but also for influencing the systemic immune response. In the context of cancer, a robust immune system is essential for recognizing and eliminating tumor cells. Studies have demonstrated that probiotics can strengthen immune surveillance by stimulating the activation and proliferation of effector T cells, natural killer (NK) cells, and macrophages, all of which play critical roles in identifying and eliminating cancer cells 40.

The idea of leveraging probiotics for cancer therapy stems from the emerging understanding that the microbiome and its associated immune responses significantly influence cancer progression and response to treatment 41. Tumor immunology has revealed the central role of the tumor microenvironment (TME) in dictating the success or failure of cancer therapies, including immunotherapy, chemotherapy, and radiation therapy. The TME is typically characterized by a pro-inflammatory milieu, hypoxia, and immune suppression, which can hinder the effectiveness of therapeutic interventions 42. Interestingly, the gut microbiota can influence the TME by modulating systemic immune responses, suggesting that probiotics might not only protect against gastrointestinal disturbances but could also serve as tools for enhancing anti-tumor immunity.

Research indicates that the microbiota can influence tumor progression and metastasis by regulating immune checkpoint activity and modulating inflammatory signaling pathways. For instance, the gut microbiota has been linked to the effectiveness of immune checkpoint inhibitors (ICIs) in cancer therapy. In particular, *Bacteroides fragilis*, *Firmicutes*, and *Akkermansia muciniphila* have been associated with improved responses to PD-1 blockade in melanoma and lung cancer patients 43. The precise mechanisms by which the microbiota modulates anti-tumor immunity are still under investigation, but it is clear that probiotics can influence immune checkpoint activity, which has profound implications for cancer immunotherapy.

One of the most exciting developments in the field of cancer immunotherapy is the engineering of probiotics to deliver therapeutic agents directly to tumor sites. Genetically modified probiotics can be designed to release targeted cytokines, enzymes, or immune-regulating molecules that reshape the tumor microenvironment (TME), activate anti-tumor immune mechanisms, and improve the efficacy of standard cancer treatments. The versatility of synthetic biology tools, such as CRISPR/Cas9, has opened new avenues for designing probiotics with tailor-made functionalities to treat cancer. For example, *Lactobacillus casei*, a commonly used probiotic, has been engineered to express interleukin-2 (IL-2), a cytokine known to promote the activation and expansion of cytotoxic T lymphocytes (CTLs). In preclinical studies, IL-2-producing *L. casei* strains have been shown to enhance anti-tumor immunity and inhibit the growth of both primary and metastatic tumors in mouse models 44. This strategy capitalizes on the ability of probiotics to modulate the immune system locally, directly at the site of the tumor, without the systemic side effects typically associated with cytokine therapies.

In addition to cytokine delivery, probiotics can also be engineered to express enzymes that activate prodrugs in the TME. Prodrug systems are designed to minimize systemic toxicity by delivering therapeutics specifically to tumor cells. For example, *E. coli* Nissle 1917, a well-characterized probiotic strain, has been engineered to express cytosine deaminase (CD), an enzyme that converts the non-toxic prodrug 5-fluorocytosine (5-FC) into the potent chemotherapeutic agent 5-fluorouracil (5-FU). When administered to tumor-bearing mice, the engineered *E. coli* strain efficiently converted 5-FC to 5-FU *in situ*, resulting in significant tumor regression 45. This approach highlights the potential of probiotics as delivery vehicles for therapeutic enzymes that can locally activate cancer treatments.

Probiotics hold promise not only as standalone therapeutic agents but also in combination with other cancer treatments, such as chemotherapy, radiation therapy, and immune checkpoint blockade. One of the key advantages of probiotics is their ability to enhance the body's immune response and promote systemic anti-tumor immunity, thereby amplifying the effects of existing therapies. Recent studies have shown that probiotics can enhance the efficacy of chemotherapy by improving the host's immune system and gut integrity. For example, *Lactobacillus rhamnosus* has been shown to alleviate chemotherapy-induced intestinal injury and enhance the immune response to tumors in mice 46. By maintaining the integrity of the intestinal barrier and promoting the expansion of beneficial gut microbiota, probiotics can improve the systemic delivery and effectiveness of chemotherapy agents.

In addition to chemotherapy, probiotics can also synergize with radiation therapy. Radiation therapy often induces significant damage to the normal tissue surrounding the tumor, leading to inflammation, immune suppression, and increased susceptibility to infection. Probiotics, by modulating the immune response, can mitigate these side effects and enhance the therapeutic index of radiation therapy. For example, *Lactobacillus plantarum* has been shown to reduce radiation-induced gut damage in preclinical models, improving the overall response to combined radio- and immunotherapy 47.

The combination of probiotics with immune checkpoint inhibitors (ICIs) represents another promising strategy in cancer treatment. By modulating the microbiome and enhancing anti-tumor immunity, probiotics can improve the response to ICIs such as anti-PD-1 or anti-CTLA-4 antibodies. In a study on melanoma patients, the presence of certain gut microbiota, including *Faecalibacterium prausnitzii* and *Akkermansia muciniphila*, was associated with improved responses to PD-1 blockade 48. The ability of probiotics to modulate the microbiome, influence immune checkpoints, and enhance the efficacy of ICIs could lead to better clinical outcomes in cancer patients. While the potential of probiotics in cancer therapy is promising, there are several challenges that need to be addressed. First, the variability in the composition of the human microbiome means that not all patients will respond similarly to probiotic-based therapies. The individual microbiome composition could influence the success of probiotic interventions, making it important to identify biomarkers that can predict which patients will benefit from probiotic-based therapies 49.

Second, the safety of engineered probiotics must be carefully evaluated. While probiotics are generally considered safe, the engineering of microbes to express therapeutic agents raises concerns about unintended effects, such as horizontal gene transfer, pathogen evolution, or systemic toxicity. Rigorous preclinical testing and the development of biosafety mechanisms, such as kill switches and self-regulation systems, will be essential to ensure the safe use of engineered probiotics in cancer patients 50. Finally, regulatory hurdles remain a significant challenge in the clinical translation of probiotic-based cancer therapies. The approval of genetically engineered probiotics for clinical use requires extensive validation of their safety and efficacy. Regulatory bodies like the FDA and EMA must develop well-defined guidelines for incorporating live therapeutics into cancer treatment, encompassing standardized requirements for manufacturing practices and clinical trial protocols 51.

Probiotics represent a new frontier in cancer therapy, offering innovative ways to modulate the immune system, enhance the efficacy of existing treatments, and directly target tumor cells. Through engineering, probiotics can be programmed to deliver therapeutic agents such as cytokines, enzymes, and immune-modulatory molecules directly to tumor sites, providing a targeted and less toxic alternative to conventional cancer therapies.

## 4. Engineering Strategies for Living Probiotics

**Figure 5:**
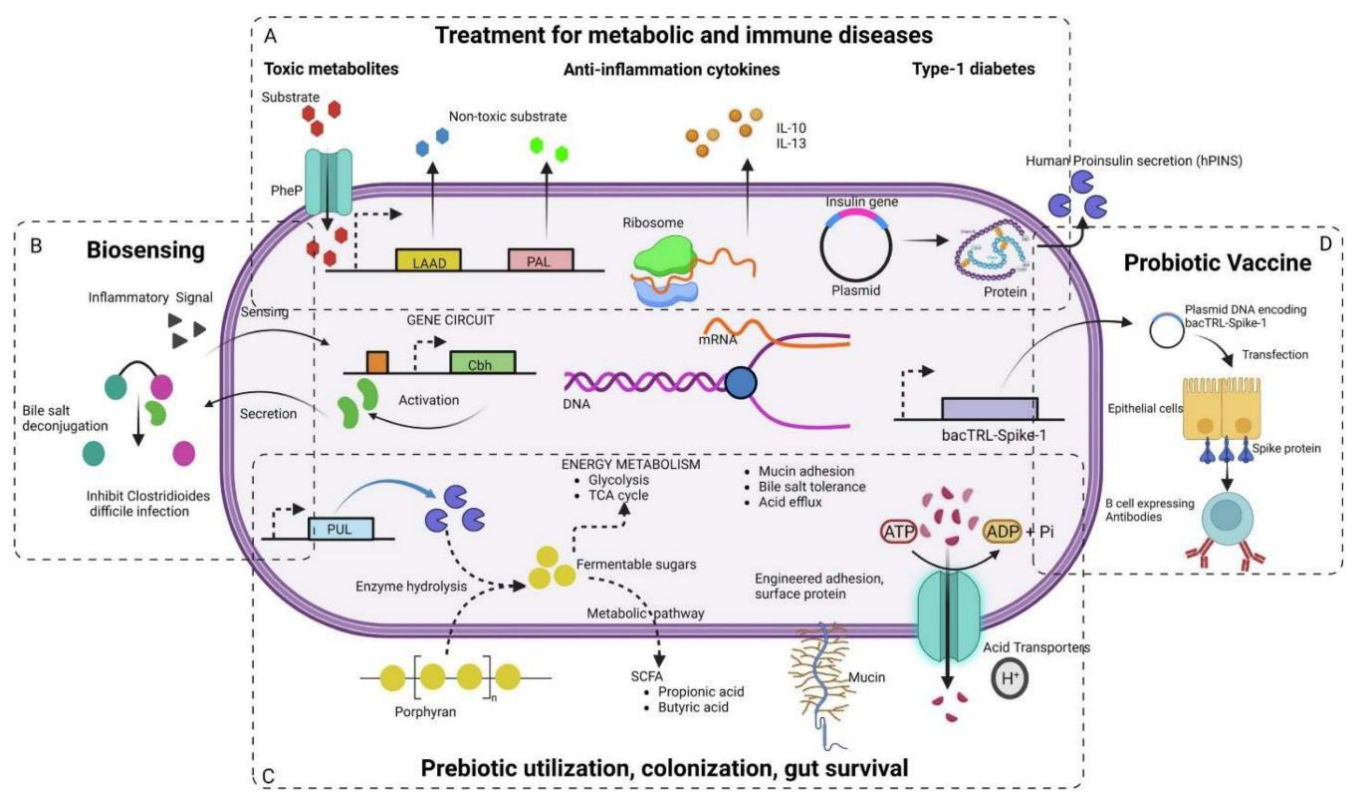
Various synthetic biology approaches in engineering next-generation probiotics for the treatment of **(A)** Metabolic and immune diseases, **(B)** biosensing and diagnostics, **(C)** prebiotic usage and gut colonization **(D)** vaccine delivery. Reused under Creative Commons Attribution License from ref. 62.

The genetic engineering of living probiotics has become a promising strategy to boost their therapeutic efficacy, especially in the field of cancer immunotherapy. Traditionally, probiotics have been used as dietary supplements to improve gut health and modulate immune responses **(Figure 5)**. However, with advances in genetic engineering, it is now possible to modify these microorganisms to perform more specific, targeted functions within the host. These engineered probiotics have been designed to deliver therapeutic agents, modulate the immune system, and enhance the effectiveness of existing cancer treatments 61.

A key objective in the development of engineered probiotics is to enhance their capacity to engage with the host's immune system effectively. Research has demonstrated that probiotics can activate immune responses by stimulating different immune cells such as T cells, natural killer (NK) cells, and dendritic cells, all of which are essential in identifying and destroying cancerous cells. Through genetic engineering, probiotics have been modified to produce cytokines—molecules that regulate immune functions. For example, the probiotic strain *Lactobacillus casei* has been altered to secrete interleukin-2 (IL-2), a cytokine known for its role in encouraging the growth and activity of T cells. Studies using mouse models have shown that these IL-2-producing *L. casei* strains improve anti-cancer immune responses by strengthening the ability of T cells to target and kill tumor cells. This illustrates the potential of genetically enhanced probiotics to support immune-based cancer therapies.

Another approach to engineering probiotics involves the modification of their metabolic pathways to produce therapeutic molecules that directly affect the tumor microenvironment (TME). The TME is known to be immunosuppressive, with factors such as low oxygen levels, metabolic waste products, and regulatory immune cells limiting the effectiveness of traditional cancer treatments. By engineering probiotics to secrete enzymes or other bioactive molecules that alter the TME, it is possible to enhance anti-tumor immunity. One example of this is the engineering of *E. coli* Nissle 1917, a strain of bacteria commonly used as a probiotic, to express the enzyme cytosine deaminase (CD). CD catalyzes the conversion of the prodrug 5-fluorocytosine (5-FC) into the cytotoxic chemotherapeutic agent 5-fluorouracil (5-FU). When administered in combination with 5-FC, *E. coli* Nissle 1917 significantly reduces tumor growth in mouse models, demonstrating the potential of engineered probiotics to directly deliver chemotherapy agents to tumors 64. This strategy minimizes systemic toxicity by localizing the production of the active drug within the tumor site.

**Figure 6:**
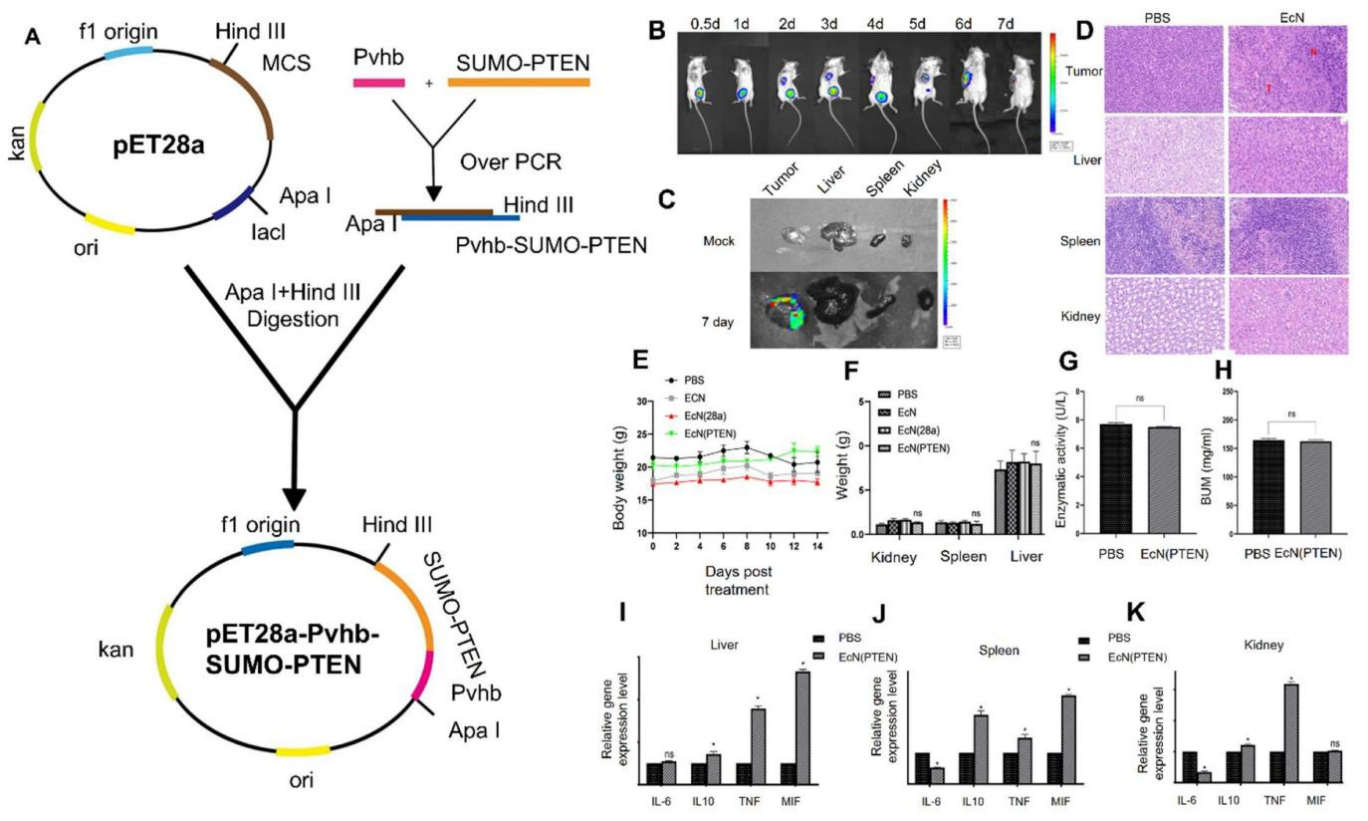
Construction and characterization of engineered *E. coli* Nissle 1917 (EcN) expressing PTEN: **(A)** A schematic map of the PTEN expression vector was designed, incorporating the hypoxia-inducible promoter Pvhb to ensure selective expression in tumor environments. **(B)** *In vivo* imaging system (IVIS) was used to monitor the colonization of luminescent EcN (Lux) in tumor-bearing mice, demonstrating effective tumor targeting. **(C)** Distribution of EcN (Lux) across various organs was assessed in tumor-bearing mice to determine tissue-specific colonization patterns. **(D)** Hematoxylin and eosin (H&E) staining was conducted on tumor tissues and major immune organs to observe histological changes and assess any potential tissue damage or immune responses resulting from the bacterial colonization. **(E)** As part of an *in vivo* safety test, mice with CT26 tumors received intraperitoneal injections of PBS, EcN, EcN, and EcN (PTEN). **(F)** The mice's kidney, spleen, and liver were isolated and weighed following the course of therapy. Following therapy, the blood levels of CT26 tumor-bearing mice were assessed for **(G)** alanine aminotransferase and **(H)** urea nitrogen. **(I)** Liver, **(J)** spleen, and **(K)** kidney. Adapted with permission from ref. 65 Copyrights 2024, Springer Nature.

Dai* et al.* 2024, developed an engineered strain of the probiotic *E. coli* Nissle 1917 (EcN) designed to deliver the tumor suppressor gene PTEN directly to tumor sites. PTEN is a critical regulator of the PI3K/AKT signaling pathway, and its loss or downregulation is frequently observed in various cancers, contributing to tumor progression, immune evasion, and therapy resistance. The engineered EcN was programmed to selectively colonize the tumor microenvironment (TME) and release PTEN in response to tumor-specific signals. Experimental results from *in vivo* tumor-bearing mouse models demonstrated that the engineered EcN effectively accumulated within tumor tissues. Once localized, the bacteria released functional PTEN, which led to the restoration of PTEN signaling within tumor cells. This restoration significantly inhibited tumor cell proliferation and growth. Additionally, the release of PTEN contributed to remodeling the TME by reducing immunosuppressive elements and enhancing the infiltration and activation of cytotoxic T cells (**Figure 6**). This created a more favorable immune landscape, promoting anti-tumor immune responses. Furthermore, the treatment resulted in slowed tumor progression and extended survival in mouse models compared to controls. Importantly, no significant toxicity or systemic side effects were observed, supporting the safety profile of the engineered probiotic approach 65.

In addition to cytokine and enzyme production, engineered probiotics have been designed to produce therapeutic antibodies or immune checkpoint inhibitors. These molecules are critical in regulating immune responses and have gained prominence in cancer immunotherapy. Immune checkpoint inhibitors, such as those targeting PD-1 or CTLA-4, can be used to enhance the immune system's ability to attack cancer cells. Probiotics can be engineered to express these inhibitors locally within the tumor site, where they can prevent immune suppression and promote a stronger anti-tumor response. For instance, *Lactobacillus rhamnosus* has been modified to express anti-PD-1 antibodies, which have shown to enhance immune responses in animal models of cancer 66. By strategically delivering these immune checkpoint inhibitors, engineered probiotics can improve the efficacy of existing immunotherapies, making them a promising tool for combination cancer treatments 67.

The ability to regulate the activity of engineered probiotics is another critical aspect of their therapeutic use. One of the main concerns with using live microorganisms in therapy is ensuring that they do not cause harm to the host. To mitigate this risk, several strategies have been developed to control the activity of engineered probiotics. These strategies include the incorporation of “kill switches,” which can be activated to eliminate the engineered microorganisms if necessary. For example, *Lactobacillus* strains can be genetically modified to include an inducible suicide gene that can be triggered by an external stimulus, such as the presence of a specific drug or environmental factor 68. This safety mechanism ensures that the probiotic does not persist in the host after its therapeutic function is completed, reducing the risk of unintended consequences.

Furthermore, the engineering of probiotics to produce biofilms has gained attention as a strategy to enhance their persistence in the host. Biofilms are dense clusters of microorganisms encased in a self-produced matrix, and they can improve the stability and persistence of probiotics in the gastrointestinal tract. The development of biofilms also helps probiotics withstand challenging environments like the acidic conditions of the stomach and the presence of bile salts in the intestines. Engineered probiotics that form biofilms may be more effective in reaching tumors located in the gut or other organs, providing an additional avenue for targeted cancer therapies 69.

The use of probiotics as delivery vehicles for genetic material has also shown promise in cancer therapy. By engineering probiotics to carry plasmids or RNA molecules that encode therapeutic proteins, it is possible to deliver these molecules directly to the tumor site. For example, *Bifidobacterium* strains have been genetically modified to carry plasmids that encode for tumor necrosis factor-alpha (TNF-α), a cytokine with potent anti-tumor activity. These probiotics, once administered to the host, can target the tumor site and release TNF-α, promoting the destruction of cancer cells. This approach highlights the potential of engineered probiotics not only to modulate immune responses but also to deliver genetic material that can directly interfere with tumor growth 70.

**Figure 7:**
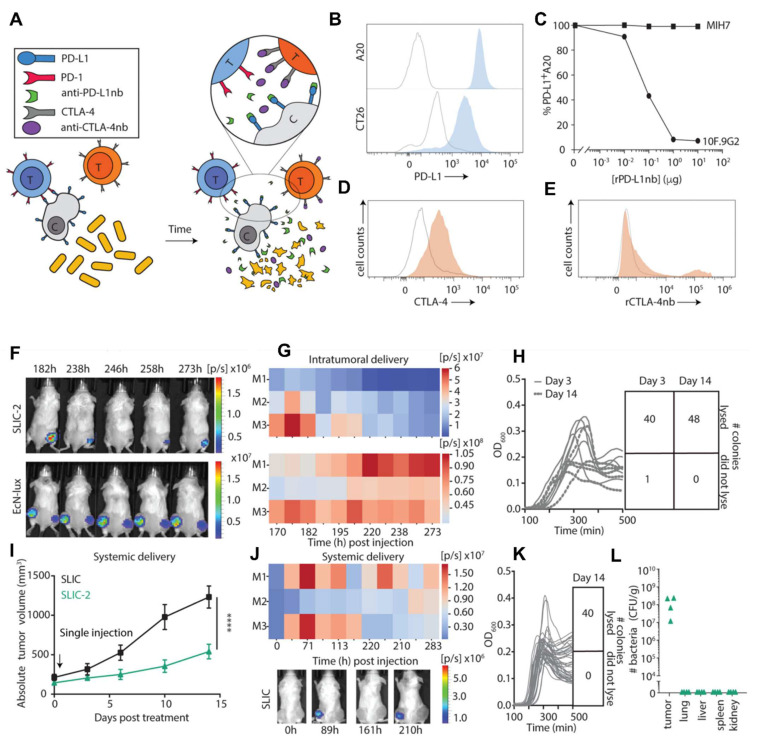
**(A)** Diagram illustrating the mechanism by which engineered bacterial strains enable controlled intratumoral release of continuously produced nanobodies targeting PD-L1 and CTLA-4. **(B)** Flow cytometry analysis showing PD-L1 surface expression on A20 and CT26 cells (gray: unstained control; blue: PD-L1-stained), with the y-axis of histograms indicating cell counts normalized to the mode. **(C)** Binding affinity curves of recombinant PD-L1 nanobody (rPD-L1nb) to the 10F.9G2 and MIH7 PD-L1 epitopes on A20 cells **(D)** intracellular CTLA-4 expression in CD3+ splenocytes under unstimulated (gray) and PMA/Ionomycin-stimulated (orange) conditions, with cell count normalized to mode on the y-axis; **(E)** binding of recombinant CTLA-4 nanobody (rCTLA-4nb) to extracellular CTLA-4 (gray: secondary anti-HIS antibody only; orange: rCTLA-4nb), also gated on CD3+ cells and normalized to mode. **(F)** Representative *in vivo* imaging system (IVIS) images of mice administered a single dose of either non-lysing EcN-lux or SLIC-2. **(G)** Heatmaps displaying the total bioluminescent signal (photons/sec) over time, corresponding to panel F. **(H)** Plate reader assay showing lysis oscillation patterns in colonies extracted from tumors on days 3 and 14 post-treatment, along with a grid indicating the frequency of successful lysis events. **(I)** average tumor growth trajectories (n = 9-11 tumors per group) analyzed via two-way ANOVA with Bonferroni post hoc test **(J)** Representative IVIS images and corresponding heatmaps showing luminescent signal over time in SLIC-2-treated mice; **(K)** Plate reader results from colonies harvested on day 14, illustrating lysis oscillations and a grid of successful lysis events. **(L)** Distribution of bacterial populations in tumors and various organs (liver, lungs, spleen, kidneys), expressed as colony-forming units per gram of tissue (CFU/g). Adapted with permission from ref. 71 Copyrights 2020, Science.

A study conducted by Gurbatri* et al.* 2020 and they engineered the probiotic strain *E. coli* Nissle 1917 to produce nanobodies targeting PD-L1 and CTLA-4, two critical immune checkpoint proteins. These bacteria were designed with a synchronized lysis circuit (SLIC) that enables them to self-destruct at a specific population density, releasing the therapeutic nanobodies directly into the tumor microenvironment. In mouse models of lymphoma and colorectal cancer, a single intratumoral injection of these engineered probiotics led to sustained local production of checkpoint inhibitors, resulting in significant tumor regression. The treatment not only affected the primary tumor but also induced systemic antitumor immunity, evidenced by increased activation of T cells and the regression of untreated tumors, demonstrating an abscopal effect. Furthermore, the modular nature of this probiotic platform allowed for the co-expression of granulocyte-macrophage colony-stimulating factor (GM-CSF), an immunostimulatory cytokine. This combination therapy enhanced the antitumor response, particularly in tumors that are less responsive to immunotherapy alone. This approach addresses the limitations of systemic checkpoint inhibitor therapies, which can cause widespread immune-related side effects. By localizing the delivery of immunotherapeutics, the engineered probiotics minimize systemic toxicity while maximizing therapeutic efficacy. The study demonstrates the potential of synthetic biology in developing targeted cancer treatments that harness the body's immune system with greater precision and fewer side effects **(Figure 7)** 71.

In recent years, there has been increasing interest in the use of synthetic biology tools, such as CRISPR/Cas9, to facilitate the engineering of probiotics for cancer therapy. These tools allow for precise modifications of the probiotic genome, enabling the creation of highly tailored strains with specific functions. For example, CRISPR/Cas9 has been used to engineer *Lactobacillus* strains to produce antimicrobial peptides that can target and kill tumor cells 72. The precision of these genetic modifications ensures that the engineered probiotics are capable of performing their desired therapeutic functions without causing harm to healthy tissues.

Despite the promising potential of engineered probiotics in cancer therapy, several challenges remain in their clinical application. One of the main hurdles is the variability of the human microbiome, which may affect the efficacy of probiotic therapies. The microbiome is highly individual, and the presence of specific microbial species can influence how probiotics interact with the immune system and the TME 73. Personalized approaches that consider the individual microbiome composition may be necessary to optimize the therapeutic outcomes of probiotic-based treatments. In addition, regulatory issues related to the safety and effectiveness of genetically modified organisms (GMOs) continue to pose major obstacles for bringing engineered probiotics into clinical use. Establishing strict safety standards and comprehensive regulatory guidelines will be crucial to guarantee that engineered probiotics can be used safely and successfully in cancer treatments 74.

Engineering strategies for living probiotics involve genetic and physicochemical modifications to enhance their therapeutic potential. These approaches include designing genetic circuits, metabolic pathway optimization, and surface modifications to improve targeting, stability, and immune modulation. Such strategies enable probiotics to serve as precise and effective tools for applications ranging from gut health to cancer therapy.

### 4.1. Synthetic biology tools for probiotic design

The application of synthetic biology to probiotic design has revolutionized the ability to engineer microorganisms with enhanced therapeutic potential, especially for cancer therapy. Synthetic biology combines molecular biology, bioengineering, and genetic modification to construct novel biological systems that do not exist in nature. This approach has expanded the scope of probiotics, traditionally used for improving gut health, into more specialized therapeutic agents capable of modulating immune responses, delivering drugs, and even targeting cancer cells. The ability to engineer probiotics using synthetic biology tools has made them promising candidates for improving existing treatments and introducing novel strategies in cancer immunotherapy 75.

One of the most powerful tools in synthetic biology is CRISPR/Cas9, a gene-editing technology that enables precise, targeted modifications in the genomes of living organisms. This system has been widely used in engineering probiotics to express desired genes that enhance their therapeutic capabilities 76. A guide RNA sequence directs the Cas9 protein to the desired location in the genome, enabling precise modifications. This has allowed the engineering of probiotics such as *Lactobacillus* strains, which have been modified to produce therapeutic proteins like tumor necrosis factor-alpha (TNF-α), a cytokine that can enhance immune responses and promote tumor cell death 77.

For example, synthetic biology has been used to modify *Lactobacillus* species to express high levels of antimicrobial peptides (AMPs), which are molecules that have anti-cancer activity. The introduction of AMP genes into *Lactobacillus* not only enhances its ability to target cancer cells but also increases its persistence in the host by allowing the bacteria to survive in the harsh conditions of the gastrointestinal tract. One study utilized CRISPR to engineer *Lactobacillus rhamnosus* to produce an AMP, which in turn, exhibited significant anti-tumor effects in murine models by inducing tumor cell apoptosis 78. This application of synthetic biology allows the generation of probiotics that are not only able to modulate the microbiome but also actively participate in cancer cell eradication. Beyond CRISPR/Cas9, other synthetic biology tools like RNA-guided gene silencing systems have also been leveraged to create probiotics that can modulate gene expression within the host or tumor microenvironment. For example, RNA interference (RNAi) technology can be employed to engineer probiotics capable of silencing specific genes that contribute to immune suppression in cancer 79. These probiotics can be designed to express small RNA molecules that interfere with the expression of immune checkpoint proteins, such as PD-L1, within the tumor microenvironment. This approach is particularly useful for enhancing the efficacy of immune checkpoint inhibitors, which are now widely used in cancer immunotherapy. By incorporating RNAi into engineered probiotics, researchers have been able to create a dynamic system in which probiotics actively promote immune responses by silencing immune-suppressive pathways 80.

**Figure 8:**
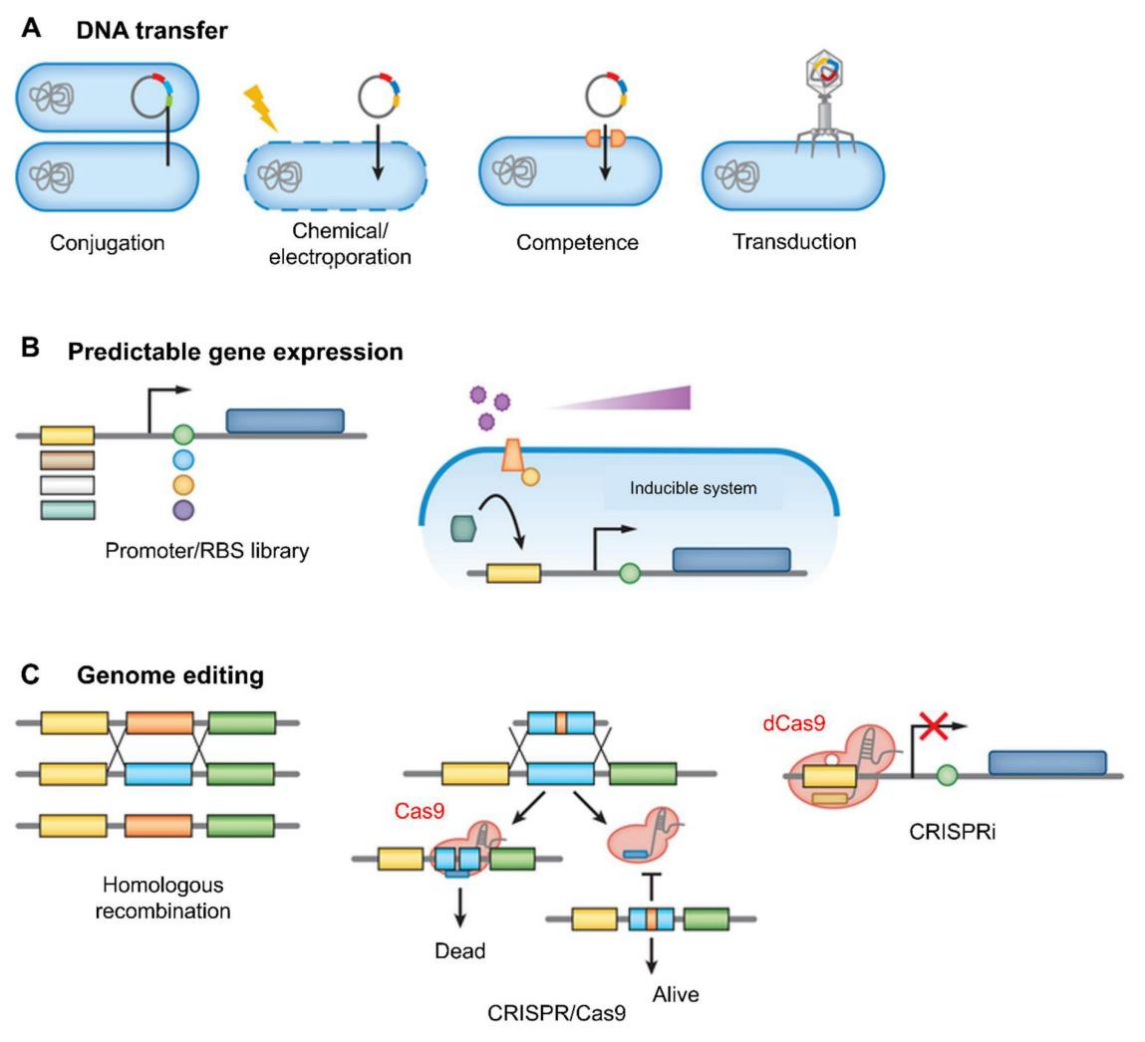
The genetic engineering of host-associated microbes relies on effective methods for modifying their genetic material. **(A)** DNA can be introduced into these microbes through various techniques, including conjugation, electroporation or chemical treatments that disrupt the cell membrane, induction of natural competence, or phage-mediated transduction. **(B)** Control over gene or pathway expression is achieved by constructing libraries of mutant promoters and/or ribosome binding sites (RBS), or by implementing inducible systems where gene expression is regulated by the concentration of an externally supplied molecule. **(C)** Traditionally, genome editing has been carried out using homologous recombination facilitated by selectable markers. However, recent advances in CRISPR-based technologies have greatly expanded the capabilities for genome-scale engineering. One approach, using the CRISPR-Cas9 system, leverages Cas9 to introduce double-strand breaks in unmodified DNA, serving as a form of negative selection. Another technique, CRISPR interference (CRISPRi), employs a catalytically inactive variant of Cas9 known as dCas9. This modified enzyme binds to specific DNA sequences without cutting them, thereby regulating gene expression by obstructing transcription. Adapted with permission from ref. 81 Copyrights 2018, Annual Review.

According to Bober* et al*. 2018, the human microbiota plays a crucial role in maintaining health, with disruptions linked to a wide range of diseases. Engineered strains of lactic acid bacteria, *Bifidobacteria*, and *Bacteroides* are now being used to sense disease biomarkers, deliver therapeutic molecules, and even reshape the gut environment. One major advancement highlighted is the development of genetic tools and synthetic circuits. These include CRISPR-based systems and modular genetic parts that allow bacteria to detect specific cues such as pH changes, metabolic byproducts, or inflammatory signals and respond accordingly **(Figure 8)**. These engineered microbes can produce anti-inflammatory cytokines like IL-10, secrete antimicrobial peptides, or deliver enzymes that neutralize toxins and restore gut balance. For instance, *Lactococcus lactis* has been engineered to secrete IL-10 to treat colitis, while *E. coli* Nissle has been modified to combat *Vibrio cholerae* and inhibit pathogenic biofilms 81.

Another key tool in synthetic biology for probiotic design is the recombinase-based genetic circuits. These circuits, often referred to as genetic switches, are used to control gene expression in response to specific environmental signals. This approach ensures that the probiotic's therapeutic function is activated only in the presence of certain triggers, which is especially important for minimizing off-target effects 82. For example, probiotics can be designed to release cytokines or other active compounds exclusively when triggered by certain conditions within the tumor microenvironment, like low oxygen levels or acidic pH. This targeted approach helps to localize therapeutic effects to the tumor site, minimizing the risk of unwanted systemic side effects 83. An example of such a strategy is the engineering of *Lactococcus lactis*, a probiotic bacterium, to express interleukin-12 (IL-12) when exposed to acidic conditions similar to those found in the TME 84. This system allows for the controlled release of the cytokine, enhancing anti-tumor immunity without causing unintended activation of immune responses in healthy tissues.

The use of biosensors is another critical aspect of synthetic biology that facilitates the engineering of probiotics. Biosensors are molecular devices that can detect specific metabolites or environmental factors and, in response, activate gene expression 85. In cancer immunotherapy, biosensors can be engineered into probiotics to detect tumor-specific metabolites, such as lactate, a byproduct of the anaerobic metabolism commonly found in the tumor microenvironment. Once these biosensors detect the presence of such metabolites, they can trigger the expression of therapeutic genes within the probiotic. This type of engineering ensures that the probiotics are only activated when they encounter the tumor, thereby enhancing their specificity and reducing the likelihood of off-target effects 86. An example of this is the use of *E. coli* strains engineered with biosensors for detecting the presence of metabolites like nitrate, which are elevated in tumors compared to normal tissues. Upon detection, these probiotics produce tumor-targeted therapeutic molecules such as cytokines or cytotoxic agents 87.

The combination of these synthetic biology tools holds immense promise for the development of engineered probiotics as effective therapeutic agents. However, several challenges remain in the translation of these technologies into clinical applications. These challenges include concerns regarding the safety and regulation of genetically modified organisms (GMOs) and the variability of the human microbiome, which can affect the effectiveness of engineered probiotics. Addressing these issues through rigorous safety testing and personalized approaches to microbiome engineering will be key to the successful application of synthetic biology in probiotic-based cancer therapies 88. Synthetic biology tools such as CRISPR/Cas9, biosensors, gene synthesis, and pathway construction have greatly expanded the potential of engineered probiotics for cancer immunotherapy. These tools enable the precise modification of probiotics to produce therapeutic agents, respond to environmental cues, and interact with the immune system in novel ways. As research continues to evolve, engineered probiotics will play an increasingly important role in enhancing the efficacy of cancer treatments and improving patient outcomes 89.

### 4.2. Bacteria-based living probes

Bacteria-based living probes represent a rapidly evolving class of biosensors that harness the innate biological properties of microbes for real-time detection, imaging, and therapeutic monitoring 90. Unlike conventional chemical or molecular probes, which are limited by diffusion, degradation, or lack of specificity, living bacterial systems offer the unique advantage of self-replication, environmental responsiveness, and the ability to be genetically engineered for complex sensing tasks. These features make bacterial probes particularly attractive for biomedical, environmental, and synthetic biology applications. Bacterial systems can be engineered to detect a wide range of analytes or conditions, including specific metabolites, toxins, pollutants, and changes in pH, oxygen levels, or redox states 91. The use of bacteria as living probes is built upon the integration of synthetic genetic circuits, often composed of inducible promoters, sensor modules, reporter genes, and feedback systems. Upon encountering the target analyte or environmental cue, the engineered bacteria respond with a measurable output, typically in the form of fluorescence, bioluminescence, color change, or even therapeutic payload release 92.

One of the most established applications of bacteria-based probes is in bioluminescent and fluorescent imaging. Engineered strains of *E. coli*, *Salmonella*, or *Pseudomonas* have been designed to express luciferase or green fluorescent protein (GFP) under the control of promoters responsive to environmental signals. For example, *E. coli* strains harboring arsenic-responsive promoters fused with GFP can detect arsenic contamination in water at very low concentrations, with the fluorescence intensity correlating with toxicity levels. Similarly, *Salmonella typhimurium* has been engineered to colonize tumor tissues and emit bioluminescence, enabling the *in vivo* imaging of tumor localization, progression, and response to therapy 93.

In tumor targeting and diagnosis, bacterial probes exploit the natural tendency of certain anaerobic or facultative anaerobic bacteria (e.g., *Clostridium*, *Bifidobacterium*, *Salmonella*) to accumulate in hypoxic tumor microenvironments 94. These bacteria can be genetically modified to express reporter genes only under tumor-specific conditions, enabling the detection of solid tumors that are otherwise difficult to image using traditional methods. In recent years, such approaches have been extended to design dual-functional bacterial probes capable of both diagnosis and drug delivery paving the way for theranostic applications 95. Beyond imaging, bacteria-based biosensors have been widely applied in environmental monitoring. Engineered microbes have been developed to detect heavy metals (like mercury, cadmium, lead), organic pollutants (such as toluene and benzene), and endocrine-disrupting chemicals. These systems often rely on metal- or xenobiotic-responsive genetic elements that drive the expression of easily quantifiable reporters. Because bacterial cells can survive in complex environments and can be deployed at low cost, they offer a scalable and sensitive alternative to traditional analytical techniques such as chromatography or mass spectrometry 96.

In clinical diagnostics, researchers have engineered probiotic strains such as *E. coli* Nissle 1917 or *Lactobacillus* spp. to detect disease biomarkers in the gut. These bacteria can be programmed to produce visible outputs such as color changes in stool samples or to secrete detectable metabolites when encountering specific disease conditions like inflammation, gastrointestinal bleeding, or colorectal cancer. Some synthetic systems use quorum-sensing circuits to enable coordinated responses among bacterial populations, amplifying detection signals and improving robustness 97. A particularly innovative direction involves programmed cell death and signal release. Some bacteria-based probes are designed to self-lyse upon detecting target analytes, releasing their contents including diagnostic molecules or therapeutic compounds into the environment. This strategy is especially useful when the signal must be spatially confined or when single-use detection is required. Such self-destruction modules are controlled by tightly regulated promoters or toxin-antitoxin systems, ensuring safety and specificity 98.

Liu and Chang (2022) present a comprehensive tutorial review that advances the field of live-cell identification by systematically discussing fluorescent probe strategies that go beyond traditional antibody-based surface marker characterization. They categorize probe designs into six major approaches protein-oriented (POLD), carbohydrate-oriented (COLD), DNA-oriented (DOLD), gating-oriented (GOLD), metabolism-oriented (MOLD), and lipid-oriented (LOLD) each exploiting unique biochemical features for cell-type discrimination. They delve into the fundamental mechanisms enabling probe retention in cells, including targeted biomolecular interactions, transporter-mediated uptake, and metabolic incorporation. Through a detailed examination of probe design principles and their deployment in differentiating diverse cell populations, Liu and Chang also highlight the advantages of fluorescent strategies such as broader biomarker coverage and intracellular accessibility and address key challenges like specificity, delivery, and signal regulation. By offering clear conceptual frameworks and illustrative examples, this work serves as a valuable guide for developing next-generation cell-type-specific fluorescent probes and facilitates deeper insights into complex biological systems 99.

Huang* et al*. 2021 offer an authoritative review of the latest advancements in fluorescent probes for bacterial detection and imaging, underscoring the urgent need for rapid, noninvasive, and highly specific tools in healthcare, environmental science, and food safety. The authors systematically classify probe design strategies based on bacterial features, including cell wall composition, surface charge, hydrophobicity, endogenous enzymatic activity, and unique outer membrane components. They comprehensively cover various probe formats including small-molecule fluorescent dyes, nanoprobes, and metal-ion-based sensors highlighting their respective mechanisms of action and application domains. Notably, they emphasize intelligent designs that exploit bacterial enzymes (e.g., nitroreductases), metabolic labeling, and peptidoglycan-targeting to achieve high selectivity between Gram-positive and Gram-negative species. They also discuss the integration of aggregation-induced emission (AIE) luminogens, turn-on fluorescence systems, and wash-free imaging methods, reflecting the field's shift towards operational simplicity and *in situ* applicability. In closing, Huang* et al*. articulate future challenges such as improving sensitivity in complex biological matrices, expanding the scope of bacterial species detected, and advancing translational use in clinical diagnostics while outlining promising directions for the rational design of next-generation fluorescent bacterial probes 100.

Wang *et al*. (2020) report a groundbreaking strategy for non-invasive, deep-tissue imaging of gut microbiota in live mice by combining peptidoglycan metabolic labeling with a second near-infrared (NIR-II) fluorescent dye. The researchers first administered d-propargylglycine, a D-amino acid analog, to mice via gavage, which became incorporated into the bacterial peptidoglycan layer. Following this metabolic step, the fluorophore IR-FGN, equipped with an azide group, was covalently attached through a copper-free click reaction, yielding stable, brightly NIR-II-labeled bacteria that retain viability and physiological function. Upon transplanting these labeled bacteria into recipient animals, the authors successfully visualized their localization and dynamics within the gastrointestinal tract with high spatial resolution and deep penetration, facilitated by NIR-II light's superior tissue transparency. This represents the first instance of real-time, *in vivo* NIR-II imaging of gut microbial populations, offering an invaluable tool for exploring microbiota behavior under physiological and pathological conditions and paving the way for refined studies of gut “dark matter” 101.

**Figure 9:**
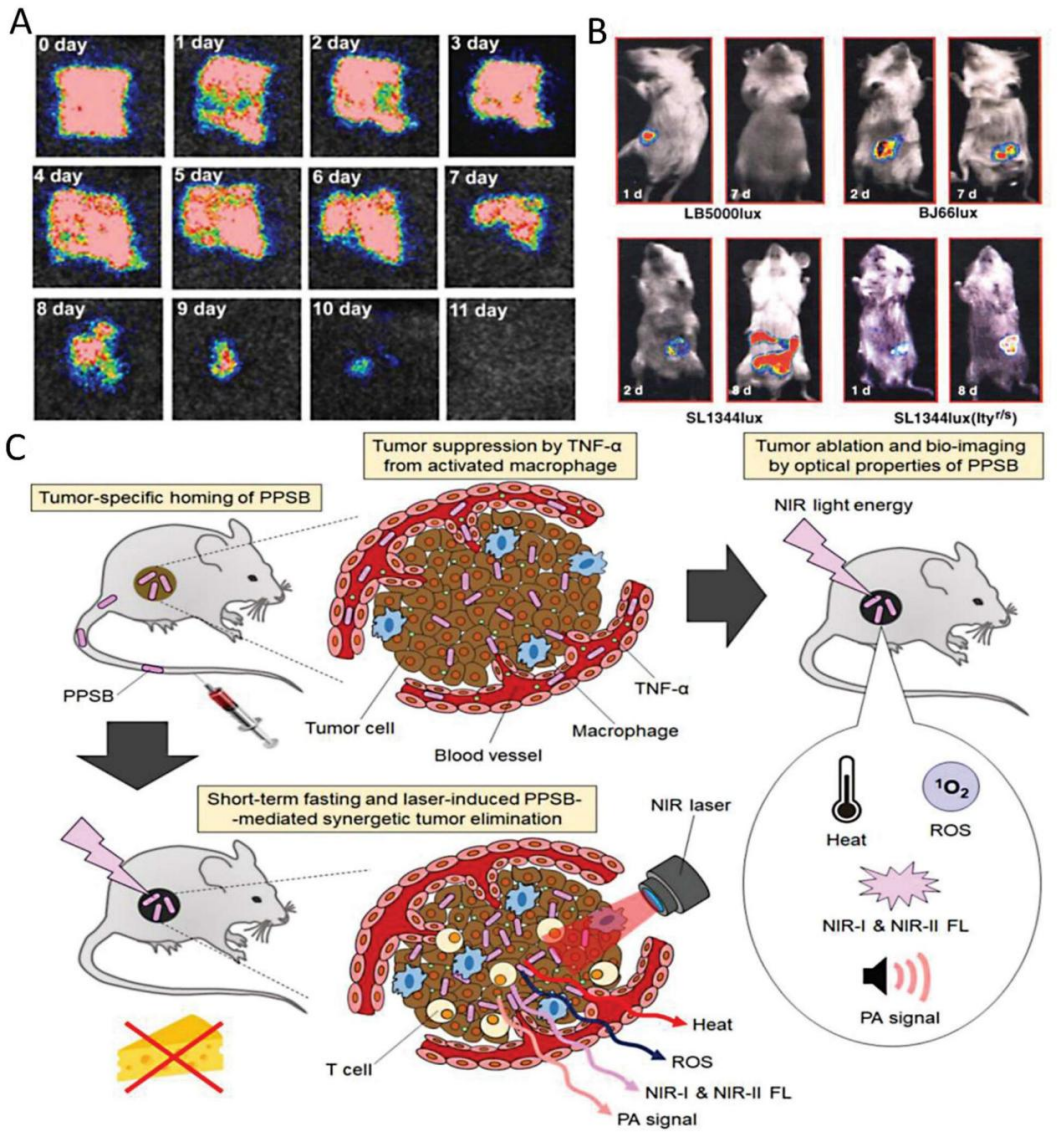
Delivery of alive probes based on bacteria for optical imaging. **(A-B)** Imaging using bioluminescence. **(C)** NIR imaging between 650 and 900 nm. Reused under Creative Commons Attribution License from ref. 102.

Jiang *et al*. (2024) present a comprehensive review on the design and utilization of bacteria-based living probes, highlighting their preparation and broad biomedical applications. Bacteria, with their inherent motility, genetic tractability, and site-specific colonization, are engineered through four primary strategies: biological engineering (e.g., reporter gene expression), chemical modification (e.g., surface dye conjugation), intracellular loading (e.g., nanoparticle cargo), and optical manipulation (e.g., light-activated labeling). The review elaborates on their implementation in multimodal imaging platforms such as fluorescence, near-infrared, ultrasound, photoacoustic, MRI, and PET demonstrating their versatility for tracing infections, tumors, and gut microbiota *in vivo*. Jiang et al. underscore notable examples, including bioluminescent *E. coli* for tumor localization, photoacoustic probes derived from melanin-producing strains, bacterioferritin-expressing bacteria for MRI contrast, and PET imaging via metabolically labeled microbe **(Figure 9)**. They further discuss successful applications in diagnosing bacterial infections, cancer imaging, and intestinal disease, while also addressing challenges related to biosafety, signal stability, and translational viability. They conclude by emphasizing the potential of engineered bacterial probes as intelligent, multifunctional platforms for next-generation diagnostics and theranostics 102.

Liu* et al*. 2020 present a thorough analysis of recent strategies in designing fluorescent probes for cell plasma membrane (PM) imaging, emphasizing both functional innovation and application versatility. They first address targeting mechanisms, detailing how amphiphilic motifs comprising charged polar heads and hydrophobic alkyl chains enable efficient and selective PM intercalation without cellular penetration. Building on this foundation, the authors classify PM probes into simple labeling dyes for morphological studies and responsive probes capable of sensing membrane-associated biochemical and biophysical changes, such as pH shifts, metal-ion fluxes, reactive species, and mechanical stress many achieving ratiometric or fluorogenic performance. Recognizing the demands of modern microscopy, they highlight the development of PM probes compatible with super-resolution techniques (e.g., STED, PALM/STORM), featuring properties like switchable emission and photostability that surpass conventional dyes. This study concludes by outlining key technical challenges, including enhancing probe specificity, reducing cytotoxicity, and improving real-time dynamic imaging, and proposes future directions such as multifunctional labeling and integration with advanced imaging platforms. Overall, this work offers valuable insights into the evolving landscape of PM-targeted fluorescent probes for cutting-edge bioimaging 103.

## 5. Therapeutic Modalities Delivered by Engineered Probiotics

Engineered probiotics are a promising avenue in the development of novel therapeutic modalities for various diseases, particularly in cancer therapy. These living microorganisms can be genetically modified to deliver a range of therapeutic agents directly to target sites in the body, where they can exert localized effects. The therapeutic modalities delivered by engineered probiotics include immunotherapies, cytokines, tumor-targeted therapeutics, and gene therapies. By using probiotics as delivery vehicles, researchers aim to enhance treatment specificity, minimize side effects, and improve therapeutic outcomes 104.

The concept of using engineered probiotics for therapy hinges on their ability to safely and effectively deliver bioactive molecules directly to affected areas, such as tumors, while maintaining minimal systemic exposure. This allows for a more focused therapeutic action with reduced off-target effects, which is particularly crucial in cancer treatment 105. A particularly exciting use of engineered probiotics lies in their capacity to influence the immune system, especially for cancer immunotherapy. Cancer cells often evade immune surveillance by employing various mechanisms, such as immune checkpoint inhibition or the secretion of immunosuppressive factors. Engineered probiotics can be designed to deliver cytokines, immune-stimulatory molecules, or even genetic modifications that enhance the body's natural immune response to tumors 106.

Cytokines are molecules that transmit signals to control immune activity and are crucial in cancer immunotherapy. Engineered probiotics can be used as vehicles to deliver cytokines to the tumor microenvironment (TME), where they can help boost the immune system's ability to recognize and destroy cancer cells 107. One of the most studied cytokines for this purpose is IL-12, a potent immunostimulatory cytokine known for its ability to activate T cells and natural killer (NK) cells and promote anti-tumor immunity. Research by Gorski* et al*. 2015 showed that genetically engineered *Lactobacillus rhamnosus* could express IL-12 in response to acidic conditions, which is a common feature of the TME. This approach ensured that the probiotic only delivered IL-12 at the tumor site, thus minimizing the potential for systemic inflammatory responses and enhancing the localized immune response 108.

Similarly, *Lactococcus lactis* has been engineered to deliver interferons (IFNs), another class of cytokines with potent anti-tumor properties. IFNs boost the immune system by activating T cells and natural killer (NK) cells and by raising the levels of major histocompatibility complex (MHC) molecules on tumor cell surfaces. By using engineered probiotics as cytokine delivery vectors, it is possible to target the tumor microenvironment specifically, where the immune-modulating effects of IFNs can be most beneficial without affecting healthy tissues 109. Another novel strategy is the use of engineered probiotics to deliver immune checkpoint inhibitors (ICIs) directly to the tumor site. ICIs, such as anti-PD-1 or anti-CTLA-4 antibodies, are designed to block inhibitory signals that cancer cells use to evade immune detection. The delivery of ICIs via engineered probiotics offers a unique opportunity to localize their action to the TME, thus reducing the risk of systemic immune activation and the associated side effects, such as autoimmune reactions. *E. coli* and *Lactobacillus* species have been engineered to produce checkpoint inhibitors like anti-PD-1 antibodies in response to specific inducers. This targeted delivery system allows for sustained and localized immune modulation, potentially enhancing the effectiveness of immunotherapy 110.

The ability of engineered probiotics to deliver tumor-targeted therapeutics is another key therapeutic modality with significant potential. Tumor-targeted therapies are designed to selectively target cancer cells while sparing healthy tissues 111. This specificity is crucial for minimizing off-target effects and enhancing the therapeutic index of cancer treatments. Engineered probiotics can be used to produce and deliver therapeutic proteins directly to tumors. For instance, cancer-specific enzymes or toxic proteins can be expressed by probiotics, where they can then be activated or induced upon reaching the tumor. One such approach is the use of probiotics to deliver prodrugs. Prodrugs are inactive compounds that are converted into their active form by enzymes 112. By engineering probiotics to produce the necessary enzyme, researchers can ensure that the prodrug is only activated in the tumor microenvironment. This targeted drug delivery system has been investigated using *Lactobacillus* species to express the enzyme cytosine deaminase, which converts the prodrug 5-fluorocytosine into the toxic 5-fluorouracil 113. This approach offers the potential for selective tumor targeting, as the drug is activated only in the vicinity of the tumor, reducing systemic toxicity.

Gene therapy is another promising modality delivered by engineered probiotics. In gene therapy, genetic material is introduced into the patient's cells to correct defective genes or to produce therapeutic proteins. Engineered probiotics can be used as vehicles for gene delivery, enabling the localized release of therapeutic genes to treat cancer. For example, probiotics can be engineered to carry and deliver genes encoding tumor-suppressor proteins, such as p53, which is often mutated or inactive in many cancers. By delivering functional copies of these genes directly to tumor cells, engineered probiotics can help restore normal cell function and induce tumor cell death 114. Additionally, engineered probiotics can be utilized to deliver RNA-based therapeutics, such as small interfering RNAs (siRNAs) or messenger RNAs (mRNAs), to silence oncogenes or promote the expression of tumor-suppressor genes. RNA-based therapies offer a highly targeted approach to cancer treatment by interfering with the expression of specific genes involved in tumor progression. Research by Zhao* et al*. 2018 demonstrated the potential of engineered *E. coli* to deliver siRNA molecules that silence key oncogenes in prostate cancer cells. This approach provides a platform for the targeted delivery of gene therapies directly to the tumor site, where they can effectively modify gene expression in cancer cells and restore normal cell function 115. In addition to protein-based and gene-based therapies, engineered probiotics can also deliver microbial enzymes that directly target tumor cells. These enzymes can break down the extracellular matrix (ECM) or degrade other components of the tumor stroma, thus facilitating the infiltration of immune cells into the tumor 116.

**Figure 10:**
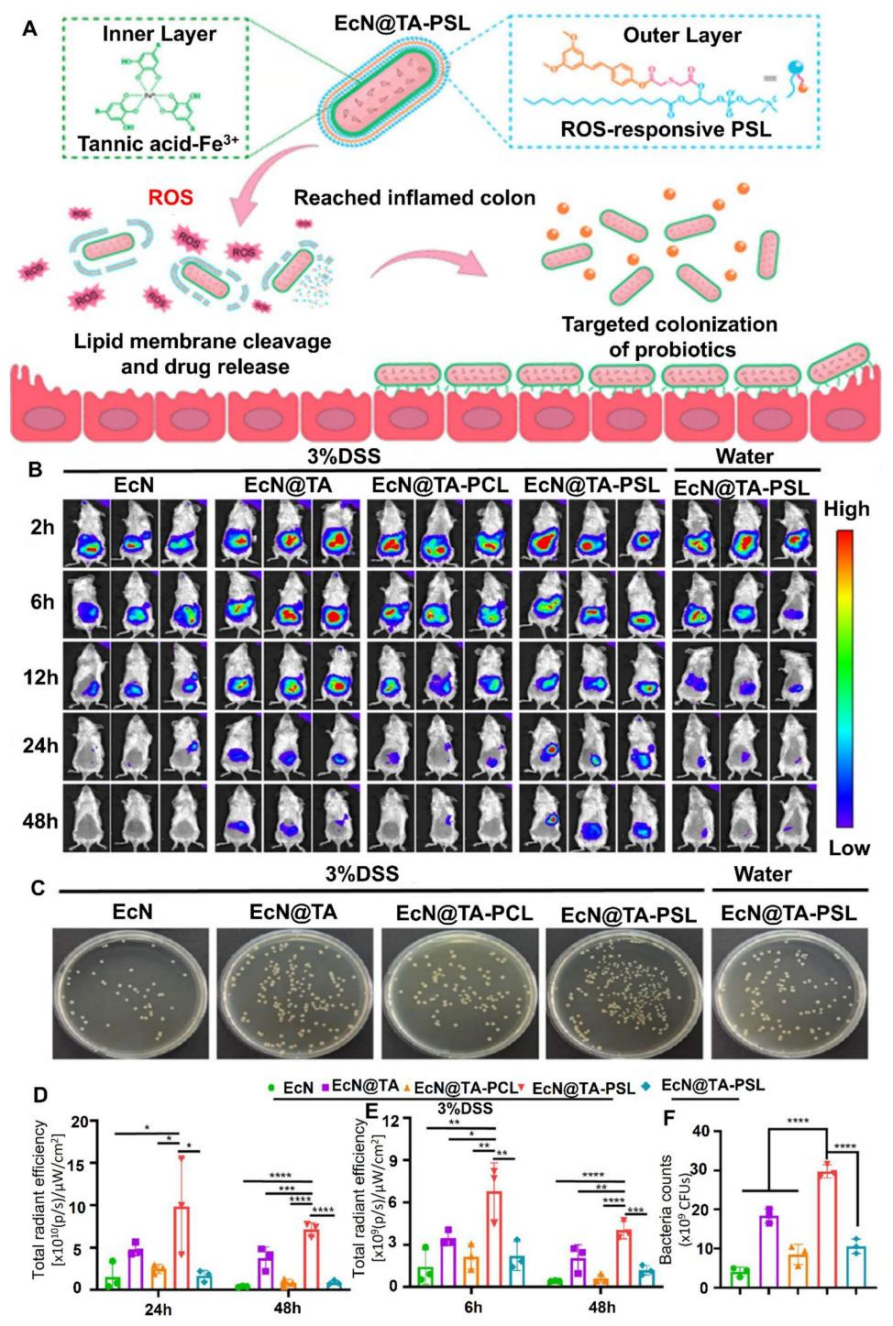
** (A)** Illustration showing the synthesis of ROS-responsive PSL and the fabrication steps of EcN@TA-PSL, along with a schematic of EcN colonizing the lesion site and releasing pterostilbene in a targeted manner. **(B)** *In vivo* imaging (IVIS) of IBD mice at 2, 6, 12, 24, and 48 hours following oral administration of native EcN, EcN@TA, EcN@TA-PCL, or EcN@TA-PSL. Healthy mice treated with EcN@TA-PSL served as controls. Each mouse received an oral dose of 1 × 10^8 CFU of live, DiR-labeled EcN (ampicillin-resistant), either uncoated or coated. **(C)** Representative agar plate images showing EcN recovered from colonic homogenates 48 hours after treatment. **(D)** Quantification of total radiant efficiency in the abdominal region at 24 and 48 hours. **(E)** Measurement of radiant efficiency in isolated colonic tissue at 6 and 48 hours. **(F)** Enumeration of EcN colonies obtained from colon homogenates of different groups at 48 hours. Adapted with permission from ref. 117 Copyrights 2025, American Chemical Society.

The study by Linzhou *et al.* 2025, presents an innovative therapeutic strategy for ulcerative colitis by developing probiotic-drug conjugates that combine site-specific colonization with controlled drug release. Using *Escherichia coli* Nissle 1917 as the probiotic carrier, the researchers designed a dual-layer protective system in which an inner tannic acid-ferric complex ensures bacterial stability and adhesion, while an outer pterostilbene conjugate layer remains stable during gastrointestinal transit but responds to the elevated reactive oxygen species (ROS) present in inflamed colonic tissue. Upon reaching the disease site, the ROS-sensitive layer degrades, releasing the anti-inflammatory compound pterostilbene while simultaneously exposing the adhesive probiotic for colonization. In mouse models of ulcerative colitis, this system not only reduced inflammatory markers and tissue damage but also improved microbiota balance and decreased disease recurrence. The findings highlight the potential of synchronizing probiotic activity with on-demand drug delivery as a powerful approach to treat ulcerative colitis and related complications, offering both therapeutic and preventive benefits **(Figure 10)** 117.

**Figure 11:**
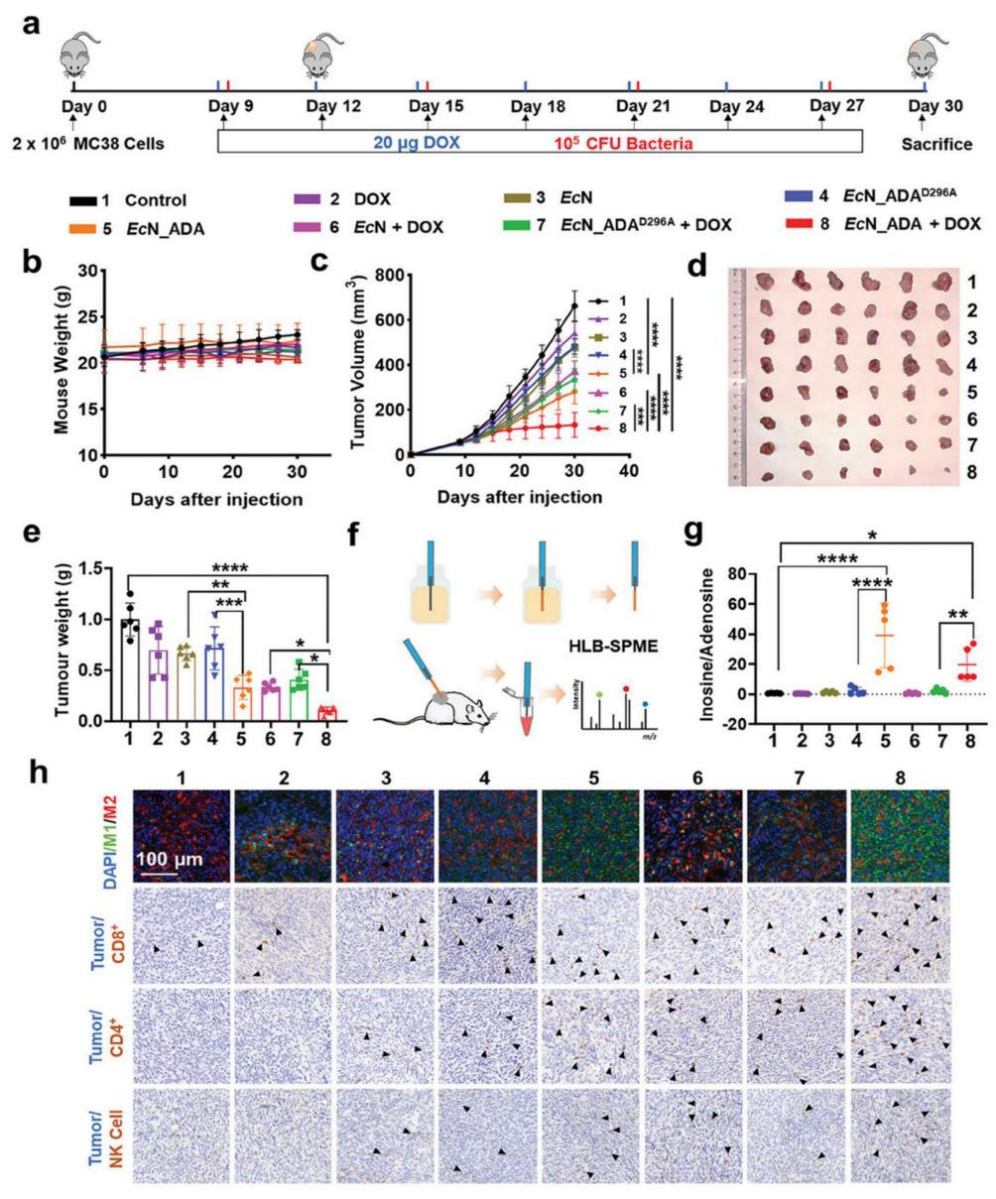
Combined Treatment with Engineered Probiotics and Doxorubicin Suppresses Tumor Progression in a Mouse Subcutaneous MC38 Tumor Model **(A)** Illustration of the therapeutic strategy integrating engineered probiotics with doxorubicin in mice bearing subcutaneous MC38 tumors. **(B)** Body weight and **(C)** tumor volume measurements were recorded over time following different treatments (n = 6). **(D)** Photographic images of excised tumors and **(E)** corresponding tumor weights from each treatment group are shown (n = 6). Data are presented as mean ± standard deviation from three mice per group. **(F)** Diagram outlining the workflow for quantifying adenosine and inosine levels in tumor tissues using solid-phase microextraction (SPME). **(G)** The ratio of inosine to adenosine was measured across experimental groups. **(H)** Immunohistochemistry of tumor tissues displayed immune cell infiltration, including M1 and M2 macrophages (green), as well as CD8⁺ T cells, CD4⁺ T cells, and NK cells. Black arrowheads indicate the presence of infiltrating immune cells. Reused under Creative Commons Attribution License from ref. 118.

Wang* et al*. 2025 developed a strain of *E. coli* Nissle 1917 (EcN) engineered to express adenosine deaminase (ADA) on its surface under hypoxic conditions, which are characteristic of tumor environments. This modification allows bacteria to transform immunosuppressive adenosine into inosine within the TME. Adenosine accumulation in tumors is known to inhibit immune cell activity via cAMP signaling pathways, thereby facilitating tumor immune evasion. *In vivo* experiments demonstrated that administration of the engineered EcN strain led to a significant reduction in extracellular adenosine levels, resulting in enhanced infiltration and activation of immune cells, including a shift from M2-like (immunosuppressive) to M1-like (pro-inflammatory) macrophages. Furthermore, when combined with low-dose doxorubicin chemotherapy, the treatment exhibited synergistic effects, leading to substantial tumor regression in both subcutaneous and orthotopic mouse models of colorectal cancer. They highlight the potential of using engineered probiotics as a novel strategy to remodel the immunosuppressive TME by targeting metabolic pathways, thereby enhancing the efficacy of existing cancer immunotherapies **(Figure 11)**. The approach offers a promising avenue for developing more effective and targeted cancer treatments with potentially fewer side effects 118.

Another approach involves the use of bacterial enzymes that activate chemotherapeutic agents by converting them into their active forms. For example, the enzyme beta-glucuronidase can be engineered into probiotics to activate certain chemotherapeutic drugs that are inactive until metabolized by the enzyme. This strategy has been applied to deliver selective chemotherapy, where probiotics produce the necessary enzymes to convert prodrugs into their active form directly at the tumor site, thereby enhancing the therapeutic effect while minimizing systemic toxicity 119. Apart from directly delivering therapeutic molecules, engineered probiotics can also influence the body's immune system through their microbiome-modulating properties. The gut microbiome is crucial in controlling immune functions, and disruptions in its balance have been associated with several diseases, including cancer. Engineered probiotics can be designed to restore microbiome balance, improve gut health, and boost systemic immune function 120.

For example, engineered probiotics that produce short-chain fatty acids (SCFAs), such as butyrate, have been shown to have anti-inflammatory and immunomodulatory effects. Butyrate is known to enhance the activity of regulatory T cells (Tregs), which play a critical role in preventing autoimmune reactions while maintaining immune homeostasis. In cancer therapy, the use of probiotics that generate butyrate can help modulate the immune response, enhancing anti-tumor immunity while preventing excessive inflammation 121. Furthermore, probiotics can be engineered to produce antimicrobial peptides (AMPs), which can selectively target and kill cancer cells. AMPs have shown promise in preclinical studies as cancer therapeutics due to their ability to selectively disrupt tumor cell membranes. By engineering probiotics to produce AMPs in response to specific inducers, researchers can direct the localized production of these molecules at the tumor site, enhancing the therapeutic effect and reducing off-target toxicity 122.

Engineered probiotics can deliver a wide range of therapeutic modalities, including immune modulators, anticancer drugs, enzymes, and genetic materials. These modalities can be precisely targeted to disease sites, enhancing therapeutic efficacy while minimizing systemic side effects. Such versatility makes engineered probiotics a promising platform for innovative and personalized cancer therapies.

### 5.1. Cytokine delivery

The use of engineered probiotics to deliver cytokines, such as interleukin-2 (IL-2) and granulocyte-macrophage colony-stimulating factor (GM-CSF), has gained significant attention in recent years as a promising approach in cancer immunotherapy. Cytokines are signaling molecules that play a crucial role in modulating the immune system, particularly in stimulating immune responses against tumors 123. The delivery of cytokines to the tumor microenvironment (TME) using engineered probiotics offers a controlled and localized means of enhancing immune activation while minimizing systemic side effects. This targeted approach can improve the efficacy of cancer therapies, as the cytokines can stimulate the immune system directly within the tumor or its surrounding tissue, thereby increasing the likelihood of tumor eradication 124.

Interleukin-2 (IL-2) is one of the most well-known cytokines used in cancer immunotherapy. It is a key regulator of T-cell activation and proliferation, particularly for the expansion of effector T cells and the activation of natural killer (NK) cells. IL-2 has been used in clinical settings to treat various cancers, including melanoma and renal cell carcinoma. However, despite its potential, systemic administration of IL-2 is often associated with severe side effects, such as capillary leak syndrome, which limits its therapeutic application. The localized delivery of IL-2 by engineered probiotics can mitigate these issues by ensuring that IL-2 is released in proximity to the tumor, where its effects can be maximized while minimizing off-target effects. Several studies have explored the use of engineered probiotics for the delivery of IL-2 125. One such study by Lien* et al*. 2019 demonstrated that genetically modified *Lactobacillus* species could be engineered to express IL-2 in response to acidic pH, a feature characteristic of the TME. This system allowed for the localized production of IL-2 at the tumor site, where it could enhance T-cell activity and promote anti-tumor immunity. By using engineered probiotics as IL-2 delivery vehicles, researchers were able to target the TME more effectively and reduce the adverse effects associated with systemic IL-2 administration 126. Moreover, the probiotic-based delivery system also allowed for a more sustained release of IL-2, which may contribute to prolonged immune activation in the tumor microenvironment.

Granulocyte-macrophage colony-stimulating factor (GM-CSF) is another cytokine that plays a pivotal role in the immune response, particularly in the activation and differentiation of dendritic cells (DCs) and macrophages. GM-CSF is crucial for promoting the maturation of DCs, which are key antigen-presenting cells that initiate and regulate immune responses 127. By inducing the maturation of DCs, GM-CSF helps prime the immune system to recognize and attack tumor cells more effectively. Additionally, GM-CSF has been shown to enhance the activation of both CD4+ T helper cells and CD8+ cytotoxic T cells, further boosting anti-tumor immunity. However, like IL-2, systemic administration of GM-CSF is often associated with undesirable side effects, such as fever, bone pain, and leukocytosis. To overcome these limitations, the delivery of GM-CSF using engineered probiotics offers a targeted and controlled approach. In one study, *Lactococcus lactis* was genetically modified to produce GM-CSF in response to specific inducers, such as nutrient availability or changes in environmental conditions. The engineered *Lactococcus lactis* was shown to effectively promote dendritic cell maturation *in vitro* and induce a potent immune response in animal models 128. This approach allowed for localized cytokine production within the tumor microenvironment, where GM-CSF could enhance immune cell recruitment and activation without causing systemic toxicity.

The use of engineered probiotics for cytokine delivery also has the potential to improve the safety and efficacy of combination therapies. For example, IL-2 and GM-CSF have been investigated in combination with other immunotherapeutic strategies, such as immune checkpoint inhibitors and cancer vaccines. Combining IL-2 or GM-CSF with immune checkpoint inhibitors, like anti-PD-1 antibodies, can enhance the immune system's ability to overcome tumor-induced immunosuppression and promote sustained immune responses 129. Engineered *Lactobacillus* producing IL-2, combined with anti-PD-1 antibody treatment, notably enhanced tumor suppression in melanoma mouse models. The probiotic-based IL-2 delivery system promoted T-cell expansion and activation, while the checkpoint inhibitor blocked the inhibitory signals from the tumor, resulting in enhanced anti-tumor immunity 130.

Additionally, cytokine delivery via engineered probiotics can help address the challenges associated with the tumor heterogeneity and the immunosuppressive TME. Tumors typically contain a variety of immune cells, such as regulatory T cells (Tregs) and myeloid-derived suppressor cells (MDSCs), which aid in evading immune detection and support tumor growth. Cytokines like IL-2 can influence the tumor microenvironment by boosting the function of effector T cells while reducing the suppressive actions of Tregs and MDSCs 131. Research by Li* et al*. 2017 demonstrated that *Lactobacillus* engineered to produce IL-2 was able to reshape the TME by increasing the ratio of effector T cells to Tregs, leading to improved anti-tumor immunity in a mouse model of breast cancer. This approach not only enhanced the immune response against the tumor but also helped overcome the immunosuppressive effects of the TME, which is a significant challenge in cancer immunotherapy 132.

Furthermore, the use of probiotics to deliver cytokines like IL-2 and GM-CSF provides a mechanism for sustained, on-demand cytokine production. Unlike traditional cytokine therapy, where cytokine administration is typically a one-time or intermittent process, probiotic-based delivery systems allow for the continuous production of cytokines within the TME. This sustained release of cytokines can potentially overcome the limitations associated with transient cytokine therapies, such as inadequate cytokine levels or rapid clearance from the body. The probiotic-mediated delivery system also allows for more precise control over the timing and dosage of cytokine release, which is essential for maximizing therapeutic efficacy and minimizing adverse effects 133.

The development of probiotic-based cytokine delivery systems has also benefited from advancements in synthetic biology and genetic engineering techniques. Tools such as CRISPR-Cas9 and synthetic promoters enable the precise modification of probiotics to express cytokines in response to specific environmental signals. For instance, researchers have engineered *E. coli* and *Lactobacillus* species to produce cytokines in response to changes in temperature, pH, or the presence of specific substrates. This level of control over cytokine production allows for highly targeted and responsive therapeutic strategies that can be tailored to individual tumors and patients 134. Moreover, the use of probiotics as cytokine delivery vectors offers several advantages over traditional cytokine therapies. Probiotics are generally considered safe and well-tolerated, with a long history of use in human health. The ability to modify probiotics to produce and deliver cytokines also opens up new possibilities for combination therapies, where probiotics could be engineered to deliver multiple cytokines or other immune-modulatory factors in a synergistic manner. This strategy could help maximize the anti-tumor immune response while minimizing the need for multiple drug regimens, thus improving patient compliance and reducing treatment costs 135.

**Figure 12:**
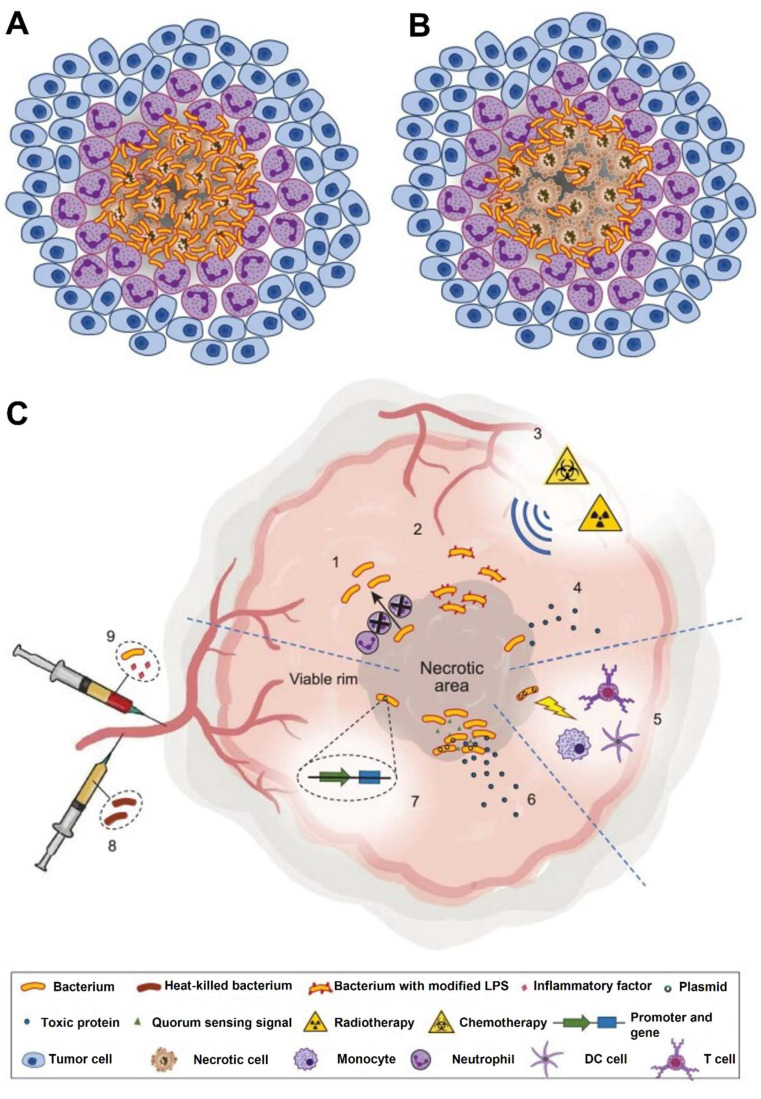
**(A)** A tumor's bacterial dispersion pattern. A tumor's necrotic/hypoxic region is mostly colonized by bacteria, which can do so in one of two ways: either uniformly across the necrotic area or **(B)** accumulating in the hypoxic area with a few colonies deeper in the necrotic area. In order to encircle the bacteria, neutrophils congregate around the necrotic region and may overlap with the bacterial area. **(C)** Suggestions for improving bacterial treatment. Three categories can be applied to the approaches. One is to stop the neutrophil ring from forming in order to target the tumor's viable region. Reused under Creative Commons Attribution License from ref. 136.

Li et al. 2021 explore how bacterial colonization within tumors establishes a spatially organized “hunting field” that orchestrates immune-tumor interactions. They report that, following administration of bacteria such as *Salmonella*, *Clostridium*, *E. coli*, and *Pseudomonas* in preclinical models, microbes preferentially colonize necrotic or hypoxic tumor regions. Surrounding these bacterial clusters, neutrophils form ring-like structures that act as an initial immune barrier. This arrangement peaks 1-3 days post-injection and stabilizes into two main distribution patterns: either a uniform spread within necrotic zones or localized aggregation near hypoxic rims. The review likens this phenomenon to a hunting scenario: bacteria (the “rabbits”) and innate immune cells (the “dogs”) infiltrate the tumor, activating adaptive immune cells (the “tigers”) and creating a congested microenvironment where both cancer cells (“sheep”) and bacteria are targeted **(Figure 12)**. This dynamic “hunting field” stimulates immune infiltration and reactivation, enhances exposure of tumor neoantigens, and may potentiate anti-tumor immunity by unmasking adaptive responses 136.

Yan F & Polk DB 2002, explores in his study that how the probiotic bacterium *Lactobacillus rhamnosus* GG (LGG) contributes to maintaining intestinal cell health by protecting cells from apoptosis triggered by pro-inflammatory cytokines. Although probiotics are widely used to support gut health, the exact cellular mechanisms by which they exert their effects are not fully understood. This research focuses on understanding how LGG interacts with intestinal epithelial cells to promote their survival, especially in conditions where inflammatory cytokines such as tumor necrosis factor (TNF), interleukin-1α (IL-1α), and interferon-gamma (IFN-γ) are present. The findings demonstrate that LGG is capable of preventing cytokine-induced apoptosis in both mouse and human intestinal epithelial cell lines. A key mechanism involves the activation of the Akt/protein kinase B pathway, which is known to play a crucial role in promoting cell survival. Simultaneously, LGG inhibits the activation of the p38 mitogen-activated protein kinase (MAPK) pathway, which is typically associated with promoting apoptosis in response to stress or inflammatory signals. These dual effects stimulating a survival pathway and blocking a death pathway suggest that LGG helps protect intestinal cells from damage caused by an inflammatory environment. Interestingly, not only live bacteria but also substances secreted by LGG into the culture medium are shown to have similar protective effects. Supernatants from LGG cultures, when applied to intestinal cells, activated the Akt pathway and reduced apoptosis in a concentration-dependent manner 137. This implies that LGG produces soluble factors capable of influencing epithelial cell signaling and survival without requiring direct contact. Overall, the study provides insight into a previously underappreciated mechanism by which probiotics like LGG may support intestinal health by modulating intracellular signaling pathways that regulate cell death and survival in the face of inflammation.

### 5.2 Tumor antigen presentation and vaccine platforms

Tumor antigen presentation is a critical step in the initiation of the immune response against cancer, and engineered probiotics have emerged as a promising platform for enhancing this process. Tumor antigens, which are specific proteins or peptides expressed on the surface of tumor cells, serve as markers for the immune system to identify and target cancer cells 138. However, the effective presentation of these antigens to the immune system is often hindered by various factors, such as the immunosuppressive tumor microenvironment (TME), the presence of regulatory cells, and immune tolerance. Vaccines targeting tumor antigens aim to prime the immune system, particularly T cells, to recognize and eliminate tumor cells. By engineering probiotics to present tumor antigens and deliver adjuvants, researchers have developed novel vaccine platforms capable of enhancing tumor-specific immunity 139.

Probiotics are gaining attention as promising carriers for delivering tumor-associated antigens (TAAs) and tumor-specific antigens (TSAs) because of their ability to target gut-associated lymphoid tissue (GALT) and stimulate mucosal immunity. Since the GALT is a key site for initiating immune responses, especially in mucosal tissues, probiotics serve as an effective platform for presenting tumor antigens to the immune system 140. Engineered probiotics can produce and display these antigens, triggering both local mucosal and systemic immune reactions. Beyond antigen delivery, probiotics can also transport immune adjuvants that boost the strength and longevity of cancer vaccine responses. Among these, *Lactobacillus* species stand out due to their well-documented safety profile (generally recognized as safe, or GRAS) and extensive use in microbiome research. These bacteria naturally engage the immune system by activating dendritic cells and T cells, which are vital for antigen presentation and initiating anti-tumor immunity. By genetically engineering *Lactobacillus* to express tumor-specific antigens, researchers have explored its potential as an effective therapeutic vaccine platform 141.

In one study, *Lactobacillus rhamnosus* was engineered to express a fragment of the human epidermal growth factor receptor 2 (HER2), which is overexpressed in certain cancers, including breast cancer 142. The engineered *Lactobacillus* was able to deliver the HER2 antigen to dendritic cells in the gut-associated lymphoid tissue, which in turn activated a systemic immune response. The vaccination resulted in the activation of HER2-specific T cells and the inhibition of tumor growth in mouse models of breast cancer. This approach demonstrated the potential of engineered probiotics to present tumor antigens in a localized and efficient manner, thereby enhancing the immune system's ability to recognize and target tumor cells 143.

Another promising tumor antigen presentation strategy involves the use of *E. coli* strains engineered to display tumor antigens on their surface. This platform, known as bacterial surface display, involves the genetic modification of bacteria to express tumor antigens as fusions with surface proteins, which allows the antigens to be presented to immune cells in a form that mimics their natural presentation by tumor cells. Surface display of tumor antigens on engineered probiotics has been shown to trigger a potent immune response, as it allows for the direct interaction of the antigen with antigen-presenting cells (APCs), such as dendritic cells, which are crucial for initiating T cell-mediated immunity 144. A notable example of bacterial surface display for tumor antigen presentation is the use of *E. coli* engineered to express the prostate-specific antigen (PSA) on its surface. PSA is a tumor-associated antigen commonly found in prostate cancer cells. The engineered *E. coli* strain, when administered to mice, led to the activation of PSA-specific CD8+ T cells, which in turn targeted and destroyed PSA-expressing tumor cells *in vivo* 145. This approach highlighted the potential of probiotics as vaccine platforms for presenting tumor antigens and triggering a targeted immune response.

In addition to expressing tumor antigens, engineered probiotics can also be used to deliver immune adjuvants to enhance the presentation and recognition of tumor antigens. Adjuvants are substances that stimulate the immune system and increase the effectiveness of vaccines by enhancing antigen presentation, promoting the activation of immune cells, and prolonging the immune response. Some adjuvants, such as the synthetic toll-like receptor (TLR) agonists, can be delivered by engineered probiotics to improve the efficacy of tumor antigen-based vaccines 146.

For example, *Lactobacillus* has been engineered to produce and deliver TLR agonists, such as monophosphoryl lipid A (MPLA), which activates TLR4 and enhances the immune response. When used in combination with tumor antigens, such as the melanoma-associated antigen gp100, this probiotic-based vaccine platform was shown to induce a stronger anti-tumor immune response and increase tumor cell death in preclinical models 147. By combining antigen presentation with immune stimulation, engineered probiotics have the potential to overcome the challenges posed by the TME and promote sustained immune activation. The application of probiotic-based tumor vaccines also extends to the development of live attenuated vaccines, which use weakened forms of bacteria to stimulate immune responses without causing disease. Live attenuated probiotics, such as *Lactococcus lactis*, have been engineered to express tumor antigens and deliver them to the immune system while simultaneously providing immune modulation through their interaction with immune cells. The use of live probiotics as tumor antigen carriers provides the advantage of continuous, localized antigen presentation, which can result in more robust and long-lasting immunity 148.

**Figure 13:**
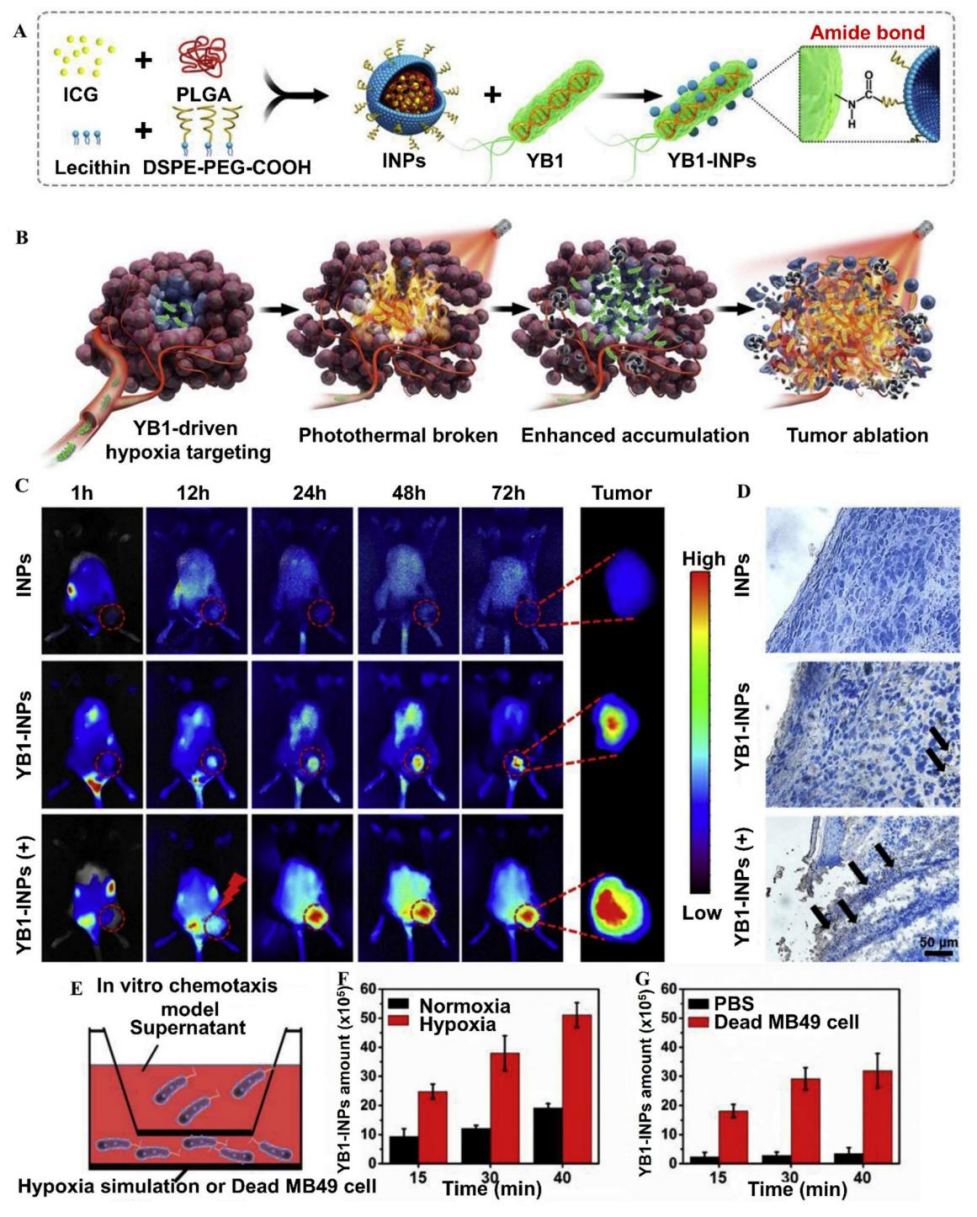
(A) YB1-INP preparation process. INPs that were synthesized via a single-step sonication process were joined to YB1 by amide bonds. (B) YB1-INPs for tumor penetrative treatment that target hypoxia and bioaccumulate with the help of photothermal. (C) FL imaging of INPs, YB1-INPs, and YB1-INPs (+) *in vivo* at various intervals. (+) denotes laser irradiation for 12 hours to encourage YB1-INP bioaccumulation. The tumors' *ex vivo* NIR FL images at 72 hours. (D) Tumor segment immunohistochemistry pictures after 72 hours. The findings demonstrated that photothermally disturbed tumors have increased YB1-INP bioaccumulation. The colonies of YB1-INPs are shown by the black dots with arrows. (E) Diagrammatic representation of the nutritional and hypoxic simulation model that assesses YB1-INP chemotaxis utilizing a transwell device. (F,G) In the hypoxia-induced (F) and nutrition-induced (G) conditions (dead MB49 cells following photothermal treatment), YB1-INPs migrate to the bottom compartment. LB agar plate test and flow cytometry were used to count the cells. Adapted with permission from ref. 149 Copyrights 2019, Elsevier.

Chen* et al*. 2019 engineered a hybrid biotic-abiotic system (YB1-INPs) by covalently attaching indocyanine green loaded nanoparticles (INPs) to a hypoxia-seeking *Salmonella Typhimurium* strain (YB1), creating nanophotosensitizer-equipped bacteria that retain viability and self-propulsion into tumor cores. This YB1-INP complex specifically accumulates in hypoxic regions of large solid tumors (> 500 mm³), enabling near-infrared (NIR) light-activated photothermal therapy (PTT) with robust fluorescence imaging capability. Notably, near-infrared (NIR) irradiation not only causes direct tumor cell destruction through hyperthermia but also increases the local accumulation of YB1 INPs by about 14-fold, likely because tumor tissue disruption and nutrient release attract more bacteria nanoparticle complexes to the area **(Figure 13)**. In murine models, a single treatment eradicated large tumors without relapse, demonstrating potent, low-toxicity efficacy and highlighting a promising strategy that combines hypoxia targeting, imaging-guided PTT, and self-amplifying accumulation for sizable solid tumor therapy 149.

In one study, *Lactococcus lactis* was engineered to express the tumor antigen mucin 1 (MUC1), a glycoprotein overexpressed in various cancers, including breast and ovarian cancers. The engineered *Lactococcus lactis* was able to deliver MUC1 to dendritic cells in the lymph nodes, which in turn activated CD4+ and CD8+ T cells. The vaccination resulted in a significant reduction in tumor size in mouse models of MUC1-expressing cancers, demonstrating the potential of engineered probiotics as live bacterial vaccines for cancer immunotherapy 150. In addition to surface display and live vaccines, engineered probiotics can also be used to deliver tumor antigens via secretion systems. Some probiotics have natural secretion systems that allow them to release proteins or peptides into the surrounding environment. By harnessing these systems, researchers have engineered probiotics to secrete tumor antigens directly into the tumor microenvironment, where they can be captured by local dendritic cells and presented to T cells. This targeted delivery of antigens can help enhance the specificity and potency of cancer vaccines 151.

In a study by Li* et al*. 2020, *Lactobacillus casei* was engineered to secrete the tumor antigen ovalbumin (OVA) into the TME. The secretion of OVA by the probiotic strain led to the recruitment and activation of tumor-specific T cells, which successfully eradicated OVA-expressing tumors in animal models. This study demonstrated the feasibility of using probiotic secretion systems for targeted antigen delivery and highlighted the advantages of localized delivery for enhancing tumor immunotherapy. Overall, the use of engineered probiotics as tumor antigen presentation platforms offers several advantages over traditional cancer vaccines. These include the ability to provide localized antigen delivery, enhance immune activation through the production of immune adjuvants, and offer a more personalized and sustained approach to cancer immunotherapy. As the field of synthetic biology continues to advance, the potential for probiotic-based tumor vaccines to provide safer and more effective cancer treatments is increasingly becoming a reality 152.

**Figure 14:**
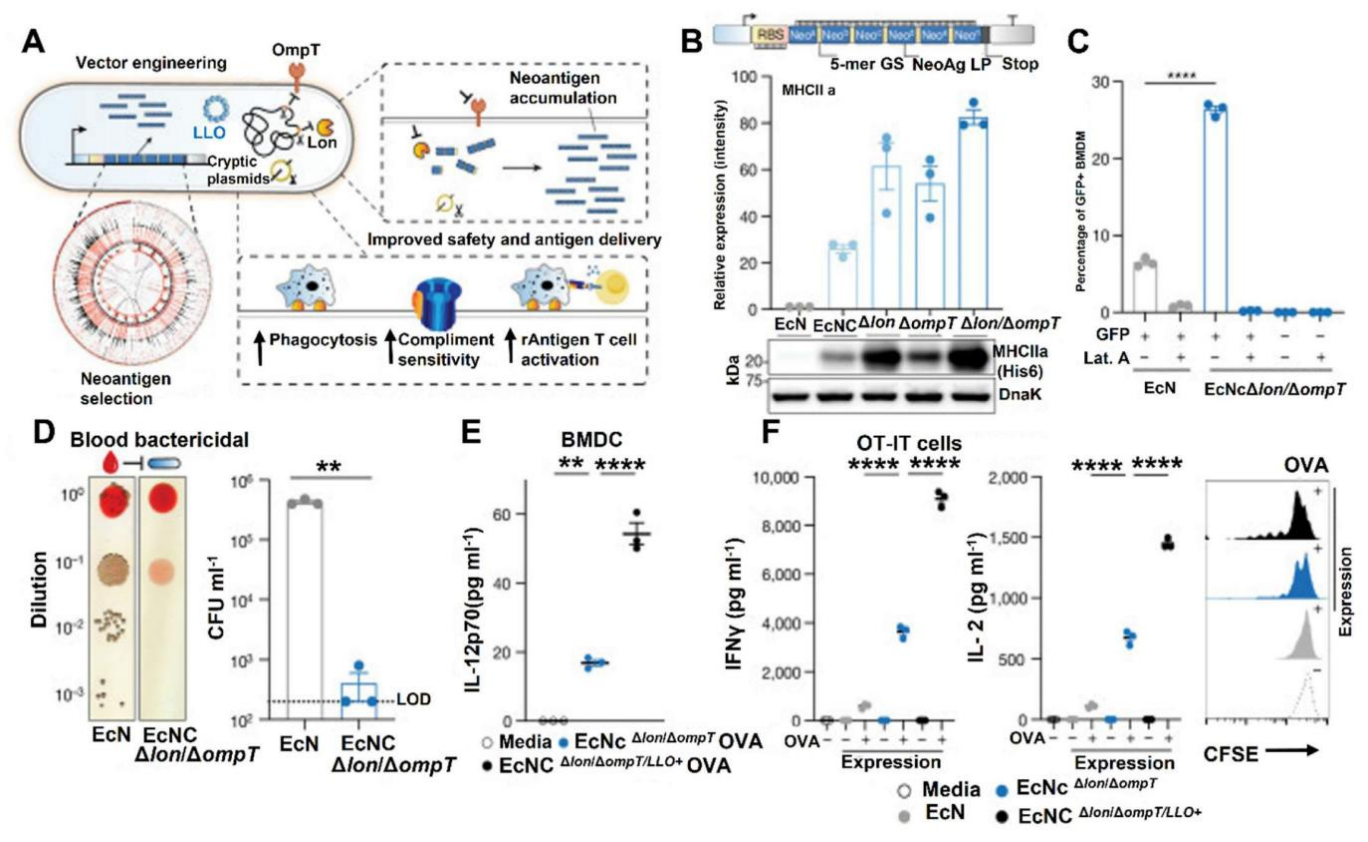
Development and Evaluation of Microbial Tumor Neoantigen Vaccines **(A)** Illustration of microbial neoantigen vaccine design, accompanied by a Circos plot representing the mutanome of CT26 tumors. **(B)** Top panel shows a schematic of the optimized synthetic neoantigen construct. The middle panel presents relative chemiluminescent intensities from immunoblots assessing MHCIIa neoantigen construct expression in *E. coli* Nissle (EcN) and its engineered derivatives (n = 3 independent biological replicates per group). The bottom panel includes a representative immunoblot displaying MHCIIa expression levels in a selected strain. **(C)** Quantification of GFP-positive bone marrow-derived macrophages (BMDMs) after exposure to either EcN or EcN^cΔlon/ΔompT strains constitutively expressing GFP (n = 3). Latrunculin A (Lat. A) was used for actin disruption. **(D)** Left panel shows representative images of EcN and EcN^cΔlon/ΔompT strains spotted on LB agar plates post-incubation in human blood. The right panel quantifies microbial load in colony-forming units per milliliter (CFU ml⁻¹) (n = 3). The detection limit was set at 2 × 10² CFU ml⁻¹. **(E)** Measurement of IL-12p70 levels in supernatants from bone marrow-derived dendritic cells (BMDCs) after antigen pulsing (n = 3). **(F)** Naive OT-I T cells were co-cultured with pulsed BMDCs. Left and middle panels display IFN-γ and IL-2 levels, respectively, in culture supernatants (n = 3). The right panel provides a representative histogram showing CFSE dilution as an indicator of OT-I T cell proliferation. Reused under Creative Commons Attribution License from ref. 153.

Redenti* et al.* 2024 developed a modified strain of *E. coli* Nissle 1917 (EcN) designed to produce and deliver arrays of neoepitope-containing peptides derived from tumor-specific mutations. To enhance the safety and efficacy of this system, the bacteria were engineered to have increased susceptibility to blood clearance and phagocytosis, ensuring they are effectively processed by the immune system. Additionally, listeriolysin O (LLO) was expressed to enable the delivery of neoantigens into the cytosol, enhancing their presentation through major histocompatibility complex (MHC) class I pathways and triggering a robust CD8+ T cell response. In murine models of advanced primary and metastatic solid tumors, administration of these engineered probiotics led to significant tumor regression and extended survival. The treatment induced a comprehensive immune response characterized by the activation of dendritic cells, extensive priming of neoantigen-specific CD4+ and CD8+ T cells, and broader activation of both T and natural killer (NK) cells. Moreover, there was a notable reduction in immunosuppressive cell populations within the tumor microenvironment, including regulatory T and B cells and myeloid-derived suppressor cells. This approach leverages the natural tumor-homing properties of certain bacteria and their capacity to be synthetically engineered, offering a promising avenue for personalized cancer vaccines **(Figure 14)**. By delivering tumor-specific neoantigens directly within the tumor microenvironment, this strategy aims to overcome the limitations of traditional cancer vaccines and immunotherapies, potentially leading to more effective and durable antitumor immunity 153.

### 5.3. Immune checkpoint inhibitor delivery (e.g., anti-PD-L1 nanobodies)

The use of engineered probiotics for the delivery of immune checkpoint inhibitors (ICIs) has emerged as a promising strategy in cancer immunotherapy. Immune checkpoint inhibitors, such as anti-PD-L1 (programmed death-ligand 1) nanobodies, have revolutionized cancer treatment by blocking inhibitory signals that suppress the immune response against tumors. By targeting immune checkpoints, these therapies can enhance T-cell activity, overcome tumor-induced immune evasion, and improve anti-tumor immunity. However, systemic delivery of immune checkpoint inhibitors (ICIs) frequently leads to notable side effects because of widespread immune system activation. Engineered probiotics offer a potential solution by delivering ICIs directly to the tumor microenvironment (TME), thereby reducing systemic toxicity while enhancing localized immune responses 154.

The programmed cell death protein 1 (PD-1) pathway, which includes the interaction between PD-1 on T cells and PD-L1 on tumor cells, is one of the primary mechanisms of immune evasion in cancer. PD-L1 binding to PD-1 inhibits T-cell activation, leading to immune tolerance and allowing tumors to evade immune surveillance. Immune checkpoint inhibitors, such as anti-PD-L1 antibodies, block this interaction and restore the ability of T cells to recognize and destroy tumor cells. However, systemic administration of anti-PD-L1 antibodies can lead to immune-related adverse events, such as autoimmune disorders, which limit their therapeutic potential 155. To mitigate these side effects, the delivery of immune checkpoint inhibitors via engineered probiotics presents a promising alternative. Probiotics, particularly those from the *Lactobacillus* and *Bifidobacterium* genera, have been studied for their ability to modulate immune responses through interactions with the gut-associated lymphoid tissue, which plays a pivotal role in initiating immune responses. By engineering probiotics to produce and deliver immune checkpoint inhibitors, researchers aim to create a localized delivery system that targets the tumor site directly, thereby enhancing the specificity of the therapy and minimizing off-target effects 156.

One of the most intriguing approaches is the use of nanobodies, a novel class of small, single-domain antibodies derived from camelid species. Nanobodies are smaller, more stable, and more efficient than conventional antibodies, making them an attractive choice for cancer immunotherapy 157. In a study by Park* et al*. 2017, anti-PD-L1 nanobodies were expressed in *Lactobacillus* species and used for targeted delivery to the TME. These engineered probiotics were able to specifically bind to PD-L1 and block its interaction with PD-1 on T cells, leading to enhanced anti-tumor immune responses in preclinical models. The use of nanobodies allowed for more efficient binding and a higher affinity for PD-L1 compared to traditional antibodies, which could potentially increase the therapeutic efficacy of the treatment 158.

**Figure 15:**
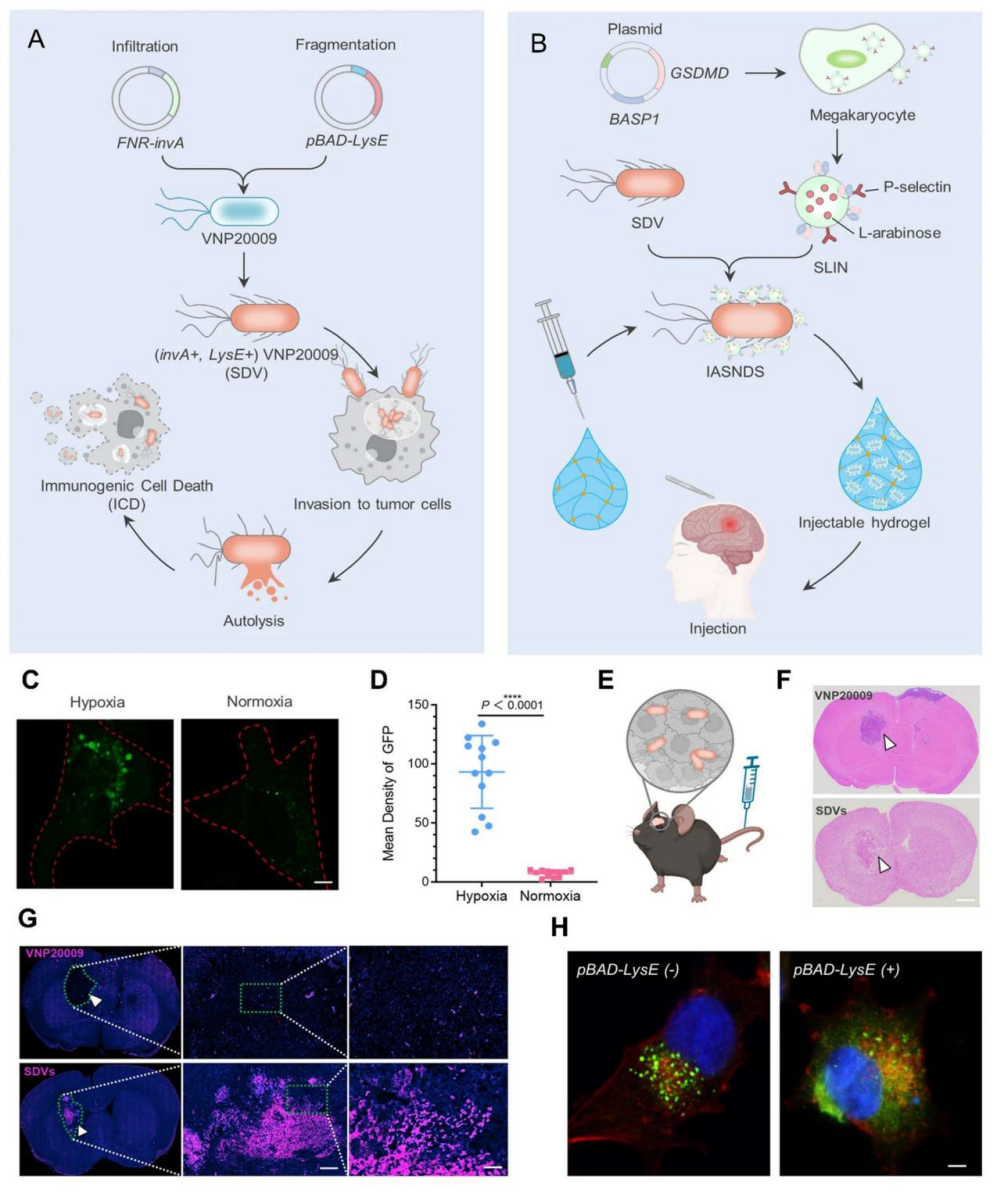
**(A)** The schematic illustrates how *Salmonella* VNP20009 strategically transforms into the SDV and induces ICD in GBM cells. **(B)** An illustration showing the IASNDS preparation and the hydrogel's synergistic localized treatment for use in surgical cavities. **(C)** Fluorescence (GFP+) in the SDV (green) is seen over 24 hours in both normoxic and hypoxic circumstances. GL261 cell borders are shown by red dashed lines. (n = three separate experiments). **(D)** A statistical study that displays the GFP fluorescence intensity in cultures that are normoxic and hypoxic. (n = 12 pictures from three different tests). **(E)** and **(F)** Evaluation of the *in vivo* dispersion of 1 × 10^7 CFU SDVs (GFP-) administered by tail vein injection into mice with intracranial tumors. Sectioning of brain GBM tissue was done; a white arrow represents the SDVs. **(G)** Additional brain tissue sections were stained with fluorescence to see the distribution of SDV (GFP-, red) in intracranial GBM tissues (white arrows). (n = three separate experiments). **(H)** Illustration of the SDVs' (GFP+) green fluorescence distribution under pBAD-LysE (-) and pBAD-LysE (+) conditions when 500 μM L-arabinose was added to the cell culture medium for 24 hours (n = 3 separate studies). In the pBAD-LysE (+) condition, the intracellular GFP distribution shows that SDV autolysis has begun in GL261 cells. Reused under Creative Commons Attribution License from ref. 159.

Zhang* et al*. 2024 developed an innovative post-surgical immunotherapeutic strategy for glioblastoma by designing a cavity-injectable bacterium-hydrogel superstructure that leverages *Salmonella typhimurium* (strain VNP20009) to stimulate potent anti-GBM immunity and prevent tumor recurrence in mice. The engineered treatment consists of *Salmonella* delivery vehicles (SDVs) tethered with *Salmonella* lysis-inducing nanocapsules (SLINs) that trigger bacterial self-lysis within the tumor cavity, releasing immunogenic bacterial components and inducing pyroptosis of residual glioma cells. When embedded in an ATP-responsive hydrogel matrix, this formulation homes to the surgical cavity, recruits phagocytes, enhances antigen presentation, and boosts both innate and adaptive immune responses, including T-cell activation. In murine GBM models, this localized bacteriotherapy significantly reduced postoperative relapse rates by transforming the resection zone into an immunologically active microenvironment, illustrating its promise as a new approach to forestall glioblastoma recurrence **(Figure 15)** 159.

Furthermore, the small size and high stability of nanobodies make them ideal candidates for incorporation into probiotic-based delivery systems. These properties allow for the prolonged activity of the nanobodies in the TME, potentially resulting in a more sustained immune response. In one example, researchers engineered *Bifidobacterium breve* to secrete anti-PD-L1 nanobodies in response to specific environmental cues, such as low pH in the TME. The engineered *B. breve* strain successfully inhibited the PD-1/PD-L1 interaction, promoting T-cell activation and tumor regression in mouse models of melanoma 160. Another significant advantage of probiotic-mediated delivery of immune checkpoint inhibitors is the potential for sustained, on-demand production of the therapeutic molecules. By engineering probiotics to produce ICIs continuously or in response to specific environmental triggers, researchers can achieve more localized and controlled delivery of these therapeutics. This approach contrasts with traditional administration methods, which often involve periodic doses and the risk of fluctuations in drug levels. The continuous release of anti-PD-L1 nanobodies from engineered probiotics provides a more consistent and localized inhibition of the PD-1/PD-L1 pathway, thereby enhancing the overall therapeutic effect 161.

In addition to direct inhibition of the PD-1/PD-L1 interaction, probiotic delivery of immune checkpoint inhibitors has been combined with other immunotherapeutic strategies to further enhance anti-tumor responses. For example, probiotics have been engineered to co-deliver immune checkpoint inhibitors along with other immune-stimulating agents, such as cytokines or co-stimulatory molecules. In one study, *Lactobacillus* was engineered to produce both anti-PD-L1 nanobodies and IL-12, a potent cytokine that promotes T-cell activation and the recruitment of immune cells to the tumor site. The combination of these two agents resulted in a synergistic effect, with the anti-PD-L1 nanobodies enhancing T-cell activation and the IL-12 further boosting the immune response, leading to significant tumor regression in mouse models 162.

**Figure 16:**
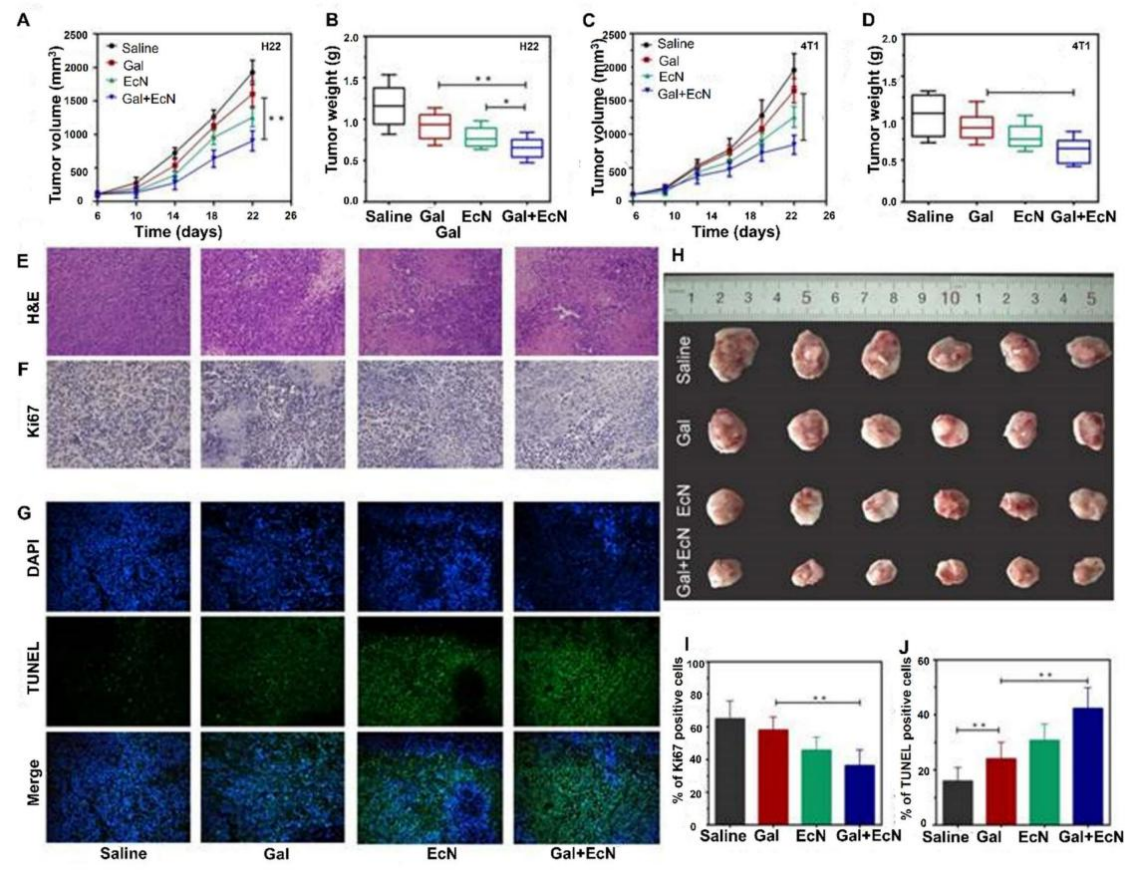
In tumor-bearing mice, EcN improved the anti-tumor effects of TGF-β inhibition. **(A)** Growth of tumors in mice with H22 hepatic carcinoma cells (n = 6). **(B)** At the conclusion of the experiment, the tumor weight of mice harboring H22 hepatic carcinoma cells. **(C)** Growth of tumors in mice with 4T1 breast cancer cells (n = 6). **(D)** At the conclusion of the experiment, the tumor weight of mice harboring 4T1 breast cancer cells. **(E to G)** Data are typical of two separate investigations (n = 3) and show the histological examination of tumor tissues stained by H&E, Ki67, DAPI (blue), and TUNEL (green) at 200×. **(H)** At the conclusion of the experiment, representative pictures of tumor tissues. **(I)** Using the Ki67 staining technique, the tumor tissues proliferation index is semi-quantified. **(J)** TUNEL staining test semi-quantification of the tumor tissue's apoptosis index. Reused under Creative Commons Attribution License from ref. 163.

Shi* et al*. 2019 present a compelling preclinical investigation demonstrating that oral administration of the probiotic *E. coli* strain Nissle 1917 (EcN) significantly potentiates the anticancer efficacy of the TGF-β receptor I kinase inhibitor galunisertib (Gal) in murine models of breast (4T1) and liver (H22) cancers. While Gal alone exerted only modest suppression of tumor growth and metastasis, its combination with daily EcN dramatically enhanced tumor control, reduced metastatic burden, and improved overall survival. Flow cytometry and histological analysis revealed that EcN synergized with Gal to modulate the tumor microenvironment: there was a notable increase in tumor-specific effector CD8⁺ T cell infiltration, activation of dendritic cells, and a reduction in immunosuppressive features such as regulatory T cells and myeloid-derived suppressor cells. Mechanistically, the combination therapy alleviated the immunosuppressive milieu established by TGF-β signaling, re-educating the tumor immune microenvironment towards a more immunostimulatory phenotype. Moreover, 16S rRNA sequencing of fecal samples demonstrated that EcN reshaped the gut microbiome, enriching for beneficial commensals, and fecal microbiota transplantation experiments confirmed that these microbial changes contribute to the enhanced anticancer effect. Notably, the combined regimen also suppressed tumor invasiveness and metastasis in both mouse and zebrafish xenograft models, underscoring its potential breadth of application **(Figure 16)**. The study concludes that integrating probiotic modulation of gut microbiota with TGF-β blockade represents a novel and promising strategy to overcome resistance and amplify antitumor immunity, offering important implications for future cancer immunotherapy development 163.

Moreover, engineered probiotics offer the advantage of targeted delivery, which is particularly important in overcoming the challenges posed by the tumor microenvironment. The TME is often characterized by immunosuppressive factors, such as regulatory T cells (Tregs) and myeloid-derived suppressor cells (MDSCs), which can inhibit the effectiveness of immune checkpoint inhibitors. The localized delivery of anti-PD-L1 nanobodies via engineered probiotics can help overcome some of these immunosuppressive factors by directly targeting and blocking PD-L1 in the TME, thereby allowing for more effective immune activation. This localized approach can also minimize the risk of immune-related adverse events, as the immune checkpoint inhibitors are delivered directly to the tumor site and are less likely to cause systemic immune activation 164.

The use of probiotics as delivery systems for immune checkpoint inhibitors (ICIs) goes beyond just anti-PD-L1 nanobodies and can include other ICIs like anti-CTLA-4 antibodies. Engineering probiotics to carry multiple ICIs simultaneously offers a more thorough way to modulate the immune system by targeting several checkpoint pathways, potentially boosting the effectiveness of cancer immunotherapy. For instance, scientists have modified *Lactococcus lactis* to produce both anti-PD-L1 nanobodies and anti-CTLA-4 antibodies, resulting in a strong synergistic effect that significantly enhanced tumor suppression in breast cancer animal models 165. Overall, the delivery of immune checkpoint inhibitors, such as anti-PD-L1 nanobodies, through engineered probiotics offers several advantages over traditional delivery methods. The localized and controlled delivery of these inhibitors to the TME can enhance their efficacy while minimizing systemic side effects. The use of probiotics as delivery vehicles also allows for the continuous, on-demand production of immune checkpoint inhibitors, providing sustained therapeutic effects. As research in this area progresses, probiotic-based delivery systems have the potential to become a valuable tool in cancer immunotherapy, improving the efficacy and safety of immune checkpoint inhibitors 166.

**Figure 17:**
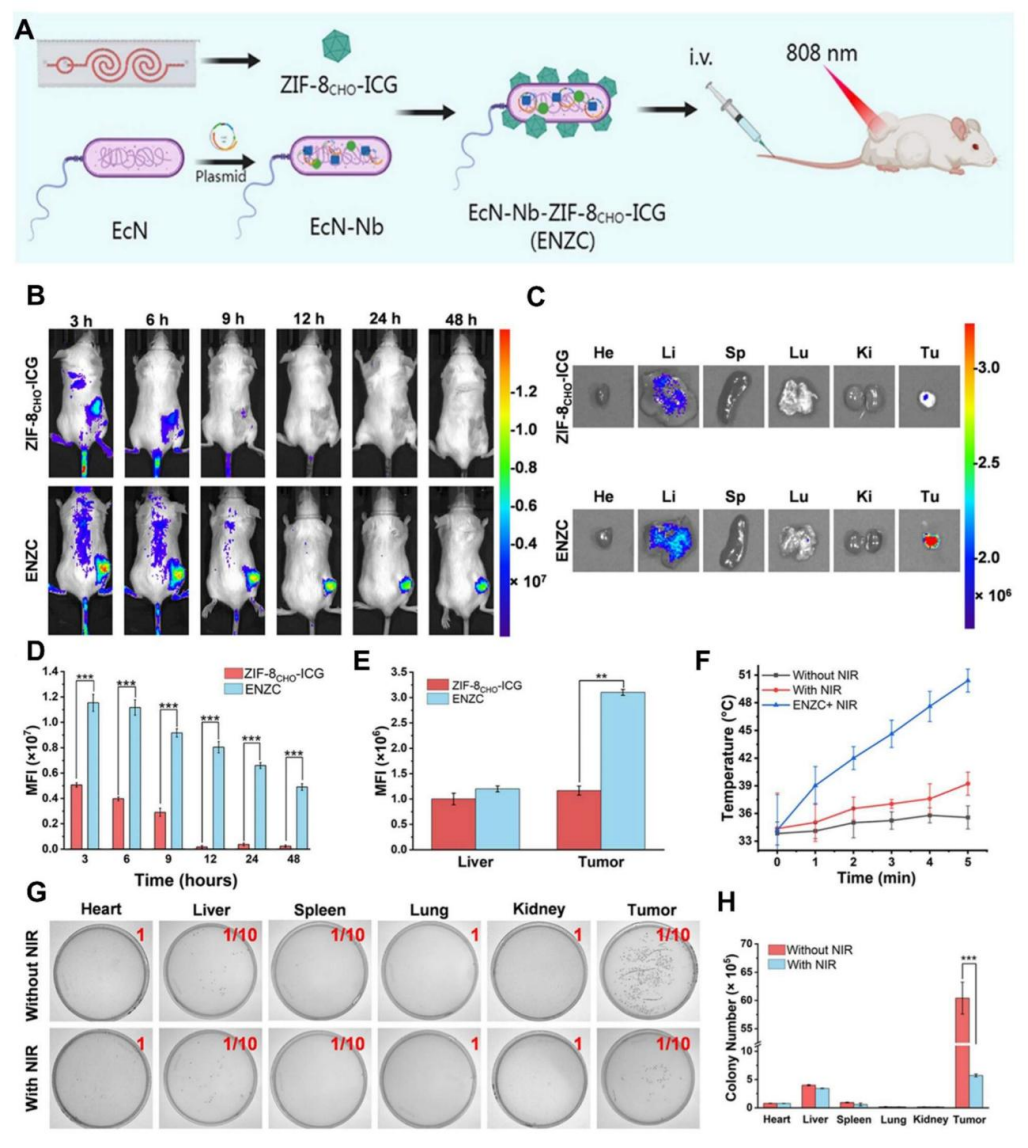
Targeted Delivery and Photothermal Evaluation of ICG-Loaded Engineered Bacteria in a 4T1-Luc Tumor Model **(A)** The photosensitizer indocyanine green (ICG) was encapsulated within ZIF-8CHO nanoparticles using a microfluidics-assisted method and subsequently conjugated to engineered *E. coli* Nissle 1917 via a Schiff base reaction. **(B)** *In vivo* fluorescence imaging using an IVIS system was performed at various time points post-injection, and the corresponding average fluorescence intensity was recorded. **(C)** *Ex vivo* imaging of tumor tissues was conducted to evaluate fluorescence distribution **(D)** At 3, 6, 9, 12, 24, and 48 hours following intravenous administration of either ZIF-8CHO-ICG or ENZC in 4T1-Luc tumor-bearing mice. **(E)** Fluorescence levels in liver and tumor tissues were also assessed 48 hours after injection. **(F)** The temperature change at the tumor site was monitored during laser irradiation (808 nm, 1.5 W/cm² for 5 minutes) following administration of 1 × 10⁷ CFU ENZC in tumor-bearing mice, demonstrating the photothermal effect. **(G)** Representative images and **(H)** quantitative analysis of bacterial colonies recovered from tumor homogenates after 24 hours of incubation on LB agar plates at 37 °C (n = 3 mice per group) confirmed tumor colonization by the engineered bacteria. Adapted with permission from ref. 167 Copyrights 2024, American Chemical Society.

Qin* et al*. 2024 demonstrated in his study a modified strain of *E. coli* Nissle 1917 (EcN) that co-expresses nanobodies against programmed death-ligand 1 (PD-L1) and CD9, a tetraspanin protein enriched in tumor-derived exosomes (TDEs). These engineered bacteria are encapsulated within zinc-based metal-organic frameworks (MOFs) loaded with indocyanine green (ICG), creating a composite system termed ENZC. Upon exposure to near-infrared (NIR) light, the ICG facilitates localized heating, triggering the lysis of the bacterial cells and subsequent release of the nanobodies directly within the tumor microenvironment. The dual-targeting strategy aims to neutralize PD-L1-mediated immune suppression and disrupt the function of TDEs, which are known to facilitate tumor progression and immune evasion. *In vivo* experiments demonstrated that this spatiotemporally controlled release system effectively remodels the tumor microenvironment by promoting the polarization of immunosuppressive to a pro-inflammatory phenotype **(Figure 17)**. Additionally, there was a notable increase in the infiltration and activation of cytotoxic T lymphocytes, leading to significant inhibition of tumor growth and metastasis 167.

### 5.4. Combination therapies

Combination therapies, particularly those combining engineered probiotics with chemotherapy or radiation, have gained attention as a promising approach in cancer treatment. These combination strategies aim to enhance the therapeutic effects of chemotherapy and radiation while mitigating their associated side effects. Probiotics, specifically engineered probiotics, offer a unique ability to target tumors locally, improve immune responses, and enhance the efficacy of traditional cancer treatments. By combining the immune-modulatory potential of probiotics with the cytotoxic effects of chemotherapy and radiation, researchers hope to create more effective, less toxic treatment regimens for cancer patients 168.

Chemotherapy and radiation therapy are standard treatments for many types of cancer. Chemotherapy uses cytotoxic drugs to kill rapidly dividing cancer cells, while radiation therapy utilizes high-energy radiation to destroy cancer cells or damage their DNA, leading to cell death. However, both therapies are not without their limitations 169. Chemotherapy can lead to systemic side effects, such as immunosuppression, nausea, and fatigue, due to its non-selective action on both cancerous and healthy cells. Similarly, radiation therapy can cause damage to normal tissues surrounding the tumor, resulting in adverse side effects, including skin burns, fatigue, and long-term risks of secondary cancers 170.

One of the main challenges in cancer therapy is overcoming the tumor microenvironment (TME), which is often immunosuppressive and limits the efficacy of treatments like chemotherapy and radiation. The TME consists of tumor cells, immune cells, extracellular matrix components, and blood vessels, and plays a critical role in tumor progression and therapy resistance. Tumors frequently recruit Tregs, myeloid-derived suppressor cells, and other immune cells that promote immune evasion and create a microenvironment that limits the effectiveness of therapies. In addition to the immune suppression, the TME can also exhibit altered blood supply, hypoxia, and nutrient deprivation, all of which contribute to treatment resistance 171. To overcome these challenges, the combination of engineered probiotics with chemotherapy or radiation therapy presents a novel approach. Probiotics, especially those that are engineered to express therapeutic proteins or enhance immune responses, can modulate the TME and enhance the therapeutic effects of traditional cancer treatments. Probiotics can target the tumor site directly, reducing systemic toxicity and increasing local drug delivery while simultaneously boosting immune responses and sensitizing the tumor to chemotherapy or radiation 172.

**Figure 18:**
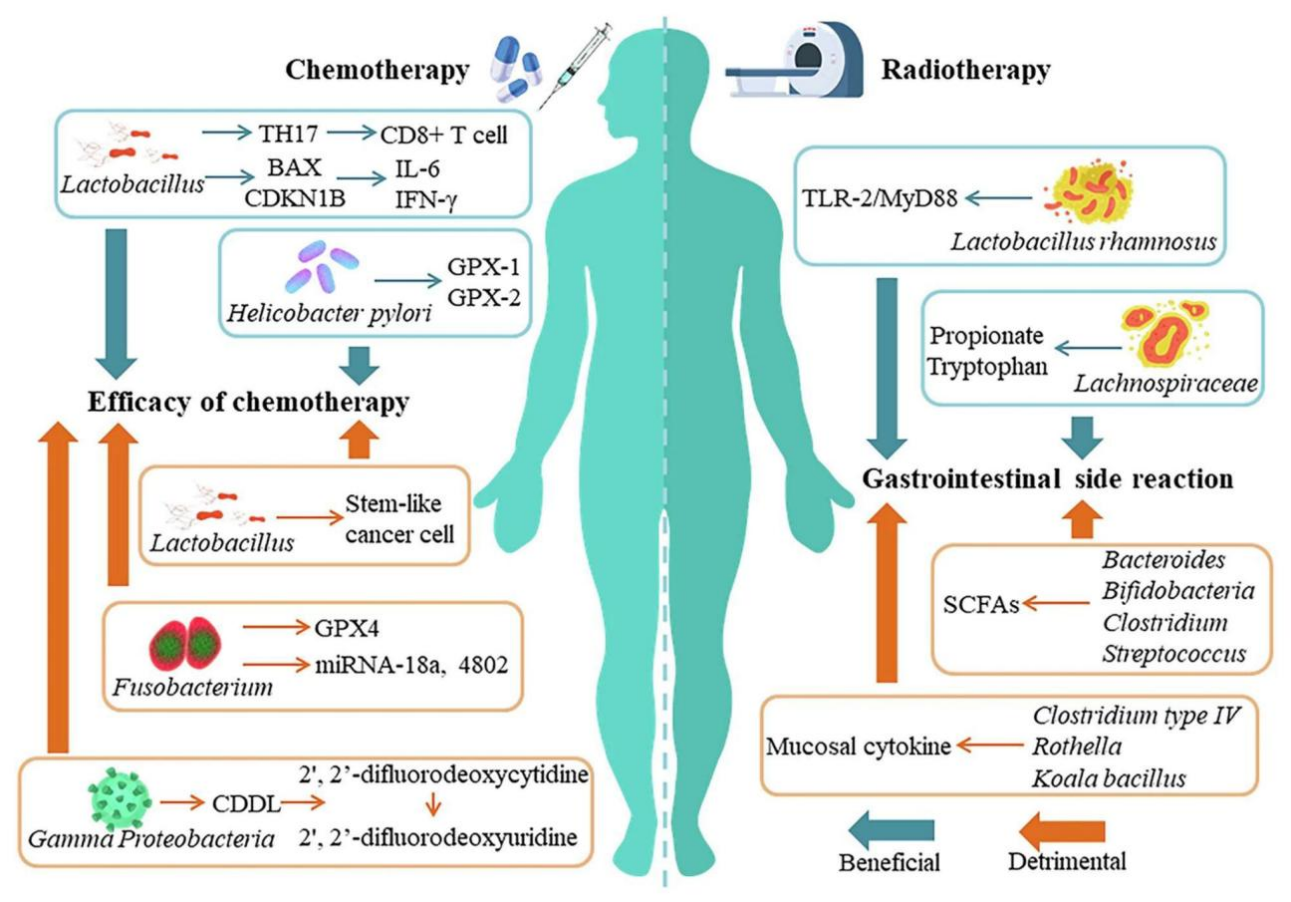
Apart from surgery, chemotherapy is the most essential cancer treatment. There is proof that the gut microbiota significantly affects the effectiveness and adverse effects of chemotherapy and radiation treatment. The effects of various common gut microbiotas on chemoradiotherapy are compared in this figure. The impacts of radiation are mostly focused on lowering complications, whereas the effects of chemotherapy are primarily focused on enhancing curative effect. Reused under Creative Commons Attribution License from ref. 173.

Sun* et al*. 2025 provide a comprehensive analysis of the pivotal role of the gut microbiota in cancer therapy, delineating its influence across multiple treatment modalities and exploring strategies to harness this microbiota-immune axis for therapeutic benefit. They first outline how dysbiosis contributes to tumor initiation and progression through mechanisms such as microbial metabolite secretion, mucosal inflammation, genotoxic pathways, and immune modulation highlighting both tumor-promoting and tumor-suppressive microbial actions. The authors emphasize that gut bacteria can significantly impact the efficacy and toxicity profiles of chemotherapy, radiotherapy, and immunotherapy, suggesting the microbiome as a predictive biomarker for treatment response. They then explore practical interventions to modulate the gut ecosystem, including fecal microbiota transplantation, probiotics, dietary adjustments, and targeted antibiotics, underscoring emerging evidence for these approaches in preclinical and early clinical settings. Finally, the review addresses the challenges associated with standardization, interindividual variability, and mechanistic complexity, while advocating for integrated approaches such as multi-omics profiling and controlled clinical trials to translate microbiome-guided strategies into precision anticancer therapies **(Figure 18)**. Overall, Sun* et al*. position the gut microbiota as a promising modulator and therapeutic target in cancer care, offering insights into mechanism-informed modulation for improved treatment outcomes 173.

In one study, *Lactobacillus* species were engineered to express IL-12, a cytokine known for its potent anti-tumor effects. IL-12 boosts the function of cytotoxic T lymphocytes (CTLs) and natural killer (NK) cells, both critical players in anti-tumor immune responses. In a breast cancer mouse model, engineered *Lactobacillus* was combined with chemotherapy drugs like cyclophosphamide to evaluate its therapeutic effects. Cyclophosphamide is a commonly used chemotherapy drug that induces immunosuppressive effects, such as the depletion of Tregs, which may limit the effectiveness of the treatment 174. The addition of IL-12-expressing *Lactobacillus* enhanced the cytotoxic effects of cyclophosphamide by promoting the activation of tumor-specific T cells and NK cells, resulting in reduced tumor growth and increased survival in the animal models 175. Similarly, engineered probiotics can be combined with radiation therapy to enhance the immune response and tumor control. Radiation therapy not only directly damages tumor cells but also induces the release of tumor-associated antigens (TAAs) into the surrounding microenvironment. These antigens can serve as targets for the immune system if properly presented. The TME often suppresses immune responses, preventing the immune system from effectively targeting tumor cells. However, engineered probiotics can enhance immune responses by delivering adjuvants or tumor antigens directly to the tumor site. This localized delivery can help overcome the immunosuppressive TME, thereby enhancing the effects of radiation therapy 176.

For example, *Lactococcus lactis* has been engineered to express tumor antigens and immune-stimulating molecules. When used in combination with radiation therapy, this engineered probiotic can deliver the tumor antigens to the immune system, stimulating a stronger immune response. In a preclinical study, engineered *Lactococcus lactis* expressing the tumor antigen MUC1 was combined with radiation therapy in a mouse model of breast cancer. The combination therapy resulted in a synergistic effect, where the probiotic-mediated delivery of tumor antigens enhanced the radiation-induced immune response, leading to significant tumor regression 177. This study demonstrated that probiotics could be used not only to enhance the immune system's ability to recognize and target tumor cells but also to improve the efficacy of radiation therapy by overcoming the immune suppression inherent in the TME.

Another important aspect of combining engineered probiotics with chemotherapy or radiation is the potential for improved tumor oxygenation and nutrient supply. Hypoxia, a common feature of solid tumors, can limit the effectiveness of radiation therapy. Tumors often develop abnormal blood vessels that lead to poorly oxygenated areas, making it difficult for radiation to effectively kill tumor cells. Engineered probiotics can be used to deliver therapeutic agents that enhance tumor vasculature or modulate the immune response in hypoxic regions 178. For example, *Bifidobacterium* species have been modified to produce vascular endothelial growth factor (VEGF), which supports the growth of new blood vessels and increases oxygen supply to tumors. When used alongside radiation therapy, this strategy may help address tumor hypoxia and improve the treatment's overall effectiveness 179. Probiotics also have the potential to reduce the side effects associated with chemotherapy and radiation therapy. Both treatments can lead to damage to the gastrointestinal tract, resulting in nausea, vomiting, and gut inflammation. Probiotics are known to have beneficial effects on gut health, including reducing inflammation and promoting the growth of beneficial gut microbiota. By improving gut health, engineered probiotics may help mitigate the gastrointestinal side effects of chemotherapy and radiation, making these treatments more tolerable for patients. In one study, *Lactobacillus rhamnosus* was shown to protect against chemotherapy-induced gut damage in mice by promoting the growth of beneficial gut bacteria and reducing inflammation. This protective effect could help enhance the overall quality of life for cancer patients undergoing chemotherapy or radiation therapy 180.

**Figure 19:**
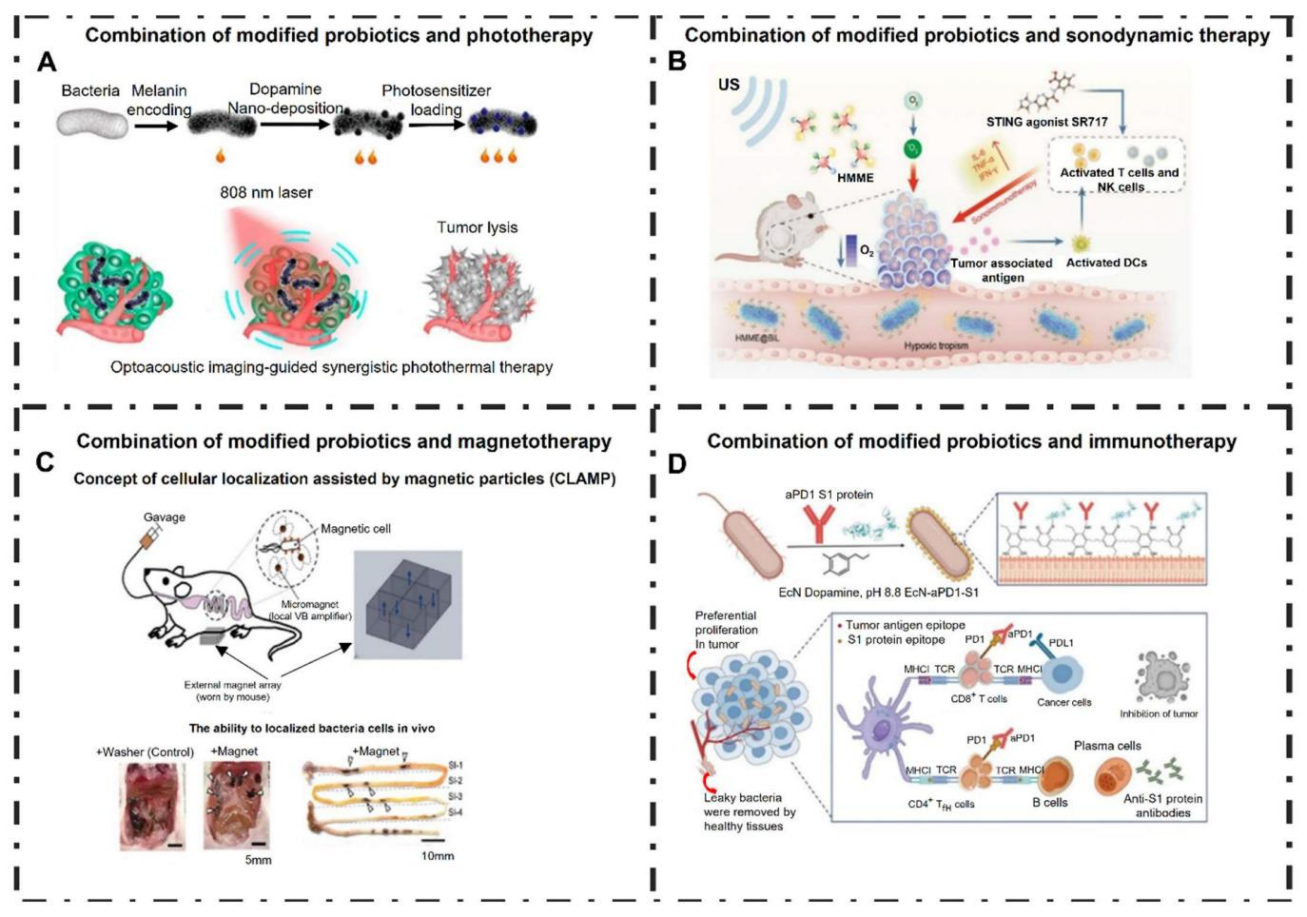
Integrative Approaches Combining Probiotics with Advanced Therapeutic Modalities **(A)** Synergistic photothermal therapy guided by optoacoustic imaging enhances precision and treatment efficacy. **(B)** Cancer immunotherapy utilizing probiotic-based synthetic immune therapeutics (SIT) in conjunction with a STING pathway agonist to stimulate robust antitumor responses. **(C)** Magnetic particle-assisted spatial manipulation of probiotics enables controlled localization within the gastrointestinal tract. **(D)** Functionalizing probiotic surfaces with a hybrid immunoreactive nanocoating to simultaneously activate antitumor and antiviral immune responses. Reused under Creative Commons Attribution License from ref. 181.

Zhao* et al*. 2025 stated in his study that probiotics hold great promise in the prevention and treatment of a wide range of diseases. However, their therapeutic potential is often limited by their sensitivity to the harsh *in vivo* environment, which results in significant loss of viability and diminished efficacy. To address these limitations, they developed modified probiotics, which offer enhanced stability and expanded functionality. These engineered probiotics are not only more resilient under physiological conditions but also possess novel attributes such as extended retention in the body and improved therapeutic performance. Through strategic modifications, probiotics was equipped with properties that enable them to better survive gastrointestinal transit, target specific disease sites, and interact more effectively with host systems. Furthermore, the integration of modified probiotics with other therapeutic approaches such as chemotherapy, immunotherapy, or diagnostic techniques has opened new pathways to improve clinical outcomes. This combination therapy paradigm significantly enhances the therapeutic effects of both probiotics and accompanying agents. They also highlight in-depth analysis of the progress made in probiotic-based combination therapies, including their applications in targeted drug delivery, disease treatment, diagnostic imaging, and biomarker detection **(Figure 19)**. These innovations not only improve the therapeutic index of probiotics but also broaden their application in precision medicine. The integration of advanced engineering strategies with probiotic therapy represents a promising frontier in developing multifunctional therapeutic platforms for complex diseases 181.

**Figure 20:**
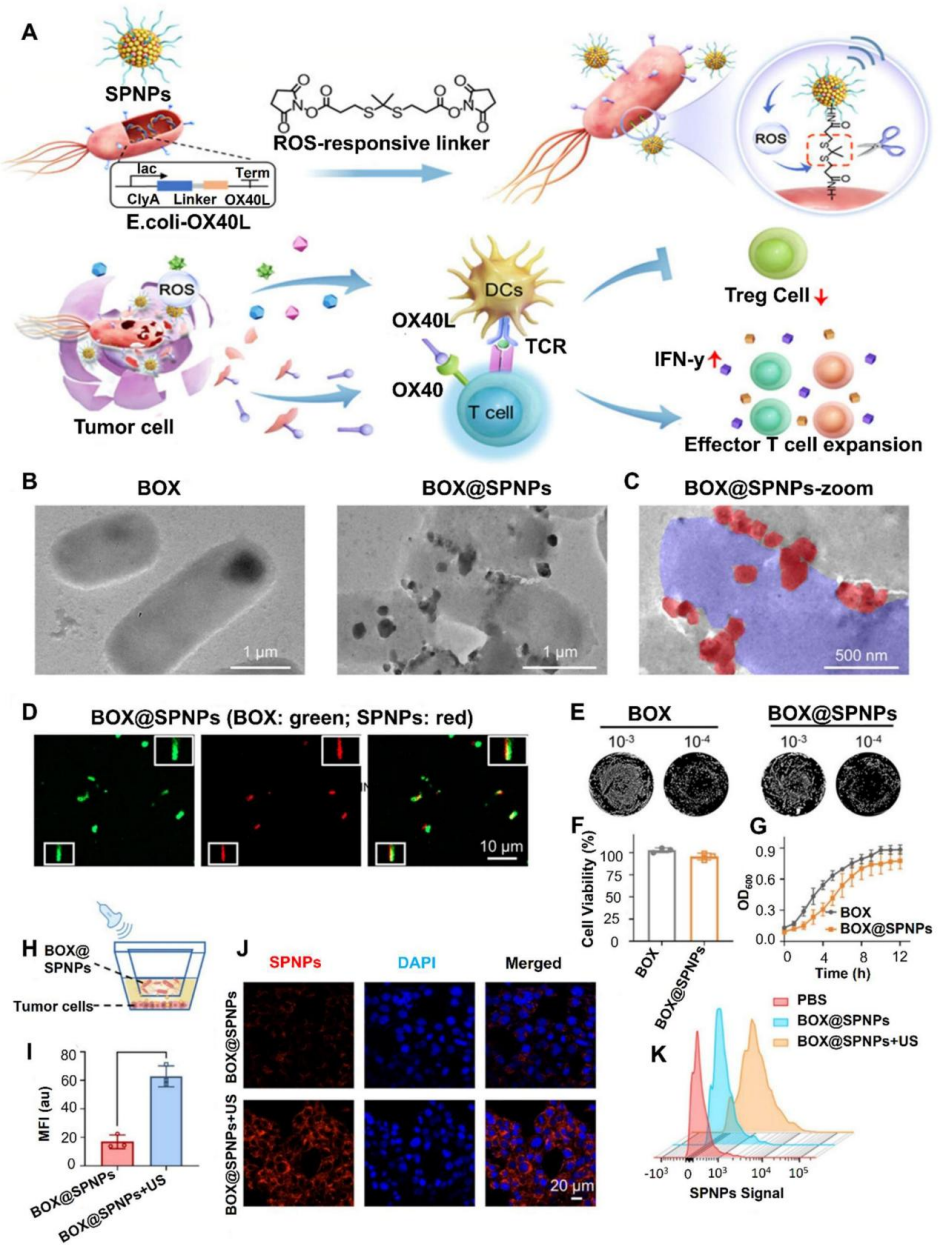
** (A)** Schematic Diagram of the Ultrasound-Powered Intelligent Biohybrid That Combines a Polymer Sonosensitizer with Genetically Engineered Bacteria to Initiate a Multidimensional Immune Cascade for Sturdy Tumor Immunotherapy. **(B)** BOX and BOX@SPNPs TEM images, and **(C)** a pseudocolor magnified picture of BOX@SPNPs. **(D)** BOX@SPNPs CLSM pictures, where BOX and SPNPs are indicated by green and red signals, respectively. **(E)** Images of BOX and BOX@SPNP bacterial colonies grown on LB agar plates. **(F)** Plate counting is used to quantify the corresponding bacterial viability. **(G)** BOX and BOX@SPNP growth curves at the same starting concentration. **(H)** A transwell model is used to evaluate the detachment of SPNPs from BOX@SPNPs during sono-irradiation. **(I)** Measurement of the bottom tumor cells' NP fluorescent signal. **(J)** CLSM pictures of the lower 4T1 cells treated with or without US irradiation after being incubated with BOX@SPNPs in the top chamber. **(K)** NP fluorescence signals from the bottom 4T1 cells incubated with BOX@SPNPs in the upper chamber, either with or without US treatment, as analyzed by flow cytometry. Adapted with permission from ref. 182 Copyrights 2025, American Chemical Society.

Liang *et al*. 2025 introduce a biohybrid living platform engineered to express the immune co-stimulatory ligand OX40L and paired with a polymer-based sonosensitizers to orchestrate a powerful ultrasound-triggered immune response against tumors. Under ultrasound irradiation, the sono-sensitive nanomaterials detach from the bacterial surface, enabling two synergistic actions: sonodynamic tumor cell damage and exposure of OX40L to activate T-cell immune pathways. This dual activation stimulates antigen release, enhances dendritic cell maturation, and robustly primes cytotoxic T cells, while concurrently counteracting immunosuppressive mechanisms within the tumor microenvironment. In preclinical cancer models, this strategy yielded substantial tumor suppression, durable immune memory, and prevention of metastasis and recurrence. By merging synthetic biology, sonodynamic therapy, and immune co-stimulation, this biohybrid represents a novel sono-immunotherapy modality that delivers controlled, vigorous, and long-lasting tumor immunity. The approach addresses key limitations of existing immunotherapies and presents a scalable framework for multi-dimensional activation of antitumor immunity **(Figure 20)** 182.

## 6. Imaging Guided Delivery of Probiotics Platform for Cancer Therapy

The integration of imaging technologies into probiotic-based therapeutic platforms represents a novel and promising direction in cancer treatment 183. Genetically engineered probiotics, including strains such as *E. coli* Nissle 1917, *Bifidobacterium*, and *Lactobacillus*, have shown an inherent capacity to localize within the hypoxic and immunosuppressive microenvironments characteristic of solid tumors. This ability to target tumors has been harnessed to engineer bacterial vectors that can deliver therapeutic payloads directly to the tumor site. However, challenges related to safety, precision, and control over bacterial distribution and persistence have limited their clinical application 184. To address these limitations, recent advances have focused on the development of imaging-guided delivery systems, enabling real-time tracking and monitoring of bacterial localization and therapeutic activity.

Imaging-guided delivery leverages various non-invasive imaging modalities to visualize and quantify the *in vivo* behavior of administered probiotics. Techniques like bioluminescence imaging (BLI), fluorescence imaging, magnetic resonance imaging (MRI), computed tomography (CT), and positron emission tomography (PET) have been used to improve the spatial and temporal tracking of bacterial distribution in the body 185. In preclinical models, BLI and fluorescence imaging are commonly used due to their high sensitivity and ease of use. Engineered probiotics expressing reporter genes such as *lux*, *gfp*, or luciferase produce detectable signals upon colonizing tumor tissues, facilitating longitudinal imaging of bacterial localization. Although these optical methods are limited by tissue penetration depth, they are invaluable in small animal studies for assessing colonization dynamics, clearance rates, and therapeutic payload expression 186.

For translational and clinical applications, deeper-penetrating imaging techniques like PET and MRI offer more robust solutions. In PET imaging, engineered bacteria can be designed to express enzymes such as thymidine kinase, which phosphorylate radiolabeled probes, allowing for accumulation of radioactivity at sites of bacterial presence. This approach has shown success in visualizing bacterial colonization in deep tissues 187. Similarly, MRI contrast can be enhanced by conjugating bacteria with magnetic nanoparticles such as superparamagnetic iron oxide (SPIO) or gadolinium-based agents, which generate signal alterations on MRI scans. These advanced imaging strategies facilitate not only localization of bacteria within tumors but also quantification of their abundance and assessment of treatment progression 188. In addition to imaging capabilities, these systems often incorporate therapeutic functionalities, resulting in multifunctional theranostic platforms. Nanoparticle-laden probiotics or bacteria conjugated with drug-loaded carriers have been developed to enable targeted therapy triggered by environmental cues or external stimuli such as light or ultrasound. These bacterially-driven hybrid systems, sometimes referred to as “bacteriobots,” allow for localized drug release within the tumor microenvironment, minimizing systemic toxicity and improving therapeutic efficacy. Furthermore, genetic circuits engineered into probiotic strains can be designed to conditionally express therapeutic molecules, such as cytokines or immune checkpoint inhibitors, in response to tumor-specific signals, providing a high degree of spatial and temporal control 189.

Another significant advantage of imaging-guided delivery is the ability to monitor therapeutic efficacy and safety in real-time. Through serial imaging, researchers can evaluate how effectively the probiotics are targeting the tumor, whether the therapeutic payload is being delivered as intended, and whether any off-target colonization is occurring. This monitoring capability enhances the safety profile of live bacterial therapeutics by enabling prompt detection of adverse events or deviations from expected biodistribution 190. Moreover, engineered kill switches or sensitivity genes can be incorporated to eliminate the bacteria post-treatment, with imaging used to confirm their clearance. Recent developments have also explored the use of multimodal imaging systems, combining different imaging technologies in a single platform to overcome the limitations of individual techniques. For instance, combining fluorescence imaging for initial localization with MRI for high-resolution anatomical mapping or PET for quantitative biodistribution analysis provides a comprehensive picture of probiotic behavior. Such integrated approaches enhance the reliability of tracking and can be used to tailor treatment protocols based on individual patient responses 191.

Despite the substantial progress in this field, several challenges remain to be addressed. One concern is the host immune response, which may limit the persistence and efficacy of engineered probiotics. Tumor heterogeneity also poses a challenge for consistent bacterial colonization and therapeutic effect 192. Moreover, regulatory considerations regarding the use of live bacteria in humans require thorough safety evaluations and standardized imaging protocols. Nevertheless, ongoing research and clinical trials are beginning to provide encouraging data, supporting the feasibility and potential of these systems for future application in oncology 193.

**Figure 21:**
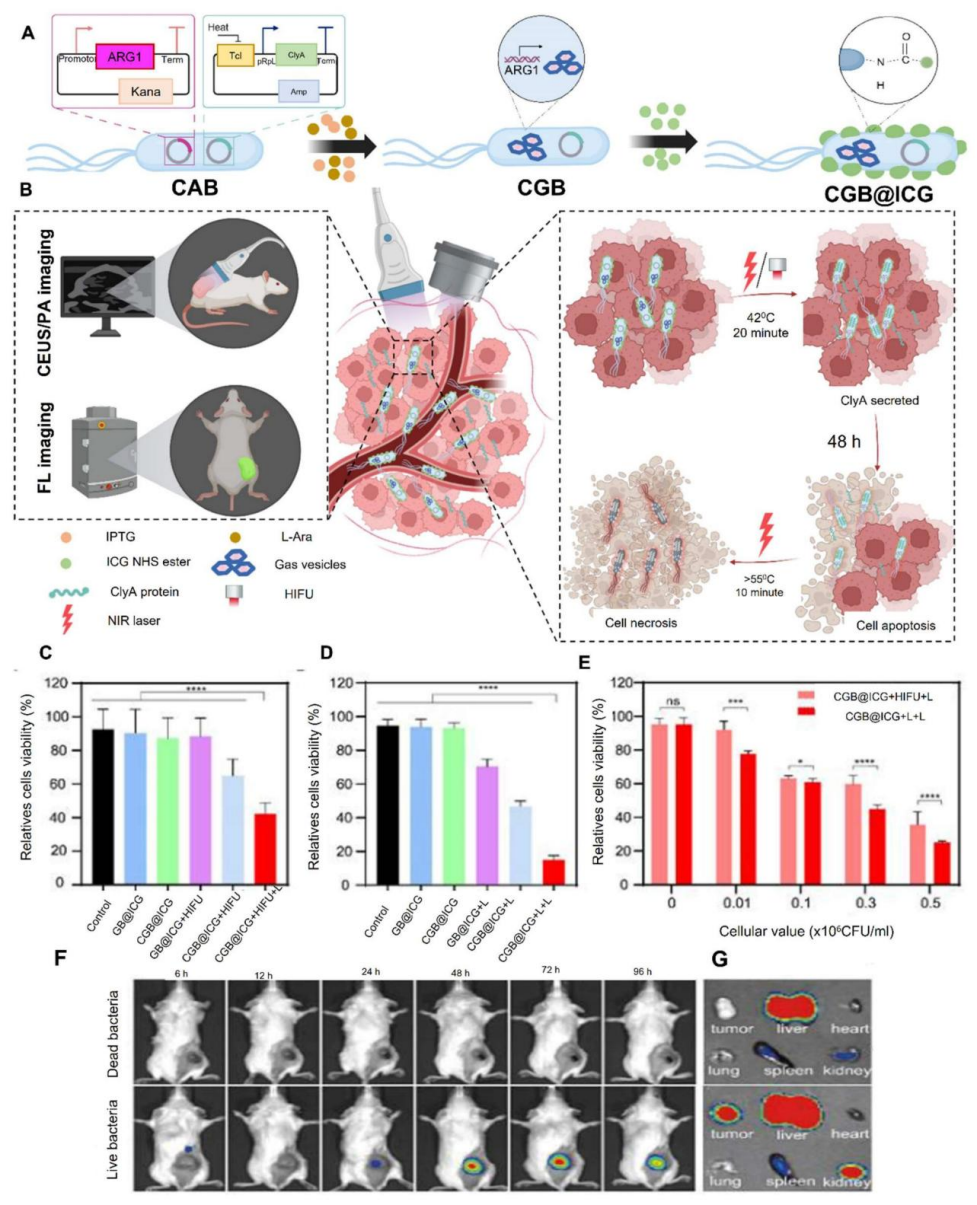
**(A)** The bacterial nanosystem CGB@ICG is created by conjugating indocyanine green (ICG) to bacteria that express ClyA-GVs. The production of gas vesicles (GVs) is induced by isopropyl β-D-1-thiogalactopyranoside (IPTG) and L-arabinose (L-Ara), while ICG is chemically linked to the bacterial surface through an amide bond. **(B)** Ultrasound (US), photoacoustic (PA), and fluorescence (FL) imaging techniques enable real-time tracking of CGB@ICG as it penetrates tumor cores. This facilitates controlled temperature regulation of ClyA secretion within the tumor, which promotes tumor cell death. **(C,D)** *In vitro* viability of 4T1 cancer cells was assessed after co-incubation with CGB@ICG or GB@ICG, with or without laser irradiation (808 nm, 55 °C for 10 minutes), and with or without induction of ClyA protein production (808 nm, 42 °C for 20 minutes) using high-intensity focused ultrasound (HIFU) or laser (L) treatment. **(E)** Relative differences in cell viability were measured at various cell concentrations following treatments with CGB@ICG combined with HIFU and laser (CGB@ICG+HIFU+L) or laser alone (CGB@ICG+L+L). **(F)** *In vivo* fluorescence imaging was conducted on tumor-bearing mice at different time points after intravenous injection of either heat-inactivated dead CGB@ICG or live CGB@ICG (2×10^8 colony-forming units, CFU; n=6 per group). **(G)** Fluorescence imaging of excised tissues was performed 48 hours post-injection. Reused under Creative Commons Attribution License from ref. 194.

Zeng* et al.* 2024 engineered a novel theranostic platform combining genetically programmed bacteria with photothermal capabilities, elegantly integrating multimodal imaging and remotely controlled therapeutic gene expression. They utilized an attenuated *E. coli* strain (CGB) harboring gas vesicle-based acoustic reporter genes (ARGs) and a thermo-inducible cytolysin A (ClyA) expression circuit. The bacterial surface was chemically conjugated with indocyanine green (ICG), yielding the hybrid CGB@ICG system. This construct facilitates contrast-enhanced ultrasound, photoacoustic, and near-infrared fluorescence imaging to pinpoint bacterial colonization in tumors, enabling real-time guidance for focused energy delivery. Upon localized high-intensity focused ultrasound (HIFU) or NIR laser irradiation, controlled hyperthermia induces ClyA expression and triggers photothermal ablation in tumor tissues. *In vitro* assays demonstrated a synergistic cytotoxic effect combining thermally triggered ClyA release and PTT achieving significantly lower 4T1 cancer cell viability than either modality alone. *In vivo*, 4T1-bearing mice treated with the CGB@ICG + HIFU + laser protocol exhibited rapid tumor regression, complete remission, and prolonged survival (> 45 days), whereas controls progressed normally. Histological analyses (H&E, TUNEL, Ki-67) confirmed robust apoptosis and decreased proliferation within treated tumors. Remarkably, CGB@ICG provides a closed-loop system: imaging guides energy delivery to induce therapeutic gene release at optimal timing, enhancing efficacy and biosafety **(Figure 21)**. The authors also show that residual bacteria can be thermally eradicated post-treatment to prevent systemic risks. This platform demonstrates precise spatiotemporal coordination of imaging, gene therapy, and PTT within tumors, highlighting a promising new paradigm in bacterial theranostics. Its success underscores the potential of integrating synthetic biology and imaging to improve targeted cancer therapy 194.

Imaging-guided delivery of probiotic platforms integrates diagnostic imaging with therapeutic delivery to enhance precision in cancer treatment. This approach allows real-time tracking of probiotic localization, colonization, and therapeutic activity within tumors. By combining imaging with engineered probiotics, it becomes possible to improve treatment accuracy, monitor therapeutic outcomes, and minimize off-target effects.

### 6.1 Fluorescence imaging

The convergence of microbial engineering and advanced imaging technologies has enabled the development of highly targeted strategies for cancer therapy. Among these, fluorescence imaging-guided delivery of probiotics presents a particularly attractive approach for real-time monitoring and precision treatment of tumors 195. Probiotics such as *E. coli* Nissle 1917, *Lactobacillus*, and *Bifidobacterium* species are being increasingly investigated as living vectors for the selective delivery of therapeutic payloads to tumor sites. Their natural tropism toward hypoxic, immunosuppressed tumor microenvironments makes them ideal candidates for such applications. However, a major challenge in clinical translation lies in the ability to monitor their biodistribution and activity *in vivo*. Fluorescence imaging offers a non-invasive, sensitive, and relatively inexpensive solution that allows researchers to track probiotic localization and function dynamically within living systems 196.

Fluorescence imaging involves the use of fluorescent probes or genetically encoded fluorescent proteins that emit detectable signals upon excitation by specific wavelengths of light 197. In the context of probiotic delivery, bacterial strains can be engineered to express fluorescent proteins such as green fluorescent protein (GFP), red fluorescent protein (RFP), or far-red variants like mCherry 198. The correspondents allow for the real-time visualization of bacterial populations after systemic or local administration. Unlike radiotracers used in PET or SPECT, fluorescent imaging agents do not involve ionizing radiation, making them more amenable to longitudinal studies in preclinical models. Furthermore, fluorescence imaging systems are widely accessible and can be integrated into small-animal imaging workflows without the need for complex infrastructure. One of the primary advantages of using fluorescence-guided delivery systems lies in their ability to confirm tumor targeting by the engineered probiotic vectors. After administration, fluorescent signals can be used to monitor the temporal kinetics of bacterial colonization within the tumor, as well as their retention and clearance over time 199. This is especially important in evaluating tumor specificity and minimizing off-target effects, which are critical concerns in the development of live bacterial therapeutics. In addition to localization, fluorescence imaging can provide semi-quantitative insights into the bacterial load and expression dynamics of therapeutic genes when coupled with inducible or constitutive fluorescent reporters.

Recent studies have demonstrated the feasibility of fluorescence-guided probiotic delivery systems in various tumor models. For instance, engineered *E. coli* strains expressing GFP have been used to visualize colonization patterns in subcutaneous and orthotopic tumor xenografts. These bacteria preferentially accumulated in necrotic and hypoxic tumor regions, validating their use as tumor-targeting vectors 200. Additionally, fluorescence imaging has been employed to monitor the co-delivery of therapeutic molecules, such as cytotoxic proteins, immune modulators, or enzymes that activate prodrugs. In such systems, dual-expression constructs enable the simultaneous production of a therapeutic agent and a fluorescent reporter, ensuring that imaging directly correlates with therapeutic function. To enhance the brightness and stability of fluorescence signals, researchers have also explored the use of synthetic fluorophores and nanoparticle-based labeling. Bacteria can be conjugated with fluorescent dyes or encapsulated in nanocarriers loaded with fluorescent agents 201. Quantum dots, near-infrared dyes, and other advanced fluorophores offer increased photostability and signal penetration depth compared to traditional fluorescent proteins. These strategies have allowed for improved visualization of bacterial behavior *in vivo*, particularly in deeper tissues or larger animal models where signal attenuation becomes a limiting factor 202.

Despite its numerous advantages, fluorescence imaging does have certain limitations that must be considered. One of the primary constraints is the limited tissue penetration of visible light, which restricts its application in deep-seated tumors or in human subjects 203. Light scattering and absorption by biological tissues can significantly reduce image resolution and sensitivity beyond a few millimeters of tissue depth. To address this, near-infrared (NIR) fluorescent probes, which operate in the 650-900 nm range, are increasingly being used. NIR imaging provides better tissue penetration and reduced background autofluorescence, enhancing the accuracy of bacterial tracking *in vivo* 204. Another consideration is the potential immunogenicity or metabolic burden of expressing foreign fluorescent proteins in probiotic strains. High-level expression of these proteins may interfere with bacterial viability or therapeutic function. Therefore, careful optimization of expression systems and use of low-burden reporter variants are essential. Additionally, the use of fluorescence imaging in clinical settings remains largely limited due to depth penetration issues and regulatory constraints regarding genetically modified organisms and imaging agents. Nevertheless, the utility of fluorescence imaging in preclinical studies remains indispensable for optimizing bacterial delivery strategies before transitioning to clinically compatible imaging modalities such as PET, MRI, or photoacoustic imaging 205.

Importantly, fluorescence imaging also facilitates the development and refinement of biosafety mechanisms within engineered probiotic systems. For example, programmable gene circuits incorporating both fluorescent reporters and safety switches (e.g., inducible kill genes or antibiotic sensitivity cassettes) allow for real-time confirmation of system behavior and rapid elimination of bacterial populations if needed 206. This integration of synthetic biology tools and imaging capabilities enhances the control and predictability of the probiotic delivery platform, which is essential for regulatory approval and clinical translation. Looking forward, the field is progressing toward the development of multifunctional platforms that combine fluorescence imaging with targeted therapy and responsive control. Probiotics can be designed to express therapeutic payloads in response to tumor-specific cues such as hypoxia, pH, or specific metabolites, with fluorescence reporters serving as real-time indicators of gene activation 207. Moreover, the emergence of multiplexed imaging technologies and AI-assisted image analysis will likely further enhance the resolution and interpretability of fluorescence-guided delivery systems.

Abdullah* et al*. 2023 engineered a multifunctional biocarrier by labeling heat-inactivated *Lactiplantibacillus plantarum* (HILP) with green-emitting carbon dots (CDs) and loading it with the red anticancer pigment prodigiosin (PG). The resulting CDs/HILP hybrid retained stable fluorescence in aqueous media for at least three months and exhibited distinct bicolor emission—green from CDs and red from prodigiosin enabling simultaneous tracking of both carrier and cargo. TEM and confocal microscopy confirmed effective labeling and formation of aggregated biostructures, while drug delivery studies in Caco-2 and A549 cancer cells demonstrated enhanced PG cytotoxicity, improved intracellular and nuclear localization, and increased late apoptosis compared to free prodigiosin. Moreover, the hybrid significantly inhibited cancer cell migration, and molecular docking suggested that prodigiosin interacts with key mitogenic regulators. Overall, this work highlights the potential of fluorescently labeled paraprobiotics as safe, sustainable, and traceable carriers for targeted anticancer therapeutics 208.

**Figure 22:**
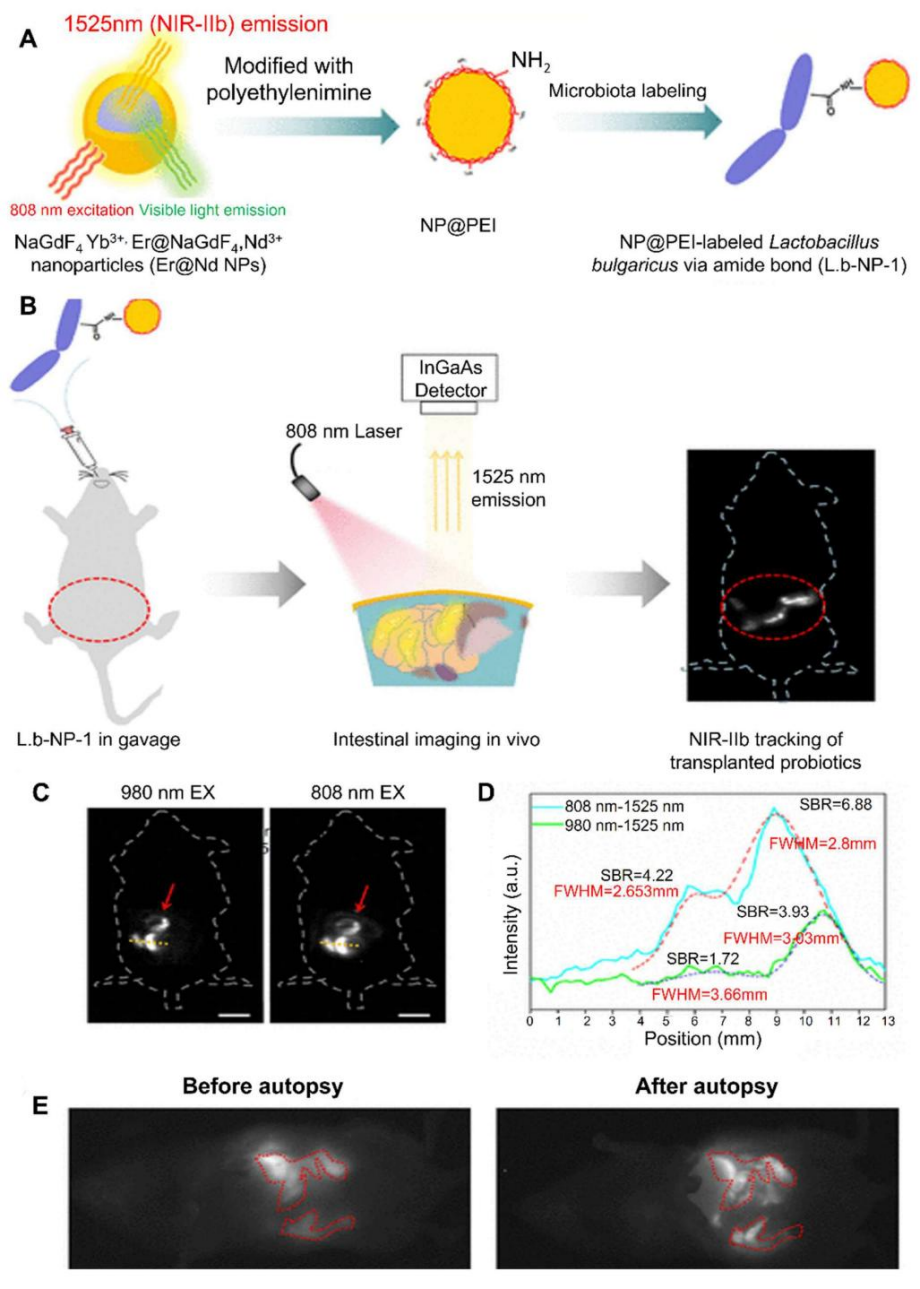
Probiotics in the Intestine Were Tracked Using IR-IIb Fluorescence Nanoparticles NaGdF4:Yb3+,Er3+@NaGdF4,Nd3+ (Er@Nd NPs). (A) L. bulgaricus Er@Nd NP Labeling (L.b-NP-1). (B) Monitoring of L. bulgaricus's NIR-IIb fluorescence following oral gavage of L.b-NP-1. (C) *In vivo* NIR-IIb fluorescence pictures following oral gavage of Er@Nd NPs into the mouse intestine. 10 mm is the scale bar. Analysis of the cross-sectional fluorescence intensity profile (CSFIP) using the yellow lines in (C) as a guide (D). (E) Before and after autopsy, the mouse intestine was imaged using 808 nm excitation-NIR-IIb emission. Adapted with permission from ref. 209 Copyrights 2023, American Chemical Society.

Ji* et al*. 2023 developed a novel imaging approach to track intestinal probiotics *in vivo* using second near-infrared window (NIR-IIb) fluorescence imaging. They synthesized core-shell lanthanide-based nanoparticles (NaGdF₄:Yb³⁺,Er³⁺@NaGdF₄:Nd³⁺) that emit in the 1500-1700 nm range and covalently conjugated them to *Lactobacillus bulgaricus* using EDC-NHS chemistry, ensuring stable attachment without compromising bacterial viability. This dual-mode system, combining NIR-IIb imaging and two-photon excitation microscopy, enabled deep tissue penetration and high-resolution visualization of bacterial distribution in the gastrointestinal tract of live mice. The real-time tracking demonstrated that labeled probiotics could be noninvasively monitored as they transited through the gut, with minimal cytotoxicity and preserved structural integrity** (Figure 22)**. This study represents a significant advancement in non-invasive fluorescence imaging technologies for microbiota research and offers valuable insights for future applications of engineered probiotics in targeted cancer theranostics 209.

### 6.2 Bioluminescence imaging

Bioluminescence imaging (BLI) has emerged as a powerful, non-invasive tool for tracking and monitoring biological processes in living organisms 210. In the context of cancer therapy, BLI has proven to be especially valuable for evaluating the behavior of live bacterial therapeutics, particularly probiotics engineered to deliver therapeutic agents selectively to tumor sites. The use of engineered probiotics such as *E. coli* Nissle 1917, *Bifidobacterium*, and *Lactobacillus* strains has gained increasing attention due to their ability to selectively colonize hypoxic, immunosuppressed tumor microenvironments 211. While this tumor-targeting capability offers a promising platform for localized therapy, ensuring the safety and precision of these approaches necessitates real-time, dynamic imaging technologies. BLI addresses this need by enabling researchers to non-invasively monitor probiotic biodistribution, viability, and gene expression in living hosts 212.

BLI works by detecting light produced from enzymatic reactions catalyzed by luciferases. These enzymes oxidize substrates (typically luciferins), resulting in the emission of photons that can be detected using highly sensitive charge-coupled device (CCD) cameras 213. In bacterial imaging, two main systems are commonly used: the firefly luciferase (*luc*) system, which requires exogenous administration of luciferin, and the bacterial *luxCDABE* operon, which encodes a self-sufficient luminescence system that does not require substrate administration. The latter, derived from species such as *Photorhabdus luminescens* or *Vibrio fischeri*, is particularly advantageous in bacterial tracking because it allows continuous and autonomous light emission from live bacterial cells. This enables researchers to longitudinally monitor the *in vivo* distribution and colonization kinetics of probiotic strains following systemic or local administration 214.

The natural preference of certain probiotic strains for the tumor microenvironment is largely attributed to the unique pathophysiological features of tumors, such as hypoxia, necrosis, low pH, and abnormal vasculature 215. These conditions inhibit immune clearance and create niches that are permissive for bacterial growth, particularly for facultative or obligate anaerobes. When administered systemically, these bacteria can escape phagocytic clearance, migrate to tumors, and proliferate preferentially within necrotic or poorly perfused regions. This property provides an ideal foundation for targeted therapy, but understanding and validating this process *in vivo* requires precise imaging 216. BLI enables researchers to visualize and quantify the extent of tumor colonization, monitor temporal dynamics of bacterial persistence, and correlate localization with therapeutic effect all in a live, non-terminal manner. One of the most important applications of BLI in probiotic delivery systems is the evaluation of tumor-targeting efficiency 217. In preclinical studies, engineered *E. coli* or *Bifidobacterium* strains expressing the *luxCDABE* operon are administered to tumor-bearing mice. As the bacteria colonize the tumor, the emitted bioluminescent signal can be captured using whole-body imaging systems. This allows for a clear spatiotemporal assessment of whether the bacteria are selectively localizing in the tumor versus healthy tissues. Studies have shown that such engineered bacteria can generate robust and stable luminescent signals within tumors as early as 24 hours post-administration, with signal intensity correlating with bacterial load. This provides a useful means of optimizing dosage, administration routes, and timing for maximum therapeutic impact 218.

Additionally, BLI is used to monitor the expression of therapeutic genes within engineered probiotic strains. Probiotic vectors are often designed to express therapeutic proteins such as cytokines (e.g., IL-2, IFN-γ), pro-apoptotic factors, or prodrug-converting enzymes under the control of tumor-specific or inducible promoters 219. By linking luciferase expression to these promoters, researchers can assess whether gene expression is occurring specifically within the tumor microenvironment. This ensures that therapeutic activity is localized, minimizing systemic toxicity and off-target effects. In dual-reporter systems, bioluminescence is used as a surrogate for therapeutic protein expression, allowing indirect but reliable assessment of functional gene delivery *in vivo*. BLI also facilitates the longitudinal monitoring of bacterial persistence and clearance. Understanding how long engineered probiotics remain viable in the host is crucial for balancing therapeutic efficacy with biosafety 220. Using BLI, researchers can track the presence and viability of bacteria over days or even weeks, observing whether the population expands, stabilizes, or declines over time. If bacterial overgrowth or unwanted dissemination is detected, kill-switch mechanisms (e.g., inducible lysis genes or antibiotic-sensitive modules) can be activated. BLI enables visualization of such clearance events, providing a robust safety evaluation framework prior to clinical translation 221.

Moreover, BLI is instrumental in studying the host immune response and its impact on bacterial colonization. Immune clearance mechanisms, including phagocytosis and antibody-mediated killing, can influence the persistence of probiotic vectors. By correlating BLI signal changes with immunological parameters (e.g., cytokine levels, immune cell infiltration), researchers can better understand the interaction between engineered bacteria and the host immune system. This, in turn, supports the design of immunomodulatory strategies that enhance colonization while preventing systemic inflammation or septic responses 222.

While BLI provides numerous advantages, it is not without limitations. The most notable constraint is the limited tissue penetration of light, which restricts the technique primarily to small animal models. Photons emitted by luciferase reactions are easily absorbed and scattered by biological tissues, reducing image resolution and accuracy in deep-seated organs or larger animals. 223. As a result, BLI is not directly translatable to clinical imaging in humans. Nevertheless, its value in preclinical development is immense, as it allows researchers to screen probiotic constructs, evaluate safety and efficacy, and optimize delivery parameters before transitioning to more clinically relevant imaging modalities such as PET or MRI. In recent years, efforts have been made to combine BLI with other imaging modalities to overcome its depth limitations and enhance resolution. For example, dual-reporter constructs that include both luciferase and fluorescent or PET-compatible reporters have been used for multi-modal imaging. Such hybrid systems allow researchers to harness the strengths of different imaging approaches for instance, using BLI for high-sensitivity initial tracking, and PET for deep-tissue visualization and quantification. These developments reflect a broader trend toward multifunctional, multi-modal platforms in the field of theranostics 224.

Another promising avenue is the integration of BLI with synthetic biology tools to create responsive, programmable probiotic therapies. Genetic circuits can be engineered to activate luciferase expression in response to environmental cues such as hypoxia, specific metabolites, or quorum-sensing signals. This allows researchers to use BLI not just as a localization tool, but also as a biosensor that reports on tumor-specific conditions or therapeutic gene activation. In such systems, light emission becomes a readout for therapeutic engagement, enabling more precise and personalized treatment planning 225.

Du* et al*. 2024 developed an innovative bacteria-driven delivery system that enhances sonodynamic therapy (SDT) against triple-negative breast cancer by harnessing immunogenic cell death (ICD). They synthesized PLGA nanodroplets co-loaded with the sonosensitizer hematoporphyrin monomethyl ether (HMME) and perfluoro-n-pentane (PFP), termed HPNDs, and covalently attached these to the surface of *E. coli* Nissle 1917 using carbodiimde chemistry. The engineered EcN acted as a micromotor, actively homing to tumor sites and boosting local HMME accumulation compared to passive nanosystem delivery. Upon ultrasound-triggered cavitation of PFP, HMME was released to generate reactive oxygen species and potentiate SDT, resulting in apoptosis and robust ICD *in vitro* and *in vivo*. Treated tumors showed significantly suppressed growth and metastasis under ultrasound irradiation, demonstrating that the bacteria-mediated system markedly improves tumor targeting and ICD efficacy in SDT 226.

**Figure 23:**
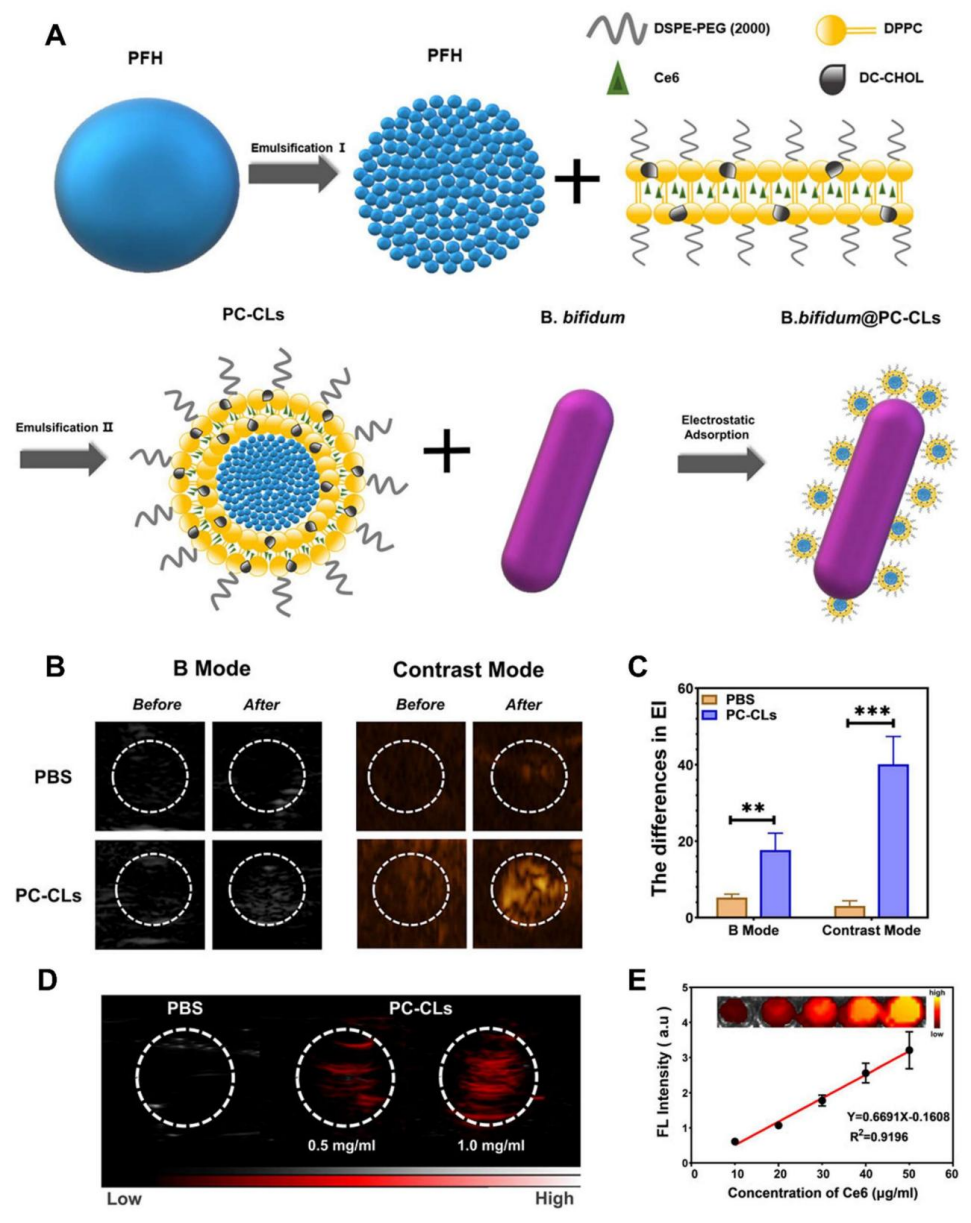
** (A)** Schematic of the B. bifidum@PC-CLs synthesis process. **(B)** TUNEL, Ki67, and HE staining pictures of the tumor's targeted region following treatment. **(C)** Ultrasound pictures and quantitative evaluation of EI in B and contrast modes before to and following PFUS for the PC-CLs group (n = 3) and the PBS group. **(D)** PBS and PC-CL PA pictures. **(E)** A FL picture showing the linear correlation between the concentration and the FL signal value for various Ce6 concentrations. Reused under Creative Commons Attribution License from ref. 227.

Ou *et al.* 2025 introduced a pioneering bio-targeted synergist platform denoted B. bifidum@ PC-CLs that integrates *Bifidobacterium bifidum* with multifunctional nanoparticles (PC-CLs) carrying chlorin e6 (Ce6) and perfluorohexane (PFH), designed to enhance pulse focused ultrasound (PFUS) cancer treatment through improved reactive oxygen species (ROS) generation and multimodal imaging guidance. The PC-CLs demonstrated strong capabilities in ROS production, sonodynamic therapy (SDT) enhancement, and multimodal visualization. By leveraging the natural tumor-targeting properties of *B. bifidum*, achieved via electrostatic adsorption to tumor-homing bacteria, the system effectively delivered and retained PC-CLs at tumor sites in murine 4T1 models. In combination with PFUS, the hybrid system generated a cascade cavitation effect, amplified ROS production upon Ce6 activation, and produced significant tumor suppression both *in vitro* and *in vivo* **(Figure 23)**. This study highlights a powerful, synergistic theranostic strategy that couples bacterial targeting with PFUS-triggered ROS therapy and real-time imaging offering a novel platform for precise, efficient, and safe tumor ablation 227.

### 6.3 PET and SPECT

Positron emission tomography (PET) and single-photon emission computed tomography (SPECT) are among the most sensitive and clinically established molecular imaging modalities currently available. Their integration into probiotic-based cancer therapy platforms has significantly advanced the field by enabling quantitative, non-invasive, and deep-tissue imaging of engineered bacterial therapeutics 228. In contrast to optical imaging techniques such as fluorescence or bioluminescence, which suffer from limited tissue penetration, PET and SPECT offer high sensitivity and full-body imaging capability in small animals and humans. These advantages make them especially attractive for translating probiotic delivery strategies from preclinical models to clinical applications 229.

PET and SPECT imaging are both nuclear medicine techniques that detect the distribution of radiolabeled tracers administered to a living subject. PET uses positron-emitting isotopes such as ^18F, ^64Cu, or ^124I, while SPECT relies on gamma-emitting isotopes like ^99mTc or ^111In. The radiolabeled probes emit signals that are captured by detectors to reconstruct high-resolution, three-dimensional images of tracer distribution throughout the body 230. When applied to probiotic imaging, these tracers can be used to detect engineered bacterial strains that express specific enzymes, receptors, or transporters capable of selectively binding or metabolizing the radiotracers. This allows for visualization of bacterial colonization at tumor sites with excellent sensitivity, specificity, and spatial resolution 231. One of the primary strategies for PET and SPECT imaging of engineered probiotics is reporter gene imaging. In this approach, bacterial strains are genetically modified to express a reporter gene that interacts with a radiolabeled substrate. A common example is the expression of herpes simplex virus thymidine kinase (HSV1-tk), an enzyme that phosphorylates radiolabeled nucleoside analogs such as [^18F] FHBG or [^124I] FIAU. Once phosphorylated, these substrates become trapped inside the bacterial cell, allowing PET imaging to visualize areas of bacterial accumulation. This method has been successfully used to monitor the localization and persistence of engineered *E. coli* or *Salmonella* strains in tumor-bearing mice, with signal intensity correlating to bacterial load 232.

Alternatively, bacterial strains can be labeled directly using radio metals that bind to the bacterial surface or are taken up through natural transport systems. For instance, isotopes like ^64Cu or ^111In can be chelated using ligands and conjugated to the surface of bacteria or their outer membrane vesicles. When injected into animals, the radiolabeled bacteria migrate to tumors, and the emitted PET or SPECT signal allows researchers to track their movement and accumulation in real time 233. Although this method provides immediate visualization, it does not distinguish between live and dead bacteria and lacks long-term tracking capabilities compared to reporter gene systems. Another advanced approach involves the use of metabolic labeling, in which bacteria are engineered to metabolize or uptake specific radiolabeled compounds. For example, some probiotic strains can be modified to express bacterial sodium iodide symporters (NIS), which transport radioactive iodine isotopes such as ^124I or ^99mTcO_4^- into the cell. Upon administration of the radiotracer, the engineered bacteria accumulate the isotope, making them visible through PET or SPECT. This method offers high specificity and enables repeated imaging sessions to evaluate colonization dynamics over time.

The application of PET and SPECT imaging in probiotic therapy is particularly valuable for assessing tumor-targeting efficiency and systemic biodistribution. After administration, engineered probiotic strains can be visualized as they migrate through the host's vasculature and accumulate within tumors. Imaging at different time points allows for the analysis of bacterial homing kinetics, persistence at the tumor site, and potential off-target colonization in healthy tissues. This information is critical for evaluating therapeutic safety, adjusting dosing strategies, and optimizing delivery routes. Studies have shown that probiotic strains such as *Bifidobacterium longum* and *E. coli* Nissle 1917 engineered with PET-compatible reporter genes preferentially accumulate in tumor tissues while being minimally present in other organs, demonstrating the high selectivity of these systems 234.

PET and SPECT imaging also enable the real-time monitoring of therapeutic gene expression in response to tumor-specific cues or environmental signals. By using inducible promoters linked to reporter genes or combining therapeutic gene expression with a radiotracer-responsive module, researchers can correlate therapeutic activation with imaging signals. This capability is crucial for validating the on-demand release of anticancer agents such as cytokines, apoptosis inducers, or prodrug-converting enzymes. The ability to visualize both bacterial presence and therapeutic activity provides a comprehensive view of treatment progression and supports adaptive therapeutic planning 235. In addition to live imaging, PET and SPECT data can be quantitatively analyzed to assess pharmacokinetics and therapeutic efficacy. The standardized uptake value (SUV), a common quantitative metric in nuclear imaging, provides a numerical estimate of tracer accumulation in specific regions, enabling objective comparisons between experimental groups or treatment conditions. This quantitative nature of PET and SPECT imaging is particularly valuable in preclinical and clinical studies, where precise measurement of therapeutic vector behavior is essential for regulatory approval and clinical decision-making 236.

Despite their advantages, PET and SPECT imaging techniques have certain limitations. One challenge is the radiation exposure associated with the use of radioactive isotopes, which may limit repeated imaging sessions in clinical settings. In preclinical research, however, the radiation doses are well within acceptable limits for small animals. Another consideration is the complexity and cost of radiotracer production and imaging infrastructure, which may limit accessibility in certain research environments 237. Moreover, the stability of radiolabeling and the potential for tracer detachment or degradation must be carefully evaluated to avoid misleading results. Efforts are underway to improve the specificity, safety, and versatility of PET and SPECT imaging in probiotic platforms. One direction involves the development of dual-modality systems, where bacteria are engineered to express both nuclear and optical or magnetic reporter genes. This allows cross-validation of imaging data and integration of high-resolution anatomical imaging (e.g., MRI) with highly sensitive molecular imaging (e.g., PET). Additionally, researchers are exploring clinically translatable reporter systems that avoid viral genes or exotic enzymes, aiming for compatibility with human immune systems and regulatory standards 238.

Chen et al. (2021) engineered the probiotic strain *E. coli* Nissle 1917 to biosynthesize and deliver 5-aminolevulinic acid (5-ALA), a photosensitizer precursor used in photodynamic therapy (PDT), to colorectal tumors. By co-expressing the hemA and hemL genes on a low-copy plasmid and inhibiting the downstream pathway with levulinic acid, the modified strain accumulated up to 300 mg/L of 5-ALA in culture. When cocultured with colorectal cancer cells, the engineered bacteria effectively produced protoporphyrin IX (PpIX) upon 5-ALA conversion and, following irradiation at 630 nm, induced selective cytotoxicity towards cancer cells with minimal impact on healthy tissues. In a mouse xenograft model, oral or local administration of the engineered *E. coli* resulted in significant tumor regression under PDT, without observed systemic toxicity. The study highlights the feasibility of using probiotic bacteria as living therapeutic agents for targeted tumor treatment via metabolic production of photosensitizers 239.

**Figure 24:**
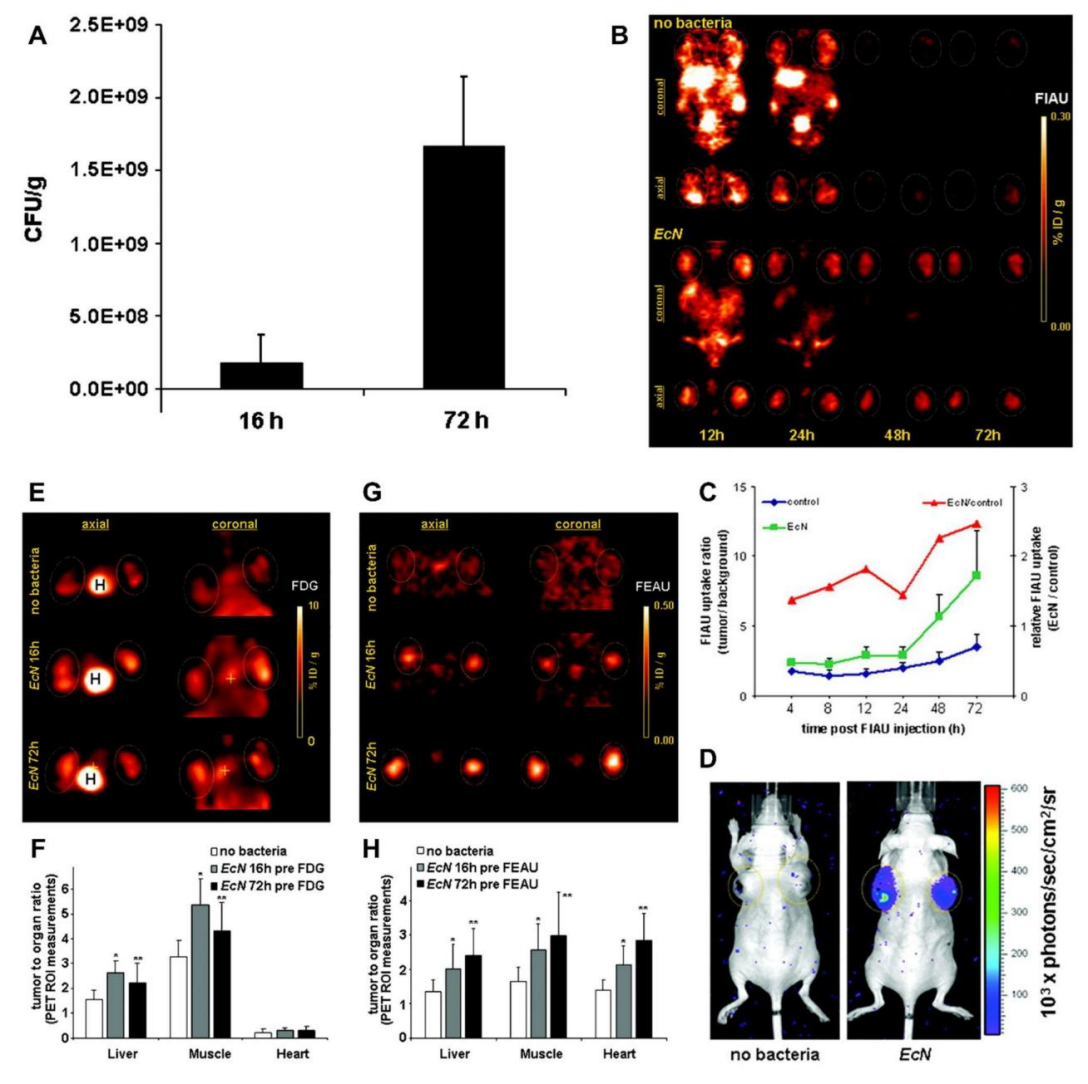
**(A)** Colonization of tumors by *E. coli* Nissle (EcN) was assessed at 16 and 72 hours after bacterial injection. **(B)** Representative axial and coronal microPET images using [^124I] FIAU tracer show EcN-treated and untreated (control) 4T1 xenograft-bearing mice at different time points (12, 24, 48, and 72 hours post-injection). **(C)** Quantification of [^124I] FIAU uptake in tumors compared to background was performed using region-of-interest (ROI) measurements; each group included six tumors. **(D)** Bioluminescence imaging of the same animals was captured four hours after administration of L-arabinose, which induces luciferase expression in EcN × pBR322DEST PBAD-DUAL-term bacteria. Tumors are indicated in the images. **(E)** Typical axial and coronal microPET images using [^18F] FDG tracer from a 4T1 xenograft-bearing rat treated with EcN (at 16 and 72 hours) and a control animal are shown. **(F)** Tumor-to-organ ratios of [^18F] FDG uptake in 4T1 tumor-bearing mice were calculated from ROI measurements of the microPET images. **(G)** Normal axial and coronal microPET images using [^18F] FEAU tracer from an EcN-treated (16 and 72 hours) 4T1 xenograft-bearing rat and an untreated control are presented. **(H)** Tumor-to-organ ratios of [^18F] FEAU uptake were determined from ROI analysis in 4T1 tumor-bearing mice. Reused under Creative Commons Attribution License from ref. 240.

Brader et al. (2008) investigated the tumor-targeting and imaging capabilities of the probiotic strain *E. coli* Nissle 1917 (EcN) using both PET and optical methods. This study leveraged EcN's endogenous thymidine kinase activity to phosphorylate radiolabeled pyrimidine analogues [^18F]-FEAU in particular enabling high-contrast PET imaging of bacterial accumulation in 4T1 breast tumor-bearing mice. Compared to [^18F]-FDG, [^18F]-FEAU demonstrated superior tumor-to-background contrast, and radiotracer uptake correlated linearly with viable EcN colony counts in tumors. To complement nuclear imaging, EcN was engineered with an l-arabinose-inducible luciferase reporter to enable bioluminescence tracking of bacterial colonization, offering a low-cost and sensitive optical modality **(Figure 24)**. The results confirmed that EcN effectively homes to and proliferates within tumors, and can be noninvasively monitored *in vivo* without genetic manipulation of imaging genes highlighting the feasibility of translating EcN-based PET imaging into clinical applications for detecting solid tumors 240.

### 6.4. MRI and CT

The application of non-invasive imaging techniques has significantly advanced the development of probiotic-based delivery systems for cancer therapy. While optical methods like bioluminescence and fluorescence imaging have been instrumental in early preclinical investigations, their limited tissue penetration restricts their utility in clinical settings 241. In contrast, magnetic resonance imaging (MRI) and computed tomography (CT) offer high-resolution anatomical imaging with deep tissue penetration and have well-established roles in clinical diagnostics. These modalities are increasingly being explored in the context of imaging-guided probiotic delivery systems, offering new opportunities for monitoring bacterial behavior, tumor colonization, and therapeutic outcomes *in vivo* with greater spatial accuracy and clinical relevance 242.

MRI is a powerful imaging tool that provides detailed soft tissue contrast without the use of ionizing radiation. It relies on the behavior of hydrogen nuclei in a magnetic field and their response to radiofrequency pulses, generating images based on the differences in relaxation times of water and fat in tissues 243. This makes MRI particularly suitable for imaging tumors, which often exhibit altered vascularity, cellularity, and interstitial fluid content. In the context of bacterial therapy, MRI has been employed by labeling probiotic strains with contrast agents to enable visualization of bacterial biodistribution and colonization in tumors. Most commonly, superparamagnetic iron oxide nanoparticles (SPIONs) are used to label bacteria *ex vivo* or through genetic modification to enable biosynthesis of magnetosome-like particles. These SPIONs induce local magnetic field inhomogeneities, resulting in T2 signal reduction (hypointensity) on MR images, thereby highlighting the regions of bacterial accumulation 244. Several studies have demonstrated the feasibility of MRI-based tracking of probiotic and commensal bacteria in animal tumor models. For example, *E. coli* Nissle 1917 or *Lactobacillus* strains labeled with SPIONs have been administered intravenously or intratumorally in mice, and subsequent T2-weighted MR imaging successfully visualized their localization within the tumor mass. This approach offers high anatomical resolution and enables real-time monitoring of the spatial distribution of bacteria, which is essential for assessing tumor-targeting efficiency and minimizing off-target colonization 245. Moreover, since MRI is capable of detecting changes in the tumor microenvironment, such as edema or necrosis, it can also provide indirect evidence of therapeutic efficacy following probiotic-mediated treatment.

In addition to external labeling, genetic engineering of bacteria to produce endogenous MRI contrast agents has also been explored. Some bacteria, particularly magnetotactic strains, naturally synthesize magnetosomes membrane-bound magnetic crystals composed of iron oxide that can serve as intrinsic MRI contrast agents. While these strains are not typically used in probiotic therapy due to biosafety concerns, synthetic biology has made it possible to introduce magnetosome gene clusters into probiotic strains, potentially enabling genetically encoded MRI contrast without the need for exogenous agents. This strategy, although still in early development, opens new possibilities for long-term, non-invasive tracking of viable bacterial populations *in vivo* 246.

CT on the other hand, is a radiographic technique that provides high-resolution, three-dimensional images of internal structures based on differences in X-ray attenuation. Although CT is most effective for imaging bone and air-filled structures, its use in soft tissue imaging can be enhanced through the application of radiodense contrast agents such as iodine or barium 247. In the context of probiotic therapy, CT has primarily been used as a complementary modality to MRI or PET, offering detailed anatomical reference to support molecular or functional imaging. However, emerging strategies have also sought to enable direct visualization of bacteria via CT by conjugating them with high-attenuation contrast agents. Bacteria can be labeled with gold nanoparticles (AuNPs) or iodinated compounds, both of which exhibit high X-ray attenuation and provide strong contrast on CT scans 248. Gold nanoparticles are particularly attractive due to their biocompatibility, tunable surface chemistry, and high contrast efficiency. Engineered bacteria coated with AuNPs or encapsulated within gold-loaded carriers can be tracked using CT after systemic or local administration. Upon tumor colonization, these labeled probiotics produce hyperdense signals that can be distinguished from surrounding tissue, allowing precise localization of bacterial populations. Additionally, the presence of these nanoparticles enables theranostic capabilities, as gold nanoparticles can also be used for photothermal therapy when exposed to near-infrared light, offering combined imaging and treatment functionalities 249.

The integration of MRI and CT with therapeutic evaluation has opened new avenues for understanding the spatial and temporal dynamics of probiotic-based cancer therapy. By enabling visualization of both bacterial localization and tumor morphology, these modalities allow researchers to correlate bacterial presence with changes in tumor size, vascularization, or necrosis. This is especially important in assessing treatment responses over time, helping to determine whether the bacterial vectors are effectively delivering their therapeutic payload and whether tumors are responding appropriately. In some cases, contrast-enhanced MRI or CT can also be used to evaluate the integrity of blood vessels or the permeability of the tumor microenvironment, which may influence bacterial infiltration and therapeutic delivery 250.

Despite the promise of MRI and CT in this field, there are some limitations. The primary challenge in using MRI for bacterial imaging is the need for sufficient concentrations of labeled bacteria to produce detectable contrast, particularly when using T2-weighted imaging. Moreover, signal voids induced by SPIONs can sometimes be nonspecific and confused with other hypointense structures such as calcifications or hemorrhages. On the other hand, CT imaging involves exposure to ionizing radiation, which may limit its use in repeated imaging sessions, particularly in sensitive or immunocompromised subjects 251. Additionally, direct labeling with gold or iodine-based agents can affect bacterial viability or function if not optimized carefully. To overcome these limitations, multimodal imaging platforms are being developed that combine MRI or CT with complementary techniques such as PET, SPECT, or fluorescence imaging. For instance, bacteria labeled with both SPIONs and radiotracers can be simultaneously visualized using MRI and PET, leveraging the anatomical precision of MRI with the sensitivity of PET. Similarly, gold nanoparticles can be engineered to carry fluorescent dyes, enabling dual CT-fluorescence imaging. These hybrid approaches enhance the accuracy of bacterial tracking and therapeutic monitoring, while also enabling validation of findings across different imaging modalities 252.

Hill *et al.* 2011 engineered probiotic *E. coli* Nissle 1917 to overexpress bacterial ferritin homologs as endogenous MRI reporter genes and evaluated their ability to enhance contrast in tumor-colonized tissue. Among the three ferritin variants tested bacterioferritin, ferritin-like protein, and Dps protein bacterioferritin produced the most pronounced T₂-weighted MRI signal *in vitro* and *in vivo*, despite similar iron accumulation across variants. Using an inducible promoter activated within tumors, the study demonstrated that bacterioferritin-expressing EcN colonized 4T1 breast cancer xenografts in mice, resulting in clear MRI contrast changes between colonized and control tumors. Site-directed mutagenesis confirmed that heme coordination was not required for contrast generation. This pioneering work highlights the feasibility of tracking live, tumor-targeting probiotic vectors using intrinsic iron-storage mechanisms, and establishes a foundational strategy for integrating bacterial MRI reporters into theranostic applications 253.

**Figure 25:**
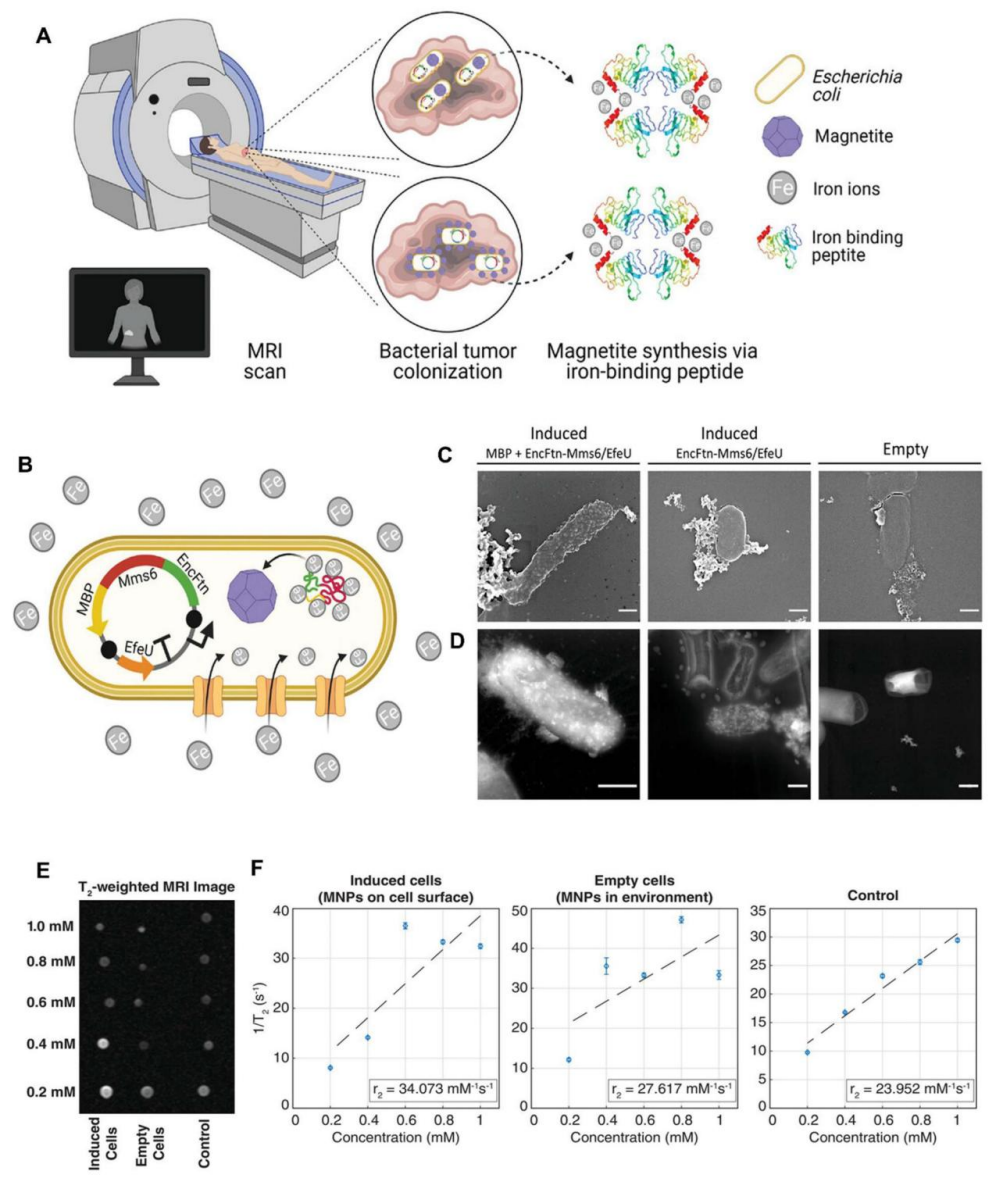
(A) An illustration of modified cells that accumulate magnetite and have relevant genetic circuits for tumor diagnostics. (B) ICMAB is made using ferroxidase, MBP, and iron transporter proteins in addition to the Mms6 peptide. Images of the generated magnetites inside the cell are shown in (C) SEM and (D) TEM. There are 500 nm scale bars. (E) Measurements of ECMAB r2 relaxivity. This is an example of a 3T T2-weighted MRI picture with TR/TE = 2000/60 ms. Iron concentrations of 0.2, 0.4, 0.6, 0.8, and 1 mM Fe are used to create MNPs on the cell surface, MNPs in the environment, and the control group. (F) Inverse of T2 for MNPs on the cell surface, MNPs in the environment, and the control group consisting only of ferrous and ferric ions as a function of iron content (0.2-1 mM Fe). Twelve distinct TE values ranging from 10 to 1000 ms are used to estimate the T2 values. The ROI's mean and standard deviation at each concentration are shown by the blue error bars, while linear fits are shown by the dashed lines. Adapted with permissions from ref. 254 Copyrights 2022, Wiley.

Yavuz *et al.* 2022 engineered *E. coli* to accumulate magnetite nanocrystals, transforming the bacteria into living MRI contrast agents with potential theranostic utility. Using synthetic genetic circuits, they co-expressed the magnetosome-associated peptide Mms6 along with ferroxidase, iron transporter proteins, and an autotransporter to either produce magnetite intracellularly or display Mms6 on the cell surface. MRI measurements revealed enhanced paramagnetic properties in engineered strains compared to controls, showing strong T₂-weighted contrast attributable to magnetite deposition. This magnetite-loading strategy enables noninvasive *in vivo* imaging via MRI and also sets the stage for magnetothermal therapy or targeted drug delivery **(Figure 25)**. By integrating biological magnetite synthesis with modular bacterial platforms, this work provides a versatile blueprint for combining diagnostics and therapeutic functionalities highlighting a novel direction for bacterial-based cancer theranostics 254.

## 7. Challenges and Limitations

### 7.1 Biosafety and containment risks

The use of engineered probiotics as live therapeutic agents raises serious concerns regarding biosafety. Key risks include uncontrolled replication, horizontal gene transfer, and systemic infection, especially in immunocompromised cancer patients. While synthetic biology tools such as inducible kill switches, auxotrophic mutations, and antibiotic-sensitivity modules have been developed to improve safety, these systems may not always function reliably *in vivo*. Environmental fluctuations within the host can disrupt circuit function, potentially leading to bacterial escape or prolonged colonization in unintended tissues. Robust biocontainment strategies that are responsive, fail-safe, and clinically validated are still urgently needed for broader clinical application 255.

### 7.2 Tumor microenvironment heterogeneity

One of the defining features of solid tumors is their heterogeneous microenvironment, which presents a significant barrier to consistent probiotic colonization and therapeutic delivery. Hypoxia, acidic pH, poor vascular perfusion, immune infiltration, and stromal density vary widely within and between tumors, influencing bacterial access and survival. Even though certain probiotics preferentially colonize hypoxic and necrotic tumor cores, they may be excluded from well-perfused or immune-active regions. This heterogeneity can result in uneven therapeutic distribution and limit overall efficacy. Moreover, variability between tumor types and patients poses challenges to standardizing and predicting probiotic behavior 256.

### 7.3 Host immune response to engineered probiotics

Although some probiotics are commensal organisms, engineered strains expressing foreign or immunostimulatory proteins may trigger undesired host immune responses. These responses can include innate clearance by macrophages and neutrophils, antibody-mediated opsonization, and adaptive immune memory formation. As a result, therapeutic bacteria may be rapidly neutralized before achieving sufficient colonization or payload delivery. Additionally, repeated dosing may be hampered by immune priming, reducing long-term efficacy. Strategies such as immune cloaking, immune-silent expression systems, and encapsulation are being explored to prolong bacterial persistence while minimizing inflammation, but remain in early stages of development 257.

### 7.4 Limited control of *in vivo* gene expression

Precise regulation of therapeutic gene expression is critical for safety and efficacy. However, achieving reliable gene control *in vivo* is challenging due to the dynamic and unpredictable nature of the host environment. Tumor-specific promoters, quorum-sensing systems, and metabolite-responsive circuits have been employed to restrict gene expression to tumor sites, but these often suffer from leaky expression or inconsistent activation. Environmental fluctuations such as pH shifts, oxygen gradients, and host immune signals may interfere with circuit stability. As a result, off-target expression may occur, leading to toxicity, while underexpression can render the therapy ineffective. More robust, tunable, and orthogonal gene regulation systems are needed 258.

### 7.5 Challenges in delivery and colonization efficiency

The route of probiotic administration plays a major role in determining therapeutic outcomes. Oral delivery, while non-invasive, subjects bacteria to gastric acid and bile salts, significantly reducing viability before reaching the tumor. Intravenous injection faces clearance by the liver, spleen, and immune system, while intratumoral injection is limited to accessible tumors and does not address metastases. Furthermore, factors such as bacterial motility, chemotaxis, adhesion, and biofilm formation can affect colonization efficiency. Enhancing these traits without compromising safety is a delicate balance that requires precise genetic engineering and validation 259.

### 7.6 Variability in preclinical models and lack of standardization

Most studies on engineered probiotics have been conducted in murine models, which often do not fully recapitulate human tumor biology, microbiota composition, or immune responses. This leads to a translational gap between preclinical findings and human clinical outcomes. Additionally, the field currently lacks standardized protocols for evaluating bacterial colonization, therapeutic gene expression, and immune response, making it difficult to compare results across studies. The development of standardized, reproducible, and clinically relevant models and metrics is essential for advancing this field 260.

### 7.7 Regulatory and manufacturing hurdles

Engineered probiotics fall into the category of live biotherapeutic products (LBPs), which are subject to stringent regulatory frameworks. Compliance with good manufacturing practices (GMP), consistent strain engineering, scalability, stability, and product sterility must be demonstrated prior to clinical use. Moreover, genetically modified organisms (GMOs) face heightened regulatory scrutiny, particularly concerning environmental release and biosafety. Different countries have varying regulations regarding the use of GMOs in humans, creating further complexity in clinical development and international collaboration 261.

### 7.8 Uncertain long-term safety and ecological impact

The long-term effects of introducing engineered probiotics into the human body and potentially into the environment are not yet fully understood. Persistent colonization, unintended disruption of the gut microbiota (dysbiosis), or the horizontal transfer of engineered genes to native microbes could have unforeseen consequences. The release of engineered strains into wastewater or natural ecosystems also raises concerns regarding ecological balance and biosafety. Long-term follow-up studies, robust environmental risk assessments, and post-treatment surveillance strategies will be essential to ensure safety beyond the treatment window 262.

Despite their therapeutic potential, the use of bacteria for the theranostics of brain tumors faces several challenges and limitations. The blood-brain barrier poses a major obstacle, restricting bacterial penetration and distribution within brain tissue. Safety concerns, including risks of uncontrolled infection, systemic toxicity, and immune overactivation, further limit clinical application.

## 8. Future Directions and Opportunities

### 8.1 Bacterial gas vesicles for drug delivery

Bacterial gas vesicles, protein-based nanostructures naturally found in aquatic microbes, have recently emerged as innovative tools for drug delivery and imaging. Their biocompatibility, ability to carry payloads, and acoustic contrast properties make them attractive for ultrasound-guided therapy 263. Engineered gas vesicles can be used to encapsulate therapeutic molecules and deliver them to tumor sites under imaging guidance, offering a non-invasive and trackable method for precise drug release. Future efforts may focus on genetically modifying these vesicles for responsive or stimuli-triggered therapeutic delivery, particularly in hypoxic tumor regions 264.

### 8.2 Bacteria-loaded nanoparticles for cancer theranostics

Combining the tumor-targeting ability of bacteria with the functional versatility of nanoparticles has created hybrid systems with great promise in cancer theranostics. These biohybrids can simultaneously enable imaging (via fluorescence, MRI, or photoacoustics) and therapeutic action (via chemotherapy, photothermal, or photodynamic effects). Leveraging bacterial tropism toward hypoxic or necrotic tumor regions, nanoparticles can be transported deep into tumors for localized treatment. Future directions include optimizing surface functionalization, minimizing immunogenicity, and integrating multi-modal imaging for real-time therapy tracking 265.

### 8.3 Bacteria to treat cancers in the gut and intestinal tract

Given their natural colonization of the gastrointestinal tract, probiotic and engineered bacterial strains present significant potential for the treatment of gut-related cancers, such as colorectal cancer. These bacteria can be programmed to release therapeutic payloads (e.g., cytokines, toxins, or checkpoint inhibitors) in response to tumor-specific signals 266. Their localized activity reduces systemic toxicity and allows for microbiota modulation, potentially reversing tumor-promoting inflammation. Future work may explore combining bacterial therapy with immunotherapy or dietary interventions for enhanced outcomes.

### 8.4 Bacteria to penetrate the blood-brain barrier (BBB) for glioma treatment

One of the most promising frontiers in bacterial cancer therapy is the use of engineered strains capable of penetrating the blood-brain barrier (BBB) to target brain tumors such as gliomas. Certain facultative anaerobes and genetically modified bacteria exhibit the ability to cross the BBB and selectively colonize brain tumors, enabling *in situ* drug production or immune activation 267. Future research should focus on refining bacterial tropism, ensuring biosafety, and integrating responsive gene circuits to achieve controlled therapy within the brain's delicate environment.

### 8.5 Fluorescence/CT/PET/MRI-guided bacteria treatment

Image-guided bacterial therapy represents a powerful approach to monitor and control bacterial localization, proliferation, and therapeutic function in real time. By engineering bacteria or their payloads to express contrast agents or reporter genes, researchers can use modalities like fluorescence imaging, CT, PET, and MRI to noninvasively track treatment progress 268. Such multimodal imaging enables precise spatiotemporal control over therapeutic delivery and response evaluation. The integration of image-guided systems with synthetic biology tools opens avenues for smart, adaptive treatment regimens.

### 8.6 Bacterial outer membrane vesicles (OMVs) for drug delivery

Bacterial outer membrane vesicles (OMVs) are nanosized proteoliposomes naturally secreted by Gram-negative bacteria and have shown great promise as drug carriers. OMVs can be engineered to carry small molecules, proteins, or nucleic acids, and their Immunostimulatory properties can enhance anticancer immune responses. Due to their nanoscale size and modifiability, OMVs can be targeted to tumors and loaded with imaging probes for theranostic applications 269. Future development could involve engineering OMVs to evade immune detection and deliver cargo in a stimuli-responsive, tumor-selective manner.

### 8.6 Precision genetic engineering and synthetic biology

Future efforts should prioritize the development of more refined genetic tools for engineering probiotics. Techniques such as CRISPR-Cas systems, recombinase-based switches, and synthetic gene circuits can enable programmable control over probiotic behavior, ensuring precise spatial and temporal activation of therapeutic functions within tumor tissues 270. These advancements will enhance safety and reduce unintended effects.

### 8.7 Overcoming regulatory and biosafety challenges

To advance toward clinical application, issues of biosafety, biocontainment, and environmental risk must be addressed. Developing kill-switch mechanisms, auxotrophic strains, and standard regulatory frameworks will be essential for clinical trials and eventual approval 271.

### 8.8 Scalable manufacturing and standardization

Reliable, large-scale production of engineered probiotics under GMP conditions will be critical. Establishing robust protocols for formulation, storage, and delivery, alongside validated *in vivo* models for efficacy testing, is vital for clinical translation 272.

### 8.9 Interdisciplinary collaboration and clinical translation

The future of probiotic-based cancer immunotherapy depends on strong interdisciplinary collaborations between microbiologists, immunologists, synthetic biologists, oncologists, and regulatory experts 273. Translational research and well-designed clinical trials will determine the pace and success of these emerging therapeutics.

### 8.10 AI-driven synthetic biology design

AI-driven synthetic biology design is emerging as a powerful approach to advance cancer treatment by enabling the rapid and precise engineering of probiotic systems. By integrating machine learning algorithms with synthetic biology tools, researchers can predict and optimize genetic circuits, metabolic pathways, and therapeutic payloads for enhanced tumor targeting and immune modulation. This approach accelerates the development of tailored probiotic therapies, improves efficiency, and reduces experimental trial-and-error, paving the way for more effective and personalized cancer treatments.

### 8.11 Spatial omics and with single-cell sequencing

Spatial omics combined with single-cell sequencing offers a powerful approach to unravel the interaction mechanisms between probiotics and tumors. Spatial omics enables mapping of molecular and cellular processes within the tumor microenvironment, preserving spatial context, while single-cell sequencing provides high-resolution insights into gene expression and cell heterogeneity. Together, these technologies can reveal how probiotics colonize tumors, influence immune responses, and interact with specific cell populations.

### 8.12 Combination of probiotics with different therapies

Multimodal combined treatment strategies, such as integrating probiotics with photothermal, photodynamic, or ultrasound-driven therapies, represent a promising advancement in enhanced tumor immunotherapy. Probiotics can actively target and colonize tumor sites while modulating the immune microenvironment, creating an optimal platform for combination treatments. When paired with photothermal or photodynamic approaches, they can improve local tumor destruction and immune activation, while ultrasound-driven systems can enhance therapeutic delivery and precision. Such synergistic strategies offer improved tumor eradication, reduced side effects, and a more effective immune response compared to single-modality treatments.

The safety and biosafety of engineered probiotics remain a critical area for further exploration. As live organisms are being genetically modified, concerns regarding their stability, potential for horizontal gene transfer, and unintended immune responses must be carefully addressed 274. To this end, researchers are working on strategies to make engineered probiotics safer, such as incorporating "kill-switch" mechanisms that allow the probiotics to self-destruct or become inactivated once they have performed their therapeutic role. These safety systems are crucial for ensuring that the probiotics do not persist in the body or spread genetic material that could lead to unintended consequences 275. Another promising approach is the development of synthetic probiotics that are engineered with highly controlled genetic circuits, which can allow for precise regulation of gene expression in response to external stimuli or changes in the TME. By ensuring that therapeutic genes are only expressed when and where they are needed, these systems could significantly reduce the risk of adverse effects.

Looking ahead, collaboration between microbiologists, oncologists, immunologists, and bioengineers will be crucial in realizing the full potential of engineered probiotics in cancer therapy. Interdisciplinary research will not only accelerate the discovery of new probiotic strains with enhanced therapeutic properties but also foster the development of innovative delivery systems, biosafety mechanisms, and combination therapies. This will pave the way for more effective, personalized, and safer probiotic-based cancer treatments. Additionally, as regulatory frameworks evolve to accommodate novel therapeutic modalities, it is expected that engineered probiotics will become an integral part of the cancer treatment landscape, providing patients with new avenues for combating this complex and heterogeneous disease.

## 9. Conclusion

The use of engineered probiotics in cancer therapy represents a revolutionary and highly promising approach that integrates microbiology, immunology, and synthetic biology to enhance the treatment of cancer. By harnessing the natural properties of probiotics, including their ability to modulate immune responses and deliver therapeutic agents directly to tumors, this innovative strategy offers a new avenue for overcoming some of the limitations associated with traditional cancer treatments. These probiotics can be engineered to target specific tumor microenvironments, enhance immune activation, and deliver therapeutic payloads such as cytokines, immune checkpoint inhibitors, or tumor antigens, potentially improving the efficacy of chemotherapy, radiation, and immunotherapy. However, significant challenges remain in the development and clinical application of engineered probiotics. Safety concerns, including the potential for unintended immune responses or horizontal gene transfer, must be addressed through the incorporation of advanced biosafety mechanisms and stringent regulatory frameworks. Additionally, the complexities of tumor heterogeneity and the need for precise targeting within the dynamic tumor microenvironment make it essential to continue refining probiotic delivery systems and therapeutic modalities. The use of synthetic biology tools, personalized treatment strategies, and combination therapies holds great promise for enhancing the effectiveness of engineered probiotics, enabling more tailored and targeted interventions that can improve patient outcomes. As the field progresses, further research into the microbiome's role in cancer therapy, the optimization of engineered probiotic strains, and the integration of these therapies into clinical settings will be crucial. Collaborative efforts across disciplines, including microbiology, oncology, and bioengineering, will accelerate the development of safe, effective, and personalized probiotic-based cancer therapies. Despite the challenges, the potential for engineered probiotics to complement and enhance current cancer treatments offers hope for more effective, less toxic, and personalized cancer therapies, ultimately improving the quality of life and survival rates for cancer patients worldwide.

## Figures and Tables

**Scheme 1:**
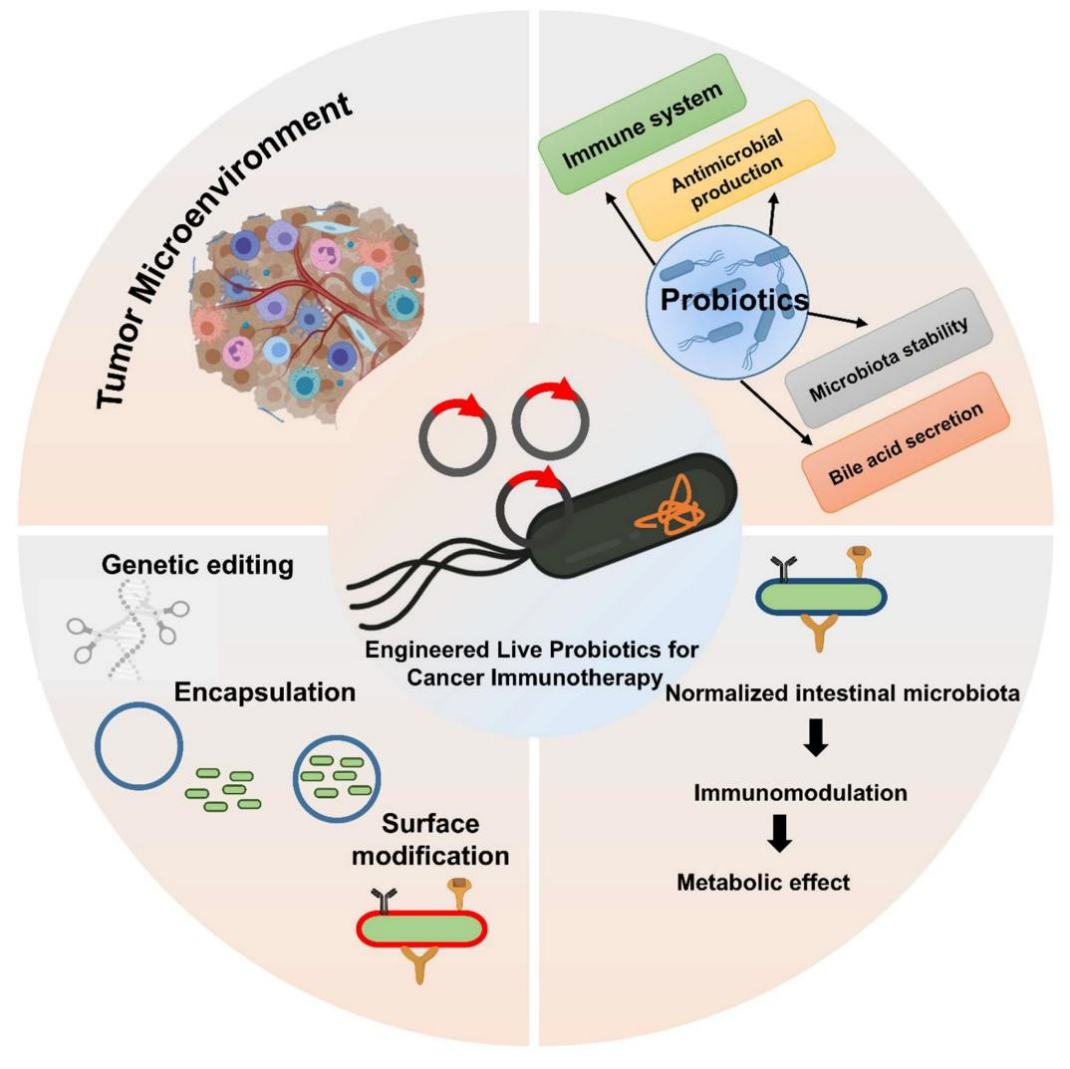
This illustration shows genetic engineering and usage of genetically modified probiotics.

**Figure 26:**
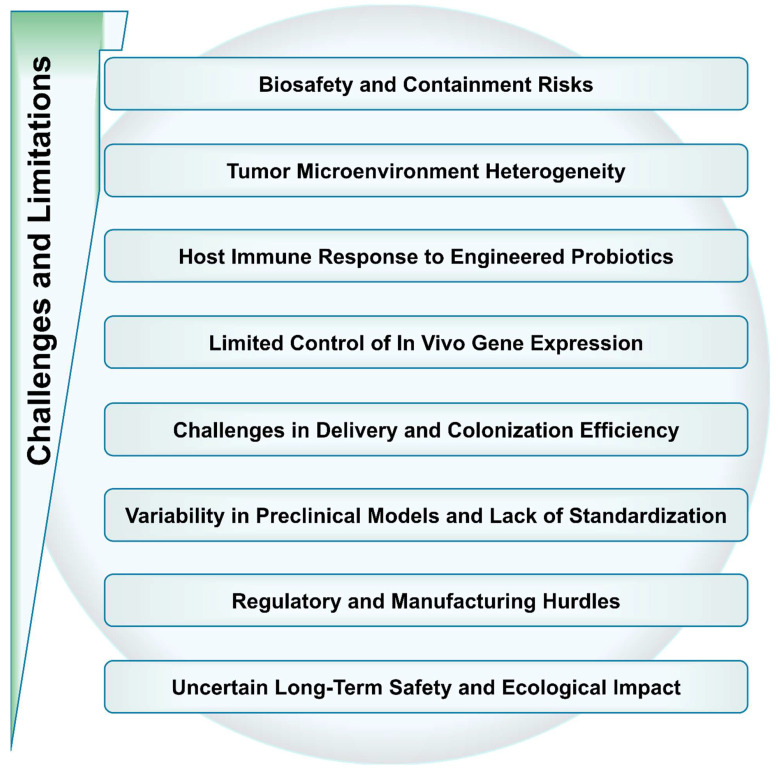
This illustration shows the various challenges and limitations.

**Figure 27:**
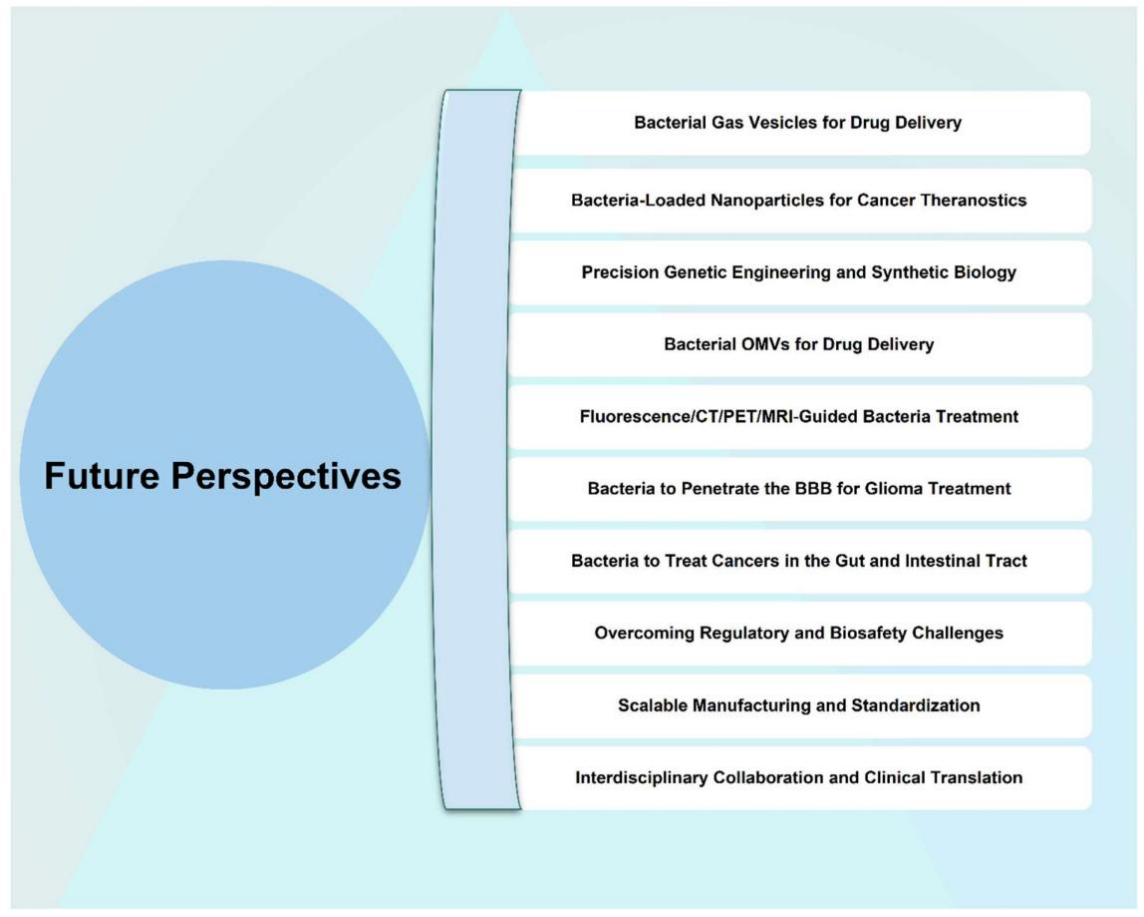
This illustration highlights the future directions for the use of probiotics against the cancer.

**Table 1:** Clinical studies of bacterial-based cancer therapies.

Bacterial strain	Condition	Study phase	Recruitment status	NCT Number	Ref.
Live intestinal bacteria	All Solid Tumors	Phase II/Phase III	Active, not recruiting	NCT03686202	52
Live BCG bacteria	Non-Muscle Invasive Bladder Cancer	Phase III	Recruiting	NCT06241755	53
*Clostridium* novyi-NT spores	Solid Tumor Malignancies	Phase I	Completed	NCT01924689	54
VNP20009	Cancer	Phase I	Completed	NCT00004988	55
*Clostridium* novyi-NT spores	Tumors	Phase I	Terminated	NCT00358397	56
*Clostridium* novyi-NT	Malignant Neoplasm	Phase I	Active, not recruiting	NCT03435952	57
BacTRL-IL-12	Cancer-Solid Tumors	Phase I	Terminated	NCT04025307	58
LBP (Live bacteria product)	Non-small Cell Lung Cancer/Melanoma/Renal Cell Carcinoma	Phase I	Active, not recruiting	NCT05354102	59
MRx0518	Pancreatic Cancer	Phase I	Terminated	NCT04193904	60
